# The sequence, structure and evolutionary features of HOTAIR in mammals

**DOI:** 10.1186/1471-2148-11-102

**Published:** 2011-04-16

**Authors:** Sha He, Shiping Liu, Hao Zhu

**Affiliations:** 1 Bioinformatics Section, School of Basic Medical Sciences, Southern Medical University, Guangzhou, 510515, China; 2 School of Biology, South China University of Technology, Guangzhou, 510510, China

## Abstract

**Background:**

An increasing number of long noncoding RNAs (lncRNAs) have been identified recently. Different from all the others that function *in cis *to regulate local gene expression, the newly identified HOTAIR is located between HoxC11 and HoxC12 in the human genome and regulates HoxD expression in multiple tissues. Like the well-characterised lncRNA Xist, HOTAIR binds to polycomb proteins to methylate histones at multiple HoxD loci, but unlike Xist, many details of its structure and function, as well as the *trans *regulation, remain unclear. Moreover, HOTAIR is involved in the aberrant regulation of gene expression in cancer.

**Results:**

To identify conserved domains in HOTAIR and study the phylogenetic distribution of this lncRNA, we searched the genomes of 10 mammalian and 3 non-mammalian vertebrates for matches to its 6 exons and the two conserved domains within the 1800 bp exon6 using Infernal. There was just one high-scoring hit for each mammal, but many low-scoring hits were found in both mammals and non-mammalian vertebrates. These hits and their flanking genes in four placental mammals and platypus were examined to determine whether HOTAIR contained elements shared by other lncRNAs. Several of the hits were within unknown transcripts or ncRNAs, many were within introns of, or antisense to, protein-coding genes, and conservation of the flanking genes was observed only between human and chimpanzee. Phylogenetic analysis revealed discrete evolutionary dynamics for orthologous sequences of HOTAIR exons. Exon1 at the 5' end and a domain in exon6 near the 3' end, which contain domains that bind to multiple proteins, have evolved faster in primates than in other mammals. Structures were predicted for exon1, two domains of exon6 and the full HOTAIR sequence. The sequence and structure of two fragments, in exon1 and the domain B of exon6 respectively, were identified to robustly occur in predicted structures of exon1, domain B of exon6 and the full HOTAIR in mammals.

**Conclusions:**

HOTAIR exists in mammals, has poorly conserved sequences and considerably conserved structures, and has evolved faster than nearby HoxC genes. Exons of HOTAIR show distinct evolutionary features, and a 239 bp domain in the 1804 bp exon6 is especially conserved. These features, together with the absence of some exons and sequences in mouse, rat and kangaroo, suggest *ab initio *generation of HOTAIR in marsupials. Structure prediction identifies two fragments in the 5' end exon1 and the 3' end domain B of exon6, with sequence and structure invariably occurring in various predicted structures of exon1, the domain B of exon6 and the full HOTAIR.

## Background

Consistent with pervasive transcription of the genome [1,2], many noncoding RNAs (ncRNAs) have recently been discovered. In addition to abundant microRNAs (reviewed recently in [3,4]), an increasing number of long noncoding RNAs (lncRNAs) have been identified, and their crucial functions have been experimentally confirmed. One key aspect of their functions is tissue-specific genome modification [5,6]. After a cell is fully differentiated, many genes are specifically silenced by polycomb proteins and lncRNA-mediated histone methylation rather than by a large army of negative transcriptional factors [7]. Another important aspect is genomic imprinting [8]. One typical example is the Xist-mediated inactivation of the whole X chromosome. Because diverse tissue-specific histone methylation and gene silencing are performed by only a handful of polycomb proteins [9], the great enigma of genome modification is how a few polycomb proteins dynamically and accurately target specific DNA sequences. The discovery of a large amount of lncRNAs should provide key information.

The most studied case of genome modification is X inactivation, the silencing of the majority of genes on one of the X chromosomes in somatic cells to balance gene copy number during mammalian embryogenesis [10,11]. X inactivation is mediated by the lncRNA Xist [12,13], and its details have recently been elucidated [14,15]. Regarding gene silencing and dosage compensation apart from the X chromosome, several lncRNAs, including HOTAIR, play essential roles. HOTAIR is co-expressed with the HoxC genes, interacts with polycomb proteins and functions *in trans *to repress HoxD expression [16,17]. In addition to creating and maintaining spatiotemporally patterned HoxD expression in multiple tissues during embryogenesis, HOTAIR is also involved in aberrant gene expression in cancers [18]. The recently discovered functions of Xist, HOTAIR and other lncRNAs suggest the hypothesis that numerous lncRNAs should exist to bridge the limited number of polycomb proteins and the diverse tissue-specific genome modification. Moreover, many of them should be evolutionarily conserved.

Tiling arrays are widely used to discover new transcripts, especially new ncRNAs [19]. Although this method is convenient and powerful, it can only uncover noncoding transcripts expressed at particular times in particular cells. If functional domains of an lncRNA, such as Xist or HOTAIR, interact with polycomb proteins, they are likely to be conserved in animals and possibly shared by other lncRNAs. This pattern of conservation allows computational genome analysis to be used to identify new lncRNAs and their functional domains, as has been successfully performed for microRNAs [20]. The origin and evolution of these lncRNAs are also of great importance and interest, but they have so far hardly been addressed, except for Xist [21,22]. In this study, we computationally investigated HOTAIR, the first lncRNA shown to function *in trans*. Specifically, we investigated the following questions: (1) whether HOTAIR exists in all mammals or vertebrates, (2) whether it has functional domains shared by other known or potential ncRNAs and whether they are evolutionarily conserved, (3) the evolutionary features of HOTAIR, and (4) the possible structures of its functional domains. We addressed the first question using Infernal, a structure-based RNA homology search program [23], to search the genomes of 13 vertebrates with exons of HOTAIR. We addressed the second question by thoroughly evaluating all of the hits in five animals. We addressed the third question using Paml and EvoNC to analyze sequences orthologous to HOTAIR exons [24,25]. Finally, we addressed the fourth question using PMmulti and Mfold to predict the structures of HOTAIR exons and the full HOTAIR sequences [26,27]. Our results indicated that orthologues of HOTAIR existed only in mammals and that HOTAIR has evolved faster than the neighbouring HoxC genes. Moreover, HOTAIR exons showed discrete evolutionary dynamics, with some having evolved significantly faster in primates. Hits of exons as a whole, with high and low scores, were poorly conserved in animals, except between closely related species. Many hits fell within introns of, or were antisense to, protein coding genes. A comparison of all the predicted 2 dimensional (2D) structures of exon1 and the two conserved domains of exon6 revealed two invariable fragments in these structures. These results uncovered multiple facets of HOTAIR and the implications of our results within the wide range of lncRNA evolution and function are discussed.

## Methods

### Data

The sequence of human HOTAIR was obtained from the National Center for Biotechnology Information (NCBI) database (accession number NR_003716.2). The unmasked genome data (Ensembl database version 57) of human (GRCh37.p2, Feb. 2009), chimpanzee (CHIMP2.1, Mar. 2006), rhesus monkey (MMUL 1.0, Feb. 2006), gorilla (gorGor3, Dec. 2009), cow (Btau_4.0, Oct. 2007), horse (Equ Cab2, Sep. 2007), dolphin (turTru1, Jul. 2008), dog (CanFam2.0, May 2006), mouse (NCBI m37, Apr. 2007), rat (RGSC 3.4, Dec. 2004), platypus (Ornithorhynchus_anatinus-5.0, Dec 2005), chicken (WASHUC2, May 2006), and zebrafish (Zv9, Apr. 2010) were downloaded from Ensembl. The sequences corresponding to the rat orthologue of HOTAIR exon6 (consisting of two domains of HOTAIR exon6 that are conserved in mammals) and the sequences corresponding to the short exon of human HoxC12 aligned by Multiz against 22 mammals (human, chimpanzee, rhesus monkey, gorilla, hedgehog, dog, rat, mouse, dolphin, elephant, orangutan, baboon, guinea pig, rabbit, cow, horse, marmoset, kangaroo, armadillo, hyrax, lemur, and platypus) were obtained from the UCSC Genome Browser database [28].

### Obtaining sequences orthologous to the HOTAIR exons

Each of the 6 exons of human HOTAIR was used as a query to search the genomes of rhesus monkey (rheMac2, Jan. 2005) and dog (Broad/canFam2, May 2005) in Ensembl using BLAT [29]. The sequences orthologous (best hit) to each exon, except exon6, in human, rhesus monkey and dog (exon2 and exon5 did not have good hits in dog) were aligned using PMmulti (v1.6, [30]); the sequences orthologous to exon6 were aligned using LocARNA (v1.5.4, [31]). With the 6 alignment results, 6 queries (query1 to query6) were built using the *cmbuild *and *cmcalibrate *functions of Infernal (v1.0.1, [23]) and then used to search the whole genomes of 13 vertebrates using the *cmsearch *function of Infernal. Two short domains of exon6, the ~235 bp domain A and the ~239 bp domain B, were identified in orthologues of exon6 in all 10 mammals. For the orthologous sequences of the two domains in human, rhesus monkey and dog, two additional queries (query6a and query6b) were built using the *cmbuild *and *cmcalibrate *functions of Infernal. They were then used to search the whole genomes of the 13 vertebrates using the *cmsearch *function of Infernal.

### Sequence alignment and structure prediction

Sequences of the 10 mammals that were orthologous to HOTAIR exon6 were aligned using LocARNA, and sequences orthologous to all of the other HOTAIR exons from the 10 mammals were aligned using PMmulti for phylogenetic analysis. Structures were predicted for the orthologues of exon1, exon6 domain A and exon6 domain B using PMmulti and Mfold (http://mfold.rna.albany.edu/?q=mfold, [26,27]), and the predicted structures were displayed using either Mfold or PseudoViewer (v2.5, [32]). In all cases, default parameters were used unless otherwise indicated.

### Phylogenetic analysis

Using orthologous sequences of HOTAIR exon6 and the concatenated homogeneous sequences of exon1, exon3, exon4 and exon5 in the 10 mammals, two phylogenetic trees were built using the *dnadist *and *kitsch *functions of Phylip (v3.69, [33]). Phylogenetic analysis of the two trees was performed using the *baseml *function of Paml (v4.4, [24]). Fixed parameters included *model *= 4 (the HKY85 nucleotide substitution model); *fix_kappa *= 0 and *kappa *= 2; *fix_alpha *= 0 and *alpha *= 0.5; *ncatG *= 5, *fix_rho *= 1 and *rho *= 0; and *cleandata *= 0. The parameters *kappa *(κ), *alpha *(α)*, local clock *and *rates of substitution *were estimated under different conditions [34-37]. The evolution of the two conserved domains of exon6 and the short exon of the neighbouring HoxC12 gene was analyzed in 22 mammals using EvoNC [25].

## Results

### The sequences of orthologous HOTAIR exons are poorly conserved

**Figure 1:**
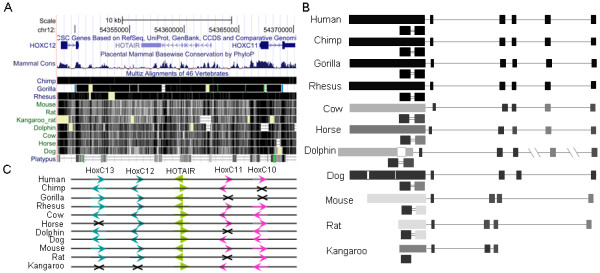
**Sequence conservation of HOTAIR orthologues in mammals**. (A) The sequences of HOTAIR orthologues are obviously conserved in primates but less well conserved in other animals (from UCSC Genome Browser). (B) Orthologues of the HOTAIR exons exist only in mammals. Exon1, exon3, exon4, exon5 and domain B of exon6 are better conserved than exon2, exon6 and domain A of exon6 (indicated by the darkness of the boxes). Note that the sequence of the exon6 orthologue is significantly shorter in rat than in other mammals and contains just two domains. The two boxes under each exon6 are domain A (right side) and domain B (left side), linked by a double line indicating a gap of 130 bp (unmatched part in the Infernal search). The gaps in exon6 of dolphin and dog also indicate unmatched parts in the Infernal search. The double slashes in the schematic of the dolphin gene indicate long introns. (C) The order and orientation of HOTAIR and its neighbouring HoxC genes in mammals. X: HoxC is absent.

lncRNAs that function *in cis*, including Xist, AIR and Kcnq1ot1 [38-41], should evolve closely with their nearby target genes. HOTAIR is the first lncRNA that has been found to function *in trans *to regulate remote gene expression; it is co-expressed with the HoxC genes on chromosome 12 and down-regulates HoxD genes on chromosome 2 in particular tissues in human [16]. Because this regulation occurs between distant genomic loci, whether it exists in other mammals and non-mammalian vertebrates is of great interest. We first searched several mammalian genomes in the UCSC and Ensembl databases for matches to HOTAIR exons using Blastn and BLAT [29]. Close matches were found in primates but not in other mammals. For example, although the whole sequence of HOTAIR showed apparent conservation among mammalian orthologues (Figure 1A), individually, the five short exons (exon1 to exon5) returned few hits from mammalian genomes. Using BLAT, only exons 1, 3 and 4 produced hits in dog and only exon4 produced hits in cow between HoxC11 and HoxC12, and all of these hits had poor scores. This finding suggests that if HOTAIR has orthologues in mammals and other vertebrates, they may show low sequence conservation. This lack of conservation is not a surprise because compensatory mutations occur widely in ncRNAs, and many ncRNAs are conserved in structure but not in sequence [42,43].

### HOTAIR exists only in mammals

Because ncRNAs are characterised by divergent sequences and conserved structures, to further address the question of whether HOTAIR exists in mammals and other non-mammalian vertebrates, we used Infernal to search whole genomes for matches to HOTAIR exons. Infernal is a local RNA alignment and search tool based on structure conservation [23]. To make the covariance model necessarily representative, we chose rhesus monkey, a primate that is more distantly related to human than chimpanzee, and dog, a mammal that produced several hits in the BLAT search. First, we identified sequences orthologous to HOTAIR exons in rhesus monkey and dog; these sequences were BLAT search hits with high scores and successive locations between HoxC11 and HoxC12. Second, we used the sequences of six human HOTAIR exons and their orthologues in rhesus monkey and dog to build six queries using the *cmbuild *and *cmcalibrate *functions of Infernal. Using these queries, we searched the genomes of 10 placental mammals (human, chimpanzee, rhesus monkey, gorilla, cow, horse, dog, dolphin, mouse and rat), the ancestral mammal platypus, and 2 other vertebrates (chicken and zebrafish). Orthologues of the HOTAIR exons (hits located between HoxC11 and HoxC12 with high scores) were obtained in all of the placental mammals but not in platypus or the other vertebrates (Figure 1B and Table S1 in Additional file 1). Notably, each query produced just one high-scoring hit in the mammalian genomes. These hits were located between HoxC10/HoxC11 and HoxC12/HoxC13 (HoxC11 or HoxC12 is absent in some mammals, Figure 1C), and all of the other hits had low scores. Query2 did not produce any high-scoring hits in dog, mouse or rat. Moreover, query6 produced good matches in primates but poor matches in other mammals, especially in mouse and rat (Figure 1B, Table S1 in Additional file 1, and Additional file 2). These results suggest that HOTAIR exists only in mammals and that, after some evolutionary process, it became highly conserved in primates.

The downloaded genome data were released in Ensembl and UCSC at the same time, except that the platypus data were released in Ensembl in Dec 2005 and in UCSC in Mar 2007. To check if different assemblies affect genome search result, we downloaded the platypus genome data from UCSC and repeated the Infernal search. The obtained results were basically the same as those obtained from the platypus data in Ensembl.

### Fragments of HOTAIR exons are widely found in mammalian and non-mammalian vertebrate genomes

Except for one high-scoring hit located between HoxC11 and HoxC12, low-scoring hits of short queries (110 bp to 120 bp for query1 to query4 and 64 bp for query5) were widely obtained in mammalian and other vertebrate genomes. These hits matched a fraction of a HOTAIR exon and it is unclear whether they contained any functional element. However, the 1,804 bp query6 produced few low-scoring hits in mammals and other vertebrates. Because the best hit was less conserved in non-primate mammals and much shorter in mouse and rat (Figure 1B), we inferred that the functional domain(s) conserved in mammals should be much shorter than 1,804 bp. Further searches addressed this issue. The rat orthologue of exon6 was only 622 bp, which was separated in the middle by an unmatched gap of 130 bp. In mouse, there was a similar gap of 150 bp at the same position. This gap, therefore, divided the highly conserved initial 622 bp of query6 into two domains (Figure 1B). We extracted the two domains from the human, rhesus monkey and dog genomes and built query6a and query6b, respectively, as described above. As expected, searches of the 13 genomes with query6a and query6b produced more hits, but no new high-scoring hits were obtained. This result suggests that the two domains of exon6, which could be the backbone of HOTAIR, are not shared by other lncRNAs. While orthologues of domains A and B of exon6 were equally conserved in primates, orthologues of domain A were much less conserved in other mammals, especially in rodents (Figure 1B). Thus, the two domains may undergo different evolutionary processes or dynamics. Query6a and query6b also produced some hits with moderate or low scores. Many of these hits matched to either two specific fragments in query6a (from approximately 50 bp to 100 bp and from 130 bp to 180 bp) or a specific fragment in query6b (from approximately 160 bp to 210 bp). Whether these fragments are essential parts of the two domains and whether they are functional in vertebrates are unclear. Using lifeOver in UCSC, we checked whether hits show syntenic relationships among animals, and found that the coordinates of many hits, possibly in non-annotated regions, cannot be converted.

### Hits of queries show distinct distributions in genomes

Experimental studies have revealed that both HOTAIR and Xist bind to Ezh2 [44] and that HOTAIR contains at least two functional domains. The 5' domain binds Suz12, a component of polycomb repressive complex 2 (PRC2), whereas the 3' domain binds LSD1 [17]. Because these proteins, especially the components of PRC2, are bound by many lncRNAs, we speculated that not all of the low-scoring hits were functionally irrelevant. A popular method to roughly determine whether a DNA sequence is functional is to evaluate its conserved context [45-47]. We examined the hits of all of the queries in human, chimpanzee, mouse, rat and platypus. To evaluate the distribution of hits in the genomes and determine if they were flanked by genes with the same annotation, for each query and each genome, we counted the following: (a) the total number of hits, (b) the number of hits within introns, (c) the number of hits within novel transcripts or known ncRNAs, (d) the number of hits in exons of protein coding genes, (e) the number of hits antisense to a gene, (f) the number of hits in intergenic regions, and (g) the number of hits in the 3'UTR or 5'UTR of genes (Table 1). Essentially, no hits fell within exons of protein coding genes, and a majority of the hits were intergenic. Nevertheless, a few observations should be noted. First, no hits were found within Xist. Second, some hits fell within novel transcripts or known ncRNAs, highlighting the possibility that there could be functional elements in these transcripts or ncRNAs. Third, many hits fell within introns of protein coding genes. Finally, although query4 was the same length as query1, query2 and query3 and query5 was even shorter, in all mammals, query4 and query5 produced significantly fewer hits. This high variance cannot be accounted for simply by random hits. One potential explanation is that exon1, exon2 and exon3 may contain functional elements that are shared by other ncRNAs and/or distributed more widely.

**Table 1:** Distribution of hits of queries in human, chimpanzee, mouse, rat and platypus

	human		chimp		mouse		rat		platypus	
Query1	79a		84a		62a		42a		28a	
	13b	1c	18b	0c	10b	1c	12b	0c	3b	0c
	0d	22e	0d	16e	0d	15e	0d	5e	0d	2e
	42f	1g	50f	0g	37f	0g	25f	0g	23f 0g	

Query2	90a		83a		69a		68a		103a	
	21b	1c	15b	0c	24b	0c	10b	0c	9b	0c
	0d	26e	0d	19e	0d	12e	2d	7e	1d	17e
	40f	2g	49f	0g	32f	1g	49f	0g	76f	0g

Query3	56a		60a		111a		119a		125a	
	11b	2c	8b	0c	23b	0c	10b	0c	1b	0c
	0d	12e	0d	8e	0d	13e	1d	14e	0d	16e
	31f	0g	44f	0g	75f	0g	94f	0g	108f	0g

Query4	17a		14a		10a		14a		12a	
	3b	0c	1b	0c	3b	0c	3b	0c	0b	0c
	0d	5e	1d	4e	0d	3e	0d	2e	0d	2e
	9f	0g	8f	0g	4f	0g	9f	0g	10f	0g

Query5	13a		16a		27a		14a		43a	
	4b	0c	3b	0c	2b	0c	0b	0c	5b	0c
	0d	3e	0d	3e	0d	6e	0d	1e	0d	0e
	6f	0g	10f	0g	19f	0g	13f	0g	38f	0g

Query6	15a		17a		10a		14a		0a	
	1b	0c	0b	0c	1b	0c	1b	0c		
	0d	2e	0d	0e	0d	2e	0d	0e		
	12f	0g	17f	0g	7f	0g	13f	0g		

Query6	69a		79a		99a		274a		23a	
a	12b	0c	10b	0c	20b	2c	18b	0c	0b	0c
	0d	24e	0d	19e	0d	17e	0d	28e	0d	3e
	32f	1g	50f	0g	59f	1g	126f	0g	20f	0g

Query6	76a		102a		81a		90a		45a	
b	15b	1c	12b	0c	27b	1c	17b	0c	5b	0c
	0d	17e	0f	16e	0d	7e	1d	7e	1d	1e
	42f	1g	74f	0g	45f	1g	65f	0g	38f	0g

a: the total number of hits, b: the number of hits in introns, c: the number of hits in novel transcripts or known ncRNAs, d: the number of hits in exons of protein coding genes, e: the number of hits antisense to a gene, f: the number of intergenic hits, g: the number of hits in the 3'UTR or 5'UTR of genes.

Flanking genes in different animals often reflect the evolutionary conservation of a DNA sequence. We specifically examined the flanking genes of each hit of query1, query6a and query6b. Query1 had 32 hits flanked by the same genes in human and chimpanzee but just 1 hit flanked by the same gene in mouse and rat. Moreover, no hit of query1 was flanked by the same genes in all four mammals. Query6a had 14 hits flanked by the same genes in human and chimpanzee, but none in mouse and rat. Consistent with high conservation, query6b had 21 hits flanked by the same genes in human and chimpanzee but none in mouse and rat. As mouse and rat have an evolutionary distance (divergence time) at least 4 times that of human and chimpanzee [37], these results indicate that hits of these queries have moderately conserved distributions in mammalian genomes.

### Orthologous sequences of HOTAIR exons show different evolutionary dynamics

**Figure 2:**
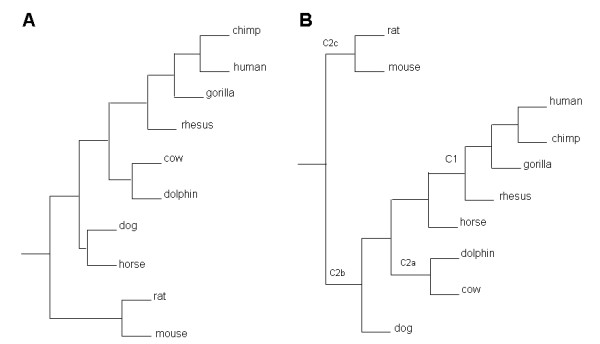
**Phylogeny of HOTAIR**. (A) A tree built with concatenated sequences of orthologues of exon1, exon3, exon4 and exon5. (B) A tree built with sequences of exon6 orthologues. C1 indicates the first local clock, while C2a, C2b and C2c indicate the second local clock inserted at three different places in different computations.

Most protein coding genes are produced by gene duplication followed by neofunctionalisation and/or subfunctionalisation. Because an increasing number of ncRNAs have been identified, the mechanisms through which these ncRNAs form and evolve are of great interest. HOTAIR comprises five short and one long exon. Although its origin remains obscure, some exons are apparently less conserved than others. We therefore analyzed the molecular evolution of the HOTAIR exons. Using the concatenated sequences orthologues to exon1, exon3, exon4 and exon5 and the sequences orthologous to exon6, we built two phylogenetic trees using Phylip [33] (Figure 2). Assuming that nucleotide substitutions followed the HKY85 model [34] and rates of nucleotide substitutions varied among sites, we analyzed sequences of HOTAIR exons using Paml. We first compared the two trees under multiple conditions. Under all of the conditions examined, nearly the same log-likelihood (lnL) and other parameters were obtained for the two trees. For example, if nucleotide substitution rates were variable among sites and the molecular clock was allowed to vary from branch to branch, tree A produced a slightly larger log-likelihood (-759.53 vs. -774.33) for exon1 but slightly smaller log-likelihoods for the other exons (-13949.07 vs. -13940.2 as the summed values). Because exon6 is the main body of HOTAIR, we then chose tree B to perform evolutionary analysis. We examined whether nucleotide substitution rates varied among sites in the exons using the log-likelihood ratio test, a statistical test for comparing two models [35]. The smallest 2ΔlnL = 2((-11774.82)-(-11791.17)) = 32.7 was obtained from orthologous sequences of exon6 (-11774.82 for the HKY85+gamma model and -11791.17 for the HKY85 model). The probability distribution of the test can be approximated by a chi-square distribution with one degree of freedom, with  = 7.88, supporting the model of disparate nucleotide substitution rates. Further analysis revealed that the sequences of the orthologues of different exons had different transition/transversion rate ratio (*κ*), different shape parameter of the gamma distribution (*α*), and different nucleotide substitution rates between clades (Table 2). Because exon1, exon2 and exon6 had significant *α*>1, most sites in these exons should have moderate substitution rates, but a few sites had fast or slow rates of substitution. Because exons 3, 4 and 5 all had *α *< 1, most sites in these exons should have low substitution rates. In addition, the values of α in exon6 (*α *>1 for domain A and *α *< 1 for domain B) indicated that domain B was more conserved than domain A, which agrees with the Infernal results in which the scores of the hits to query6a in non-primate mammals were lower than the scores of hits to query6b (Figure 1B and Table S1 in Additional file 1). These results indicate asynchronous evolution of orthologous sequences of HOTAIR exons in mammals.

**Table 2:** Some estimated parameters of molecular evolution

	Exon1	Exon2	Exon3	Exon4	Exon5	Exon6	DomainA	DomainB
*κ*	1.81901	2.88324	4.85122	1.92371	5.90266	2.18681	1.50398	1.45864

*α*	233.20*	137.621	0.66662	0.66759	0.70397	3.14532	2.04029	0.70016

r_1_/r_2a_	9.34444	1.88055	0.46272	2.23894	1.06430	1.06613	0.43880	2.78380

	7.19374	0.83409	0.46463	0.99973	1.78446	1.16327	0.45130	0.92838

r_1_/r_2b_	6.65571	0.89836	1.00285	1.01670	0.46319	0.47190	0.55024	2.92695
	0.39720	0.42971	0.71267	0.45403	0.59854	0.47959	0.74729	1.02247

*r*_*1*_*/r*_*2c*_	16.75779	2.09060	1.40709	2.23928	0.77386	0.98396	0.73568	2.86161
#	0.45376	0.26278	0.03274	0.11204	0.21409	0.47428	0.74774	0.39388

To obtain stable local clock estimations, only two local clocks were specified in each run (see Figure 2B) and the remaining species had rate r_0 _= 1. *: a large unstable value. #: r_2c _was not stable because the clade (mouse, rat) was too close to the root.

To examine HOTAIR evolution in more detail, we also investigated whether nucleotide substitution rates varied among clades. First, we performed a log-likelihood ratio test to determine whether the HKY85 model would fit the data better with or without a global clock. The smallest 2ΔlnL = 2((-11774.82)-(-11814.58)) = 79.52, with = 21.96 (this log-likelihood ratio test has eight degrees of freedom), was obtained from orthologous sequences of exon6 (-11774.82 for the HKY85+gamma model without a global clock and -11814.58 for the HKY85+gamma model with a global clock). This result clearly disproved the global clock hypothesis. Then, we set two local clocks to investigate whether the exons evolved at different rates in mammals (Figure 2B). For exon1, exon2, exon4 and domain B of exon6, the substitution rates in primates were significantly higher than those in the other group of mammals; for exon3, exon5 and domain A of exon6, the substitution rates were not much different between the two groups (Table 2). As the 5' domain of HOTAIR has been found to bind to Suz12 and the 3' domain to LSD1 [17], whether the accelerated evolution of exon1, exon2 and domain B of exon6 in primates has relationship with their protein binding function awaits further investigation. In addition, the frequencies of nucleotide substitutions (πA, πT, πC, πG) varied significantly for different branches and at different nodes. Taken together, these results suggest that HOTAIR may be a relatively new gene, with some exons having recently undergone an accelerated evolution.

### HOTAIR evolves faster than its neighbouring HoxC genes in mammals

HoxC genes exist widely in vertebrates; HOTAIR, in contrast, exists only in mammals. It was therefore interesting to determine whether HOTAIR evolved faster than the neighbouring HoxC genes. Because HoxC11 is absent in rat, dolphin and platypus and the long exon of human HoxC12 is absent in some mammals, we compared the evolution of the short exon of HoxC12 with the evolution of the main part of exon6 of HOTAIR in 22 mammals (see Data and Methods). Sequences from the UCSC database that were aligned by Multiz and EvoNC, a program for detecting selection in noncoding regions of nucleotide sequences, were used [25]. For protein coding sequences, the rate of nonsynonymous/synonymous substitution was used to detect selection pressure and positive/negative selection. To apply such detection to noncoding sequences, the rate of substitution relative to the rate of synonymous substitution in coding sequences can be modelled by the parameter δ. δ = 1 indicates that a site in a noncoding sequence evolved neutrally, whereas δ < 1 and δ > 1 suggest positive and negative selection, respectively [25]. We concatenated the aligned orthologous region of HOTAIR and the aligned short exon of HoxC12 and analyzed the concatenated sequences. The results are shown in Table 3. The log-likelihood test clearly rejected the null hypothesis that the HOTAIR region evolved neutrally, and the value of 4.1694 found for δ_2 _in the three-category case strongly suggested that the HOTAIR region was under positive selection and evolved faster than HoxC12. The exact driving force behind this positive selection remains to be elucidated.

**Table 3:** Log-likelihood values and parameter estimates given by EvoNC

	Likelihood	Κ	ω	**δ**_**0**_	***p***_***0***_	**δ**_**1**_	***p***_***1***_	**δ**_**2**_	***p***_***2***_
Neutral	-3665.64	3.74	0.60	0.0834	0.6070	1.00	0.3930		

Two category	-3650.17	3.37	0.60	0.2964	0.6439	3.3412	0.3561		

Three category	-3645.42	3.48	0.60	0.0362	0.3571	1.00	0.4358	4.1694	0.2071

### Structure prediction reveals two domains with invariable sequences and structures

As many lncRNAs, including Xist and HOTAIR, can interact with both polycomb proteins and DNA sequences, it is important to identify the sequences and structures of their functional domains [48]. An lncRNA may have a conserved backbone and/or functional domains but have varied structure in different species, making the determination of the accurate structure of the full lncRNA difficult and sometimes unnecessary. So, instead of attempting to predict the structure of the full HOTAIR, we focused on determining the sequence and structure of possible functional domains in its exons. Because the orthologous sequences of each HOTAIR exon were obtained using structure-based genome searches, they had the same structures as the queries built by PMmulti and Infernal. Because each query produced only one high-scoring hit located between HoxC11 and HoxC12, the structures of queries determined by PMmulti and Infernal should be reasonable. To facilitate the determination of the sequence and structure of possible functional domains, two constraints were used. First, in the consensus structure of each query determined by PMmulti and used by Infernal, functional domains should be occupied by sequences conserved in the 10 mammals. Second, in all of the possible structures of an exon's orthologue in a mammal predicted by other tools, functional domains should have invariant sequences and structures.

**Figure 3:**
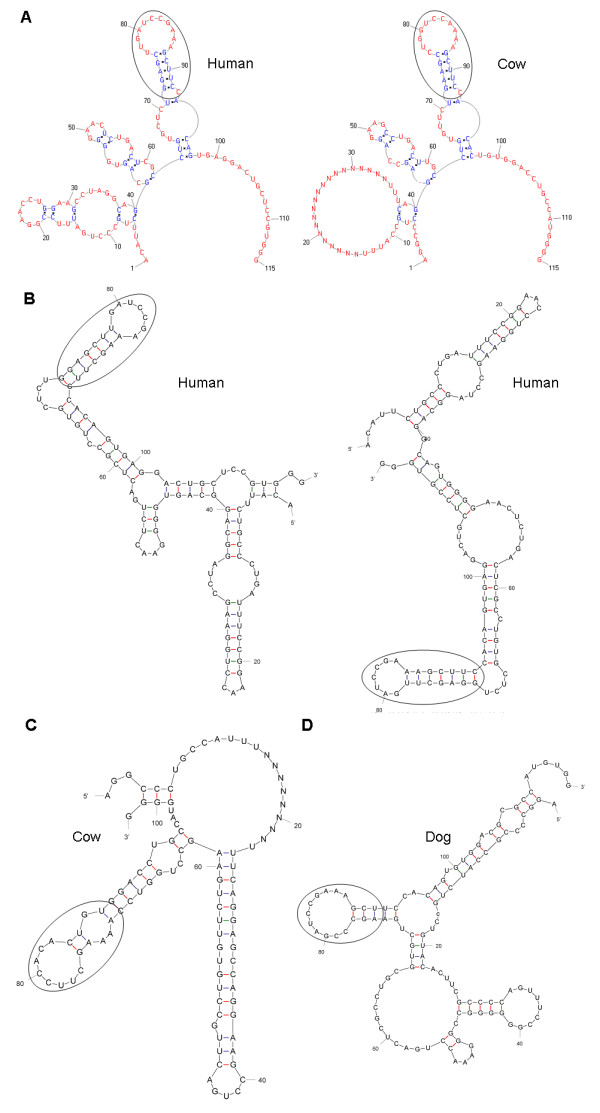
**Predicted structures of exon1 orthologues in mammals**. (A) The structure predicted by PMmulti and used by Infernal. This consensus structure consists of one big arc and three substructures. In some mammals, the bottom substructure contains three small loops, but in cow, dolphin, mouse and rat, it is a big loop. The middle substructure contains three tiny loops and the top substructure contains a hairpin at its end in all animals. (B) Two structures predicted by Mfold in human. Although the overall structures are different, the hairpin structure found in the PMmulti-predicted structure invariably occurs in both structures. (C) Two structures predicted by Mfold: one in cow and one in dog. The sequence and its hairpin structure (slightly varied) occur in both structures.

Because a 5' domain of HOTAIR binds PRC2 [17], we assumed the 5' domain should be conserved in mammals and query2 did not produce any high-scoring hits in dog, mouse or rat, we tried to identify the functional domain in predicted structures of exon1. PseudoViewer shows that the consensus structure for exon1 consists of one big arc and three substructures (Figure 3A and Figure S1 in Additional file 1). The bottom substructure contains three small loops in some mammals, but is a large loop in cow, dolphin, mouse and rat; therefore it is unlikely to be a functional domain. The middle substructure contains three tiny loops and the top substructure contains a hairpin at its end in all animals, which indicates that they could be or contain the functional domain. To obtain more results to aid in the determination, Mfold was used to predict structures of exon1's orthologous sequence in each mammal [27]. Mfold predicted 9 structures in human, 9 in chimpanzee, 14 in rhesus monkey, 3 in gorilla, 2 in cow, 3 in dog, 2 in dolphin, 42 in horse, 1 in mouse and 3 in rat (Figure S2 in Additional file 1). Notably, the hairpin was found at the same position in 7 of the 9 predicted structures in human, and in the other 2 cases its sequence was embedded within neighbouring sequences (Figure 3BCD and Figure S2 in Additional file 1). Similar results were obtained from other animals. These Mfold predicted structures provide valuable and complementary information for determining the possible position and structure of the functional domain in exon1.

**Figure 4:**
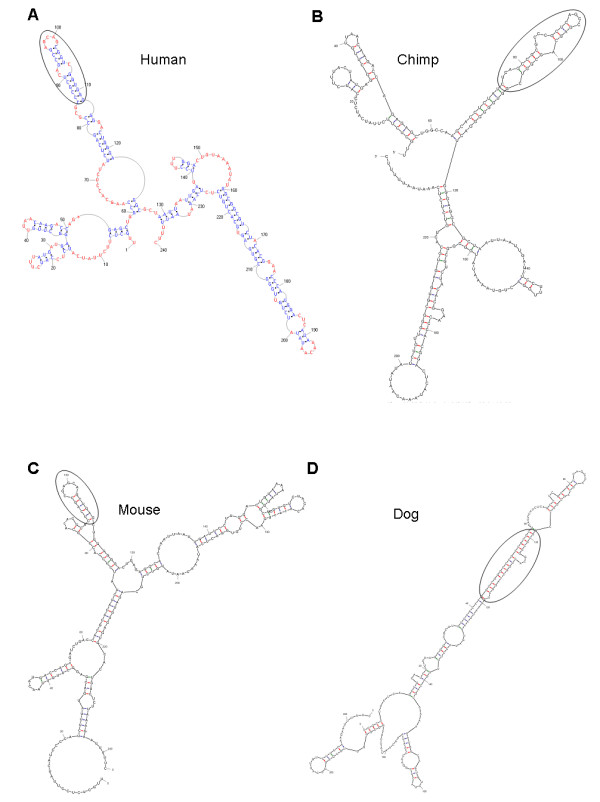
**Predicted structures of orthologues of domain B of exon6 in mammals**. (A) The structure predicted by PMmulti and used by Infernal. The circled part was identified by comparing the structure with the structures predicted by Mfold based on the position and base pairing of sequence. (B) One structure predicted by Mfold in chimpanzee; the circled part is nearly the same as that predicted for the human sequence. (C) One structure predicted by Mfold in mouse; the circled part is slightly different but still occurs at one end. (D) One structure predicted by Mfold in dog; the circled part is embedded within other sequences.

According to an experimental study, a 3' domain of HOTAIR, located from approximately 1500 bp to 2146 bp, binds LSD1 [17]. However, Infernal produced short sequences for the exon6 orthologues in mouse and rat (1,500 bp and 622 bp, respectively), which did not include the 3' end reported in the human sequence. Postulating that the 3' functional domain should be conserved in mammals and might not be as long as 622 bp, we analyzed the structures of domain A (560 bp to 800 bp) and domain B (950 bp to 1,190 bp) of exon6 in the 10 mammals. As stated previously, the structure determined by PMmulti and used by Infernal was compared with all of the structures predicted by Mfold. We first examined domain B. Mfold predicted 3 structures for domain B in human, 6 in chimpanzee, 6 in rhesus monkey, 8 in gorilla, 2 in cow, 13 in dog, 5 in dolphin, 7 in horse, 9 in mouse and 7 in rat. We first checked those in human and rat, the two mammals with the greatest evolutionary distance, and found that a conserved GC-rich paired fragment existed in structures predicted for all 10 mammals and that it closely matched the marked part (the circled area) in the structure predicted by PMmulti (Figure 4A and Figure S3B in Additional file 1). We then examined all 66 structures of domain B predicted by Mfold for the 10 mammals and found that in 4 cases, the GC-rich paired fragment had the structure shown in Figure 4A (human), in 39 cases it had the structure shown in Figure 4B (chimpanzee), in 11 cases it had the structure shown in Figure 4C (cow), and in 7 cases it had the structure shown in Figure 4D (dog). Compared with the predicted functional domain in exon1, this specific structure existed more obviously in domain B. In contrast, domain A of exon6 was much less conserved (Figure 1B and Table S1 in Additional file 1) and was GC poor (data not shown), without a clear consensus substructure in structures predicted using Mfold.

### The sequence and structure of the two domains occur nearly invariably in structures of full HOTAIR

Although focusing on the sequence and structure of conserved (and potentially functional) domains is reasonable, the structure of a piece of RNA can be very different from that when it is embedded by long sequences. To validate the sequence and structure of the two conserved fragments in exon1 and domain B of exon6, we predicted structures of the full HOTAIR in all the mammals. The predicted sequence and structure of the fragment in exon1 occurs in many structures of full HOTAIR; remarkably, the predicted sequence and structure of the fragment in domain B of exon6 occurs in most structures of full HOTAIR. For example, Mfold produced 29 and 37 structures for human and rat full HOTAIR respectively. In humans, the predicted structure of the fragment in exon1 occurs in 8 of 29 full HOTAIR structures, and the predicted structure of the fragment in domain B of exon6 occurs in 20 of 29 full HOTAIR structures. In rats, the predicted structure of the fragment in exon1 occurs in 33 of 37 full HOTAIR structures, and the predicted structure of the fragment in domain B of exon6 occurs in 31 of 37 full HOTAIR structures (Additional file 3). Given the length of HOTAIR and the number of its predicted structures, these results strongly support the predicted functional fragment in domain B of exon6. The next step should be to experimentally validate these structures and their functions.

## Discussion

Except for Xist, the origin, evolution, structure and phylogenetic distribution of lncRNAs have barely been investigated. Because BLAT failed to find orthologous sequences of HOTAIR exons in mammals, some exons are missing in some mammals and gaps exist in many mammals in the sequences of exon orthologues identified using the RNA homology search software Infernal, HOTAIR is likely to have conserved structures but divergent sequences. This feature should be common to lncRNAs rather than being unique to HOTAIR [N1,N2]. For example, XIST contains both rapid evolving sequences and highly conserved domains [49]. What constrains lncRNAs evolution is poorly understood. As they interact with both the PRC2 complex and specific DNA sequences, co-evolution with specific DNA sequences should be an important aspect. Compared with lncRNAs functioning *in cis *to regulate local genes, the evolutionary constraints of HOTAIR that function *in tran *is more intriguing. Because the Infernal search produced just one high-scoring hit in each placental mammal, where this was located between HoxC11 and HoxC12, it can be inferred that HOTAIR exists in eutherians and that it is younger than its neighbouring Hox genes. How HOTAIR originated remains unclear. Phylogenetic analysis revealed that within the relatively young gene, HOTAIR exons had asynchronous evolutionary dynamics and some exons had undergone an accelerated evolutionary process in primates. These results indicate positive selection during HOTAIR's evolution. Accelerated evolution is also supported by the comparison between HOTAIR exon6 and the short exon of HoxC12, which clearly showed that the HOTAIR exon evolved significantly faster than the HoxC12 exon. Structure prediction for the orthologous sequences in 10 mammals showed two fragments in exon1 and domain B of exon6 with invariant base pairing and 2D structure (Figure 3 and Figure 4). These fragments, located at the 5' end and close to the 3' end of HOTAIR, respectively, could be functional domains of HOTAIR.

One query based on a HOTAIR exon produced only one high-scoring hit in the genome of each mammal, where this was located between HoxC11 and HoxC12, the location of HOTAIR in the human genome. However, many low-scoring hits were found in other places in mammalian and other vertebrate genomes. Because a considerable number of lncRNAs are believed to interact with polycomb proteins to conduct tissue-specific genome modification, we anticipated, for several reasons, that the Infernal search would identify some consensus sequences for polycomb protein binding in genomes, like the TATA box in promoters and the homeobox in Hox genes, that are shared by other lncRNAs. First, the four families of Hox genes have demonstrated complex cross regulation and compensation during embryogenesis [50,51], which suggests that multiple HOTAIR-like lncRNAs may be needed to mediate negative feedback and dosage balances among Hox genes. Second, Hox genes participate in diverse cell fate determination and reprogramming [52,53], which suggests that Hox-related lncRNAs may mediate genome modification at multiple loci. Third, a recent study revealed that both HOTAIR and the RepA ncRNA within Xist bind to the PRC2 complex [44], although it is unclear whether the binding domains are similar. Moreover, because multiple important proteins, such as Nanog, Oct4 and Sox2, also interact with Xist [54], the scope of lncRNA functions should be large. These facts make it theoretically plausible that there should be many lncRNAs that share the same or similar functional domains with HOTAIR. However, except for one high-scoring hit, no hits with moderate scores were obtained. To what extent lncRNAs maintain conserved function with evolved sequences is poorly understood. It is unlikely that all of the low-scoring hits are random hits, because query4 and query5, which are equal to and shorter than query1, produced much fewer hits. In addition, some low-scoring hits fell within novel transcripts and unknown ncRNAs, and many were within introns of or antisense to protein coding genes (Table 1), which is consistent with the findings that many lncRNAs (like AIR and Kcnq1ot1) are antisense to protein coding genes [11,55]. Global transcriptome analysis has revealed that a large proportion of the genome can produce transcripts from both strands [2] and antisense transcription is believed to have roles in gene regulation. Meanwhile, more than 55,000 completely intronic noncoding RNAs have been found to be transcribed from the introns of 74% of all unique RefSeq genes, which indicates that RNAs transcribed from intronic regions of genes have distinct regulatory roles and are involved in a number of processes [56,57]. To carefully compare all hits with cDNA libraries should produce more information.

The evolution of lncRNA sequences, including those within the vertebrate Hox clusters, has been examined recently. These studies reveal that the evolution of many lncRNAs is not consistent with the neutral evolution model, and purifying selection has acted on their promoters and some conserved sites [58,59]. However, except for Xist [21,22], the evolution of specific ncRNA genes has not been examined. Compared with ancestral regions and general intergenic sequences, lncRNA sequences have been shown to exhibit lower rates of nucleotide substitution, insertion, and deletion, which can be interpreted to indicate that they have undergone purifying selection [58]. Our analysis of orthologues of HOTAIR in 10 mammals covering multiple eutherian orders suggests that HOTAIR exons have discrete evolutionary dynamics, and that some exons evolved significantly faster in primates than in non-primate mammals. The analysis of orthologous sequences of HOTAIR exon6 and a HoxC12 exon in 22 mammals indicates that HOTAIR may have evolved faster than its neighbouring HoxC genes. These results suggest that HOTAIR may have undergone an accelerated evolution in eutherians under positive selection. In general, a gene with important function should evolve slowly due to strong functional constraints; however the opposite is often true when the gene is young (in an active neofunctionalisation or subfunctionalisation stage). For example, young proteins experience more variable selection pressures than established proteins [60]. That HOTAIR is not found in non-mammalian vertebrates and that it has evolved faster than nearby HoxC genes both indicate that it is a young gene that formed after the two rounds of whole genome duplication. Given that most lncRNAs, including Xist, have so far only been found in mammals, it is interesting to ask when and why these lncRNAs emerge in higher vertebrates to mediate genome modification. Because HOTAIR exists in mammals, it evolves faster than HoxC12, and its exons have discrete evolutionary dynamics, we postulate that HOTAIR may have formed *ab initio*, possibly via the activity of transposons. HOTAIR is involved in the PRC2-mediated silencing of chromatin at HoxD loci, but its main effect in the regulation of Hox gene expression may be dosage compensation. In this sense it is similar to Xist. The lower effectiveness of dosage compensation in birds than in mammals and the lack of general dosage compensation for sex-linked genes in chickens [61,62] may explain why HOTAIR, like Xist, is found only in eutherians.

In this study, the structures of HOTAIR exons were predicted using two programs. Without any experimental data for structure prediction [48], we adopted a comparative computational approach to predict the sequence and structure of conserved functional domains of HOTAIR rather than the structure of the full HOTAIR sequence in mammals. PMmulti and Mfold were used to predict multiple potential structures for orthologues of each exon. For example, for exon1, Mfold predicted 9 structures in human, 9 in chimpanzee, 14 in rhesus monkey, 3 in gorilla, 2 in cow, 3 in dog, 2 in dolphin, 42 in horse, 1 in mouse and 3 in rat. If invariant sequence base pairing and 2D structure are found in all of the structures predicted using Mfold and in the consensus structure predicted using PMmulti, it is highly likely that the sequence and structure represents a functional domain. Because to produce experimental data to determine structures of lncRNAs is sophisticated and time-consuming, the results of our structure prediction should be valuable for further experimental studies of HOTAIR, and the methods should be applicable to studies of other lncRNAs.

## Conclusions

The lncRNA HOTAIR has poorly conserved sequences and considerably conserved structures in 11 examined mammals (10 eutherians and 1 marsupial). It shows distinct evolutionary features and has evolved faster than nearby HoxC genes. Given that exons 1-5 are very short, exon1 and a domain of exon6 (1804 bp) are absent in kangaroo, and exon2 is absent in mouse, rat and kangaroo, a highly conserved 239 bp domain in exon6, initially appeared in kangaroo, should be the backbone of HOTAIR. These findings suggest the *ab initio *generation of HOTAIR in marsupials. Structure prediction identifies two fragments, in the 5' end exon1 and the 3' end domain B of exon6 respectively, with their sequence and structure invariably occurring in various predicted structures of exon1, the domain B of exon6 and the full HOTAIR. These are supported by experimental findings. To compare the origin and evolutionary features of HOTAIR with Xist suggests that many lncRNAs, may first form in marsupials and then have undergone a rapid evolution in eutherians. An interesting question is whether their origin and evolution is intrinsically associated.

## Note added in proof

During the review of the manuscript, we downloaded, searched and analyzed opossum and kangaroo genome data (Ensembl released the improved assemblies of kangaroo (dipOrd1.60) and opossum (BROADO5.57) in Oct 2010). Whole genome search of opossum did not produce high-scoring hits. Searching the kangaroo genome with query3, query4, query5 and query6 each produced a high-scoring hit, which have successive addresses in GeneScaffold_2370 (Figure 1B and Additional file 2). Query6's hit matches query6 from 33 bp to 655 bp, exactly as that in rat. What is interesting is that exon1 and exon2 were absent, domain B of exon6 produced a high-scoring hit, and domain A of exon6 was not identified. These results, together with the phylogenetic analysis, lead to two suggestions. First, HOTAIR, like other lncRNAs, first formed in some marsupials and underwent a rapid evolution in eutherians. Second, domain B of exon6 may be the backbone of HOTAIR, because it is the only relatively long piece conserved in marsupial and eutherians.

Monotremes have multiple X chromosomes but it is not clear whether they undergo dosage compensation; marsupials show dosage compensation but they lack Xist [63]. It is found that female marsupials may use an ancestral dosage compensation mechanism that differs from, but share common properties with, the Xist based in eutherians [64,65]. Since protein-coding genes that flank the eutherian XIC are well-conserved in M. domestica and vertebrates and there is a surprising break in synteny with eutherian mammals and other vertebrates, it is suggested that during the evolution of the marsupial X chromosome, one or more rearrangements broke up an otherwise evolutionarily conserved block of vertebrate genes [66]. The situation of HOTAIR, which is flanked by HoxC11 and HoxC12, not found in vertebrates and initially occurs in marsupials as revealed in this study, seems quite similar to Xist. This raises the interesting question of whether HOTAIR and Xist, and possibly also some other lncRNAs, have undergone the same evolutionary process.

## Abbreviations

ncRNA: noncoding RNA; lncRNA: long noncoding RNA; PRC2: polycomb repressive complex 2.

## Authors' contributions

SH performed genome search, results analysis and structure prediction; SL participated in genome search; HZ conceived the project, performed phylogenetic analysis, structure prediction and wrote the manuscript. All authors read and approved the final manuscript.

## Supplementary Material

Additional file 1 **This file contains Table S1, Figure S1, Figure S2, and Figure S3**. Click here for file

Additional file 2 **This file contains orthologues of HOTAIR exons and their coordinates in mammals**. Click here for file

Additional file 3 **This file contains predicted structures of full HOTAIR in human and rat**. Click here for file
