# Supplementary material for: The sequence, structure and evolutionary features of HOTAIR in mammals
Source: BMC Evol Biol. 2011 Apr 16;11:102. doi: 10.1186/1471-2148-11-102 (PMC3103462; doi:10.1186/1471-2148-11-102)
Supplement: Additional file 1 — This file contains Table S1, Figure S1, Figure S2, and Figure S3. [file 1471-2148-11-102-S1.DOC]

Table S1. Hits with high scores and successive addresses are only found in mammals

|  | Query1  (115bp) | Query2  (126bp) | Query3  (102bp) | Query4  (125bp) | Query5  (64bp) | Query6  (1804bp) | Query6a  (235bp) | Query6b  (239bp) |
| --- | --- | --- | --- | --- | --- | --- | --- | --- |
| Human | Q=1-115  S=123.29 | Q=1-126  S=151.15 | Q=1-102  S=121.17 | Q=125 S=131.54 | Q=1-64 S=71.89 | Q=1-1804, S=2073.52 | Q=1-235  S=270.96 | Q=1-239  S=267.95 |
| Chimp | Q=115 S=123.29 | Q=1-126  S=151.15 | Q=1-102  S=120.99 | Q=1-125  S=129.02 | Q=1-64  S=79.08 | Q=1-1804  S = 2041.98 | Q=1-235  S=271.83 | Q=1-239  S=255.41 |
| Rhesus | Q=1-115  S=122.37 | Q=1-126  S=151.71 | Q=1-102  S=120.97 | Q=1-125  S=133.21 | Q=1-64  S=91.45 | Q=1-1804  S=2071.94 | Q=1-235  S=259.09 | Q=1-239  S=272.97 |
| Gorilla | Q=1-115  S=125.54  (with a small gap) | Q=1-126  151.15 | Q=1-102  S=115.29 | Q=1-125  120.82 | Q=1-64  S=79.08 | Q=1-1804  S=2038.43 | Q=1-235  S=268.66 | Q=1-239  S=261.76 |
| Cow | Q=1-115  S=68.22 | Q=1-126  S=27.39 | Q=1-102  S=91.35 | Q=1-125  S=98.63, | Q=1-64  S=63.15 | Q=1-1804  S=402.43  (with big gaps) | Q=1-235  S=117.54 | Q=1-239  S=168.61 |
| Horse | Q=1-115  S=71.09 | Q=1-126  S=30.59 | Q=1-102  S=72.06 | Q=1-125  S=94.46 | Q=1-64  S=83.02 | Q=1-1804, S=812.78  (with big gaps) | Q=1-235  S=71.61 | Q=1-239  S=177.18 |
| Dolphin | Q=1-115  S=72.45  (with a small gap) | Q=1-126  S=34.21 | Q=1-102  S=89.83 | Q=1-125  S=104.20 | Q=1-64  S=84.06 | Q=11-660  S=183.99  Q=875-1096  S=57.59  Q=1222-1459  S=136.87  Q=1527-1802  S=120.57  (with big gaps) | Q=1-235  S=135.23 | Q=1-239  S=173.43 |
| Dog | Q=1-115  S=117.60 | No | Q=1-102  S=116.57 | Q=1-125  S=116.11 | Q=1-64  S=79.99 | Q=9-662  S=240.17  Q=697-1221, S=112.80  Q=1222-1459  S=140.27  Q=1148-1209  S=43.86  Q=1527-1802  S=43.39  (with big gaps) | Q=1-235  S=77.33 | Q=1-239  S=164.89 |
| Mouse | Q=1-115  S=36.75  (with a big gap) | No | Q=1-102  S=58.52 | Q=1-125  S=63.08 | Q=1-64  S=64.72 | Q=9-1506  S=180.26  (with big gaps) | Q=1-235  S=25.81 | Q=1-239  S=167.58 |
| Rat | Q=1-115  S=26.53  (with a big gap) | No | Q=1-102  S=65.51 | Q=1-125  S=60.42 | Q=1-64  S=66.78 | Q=33-655  S=179.13  (with big gaps) | Q=1-235  31.49 | Q=1-239  S=190.30 |

(Q=query coverage, S=score. The largest E value of these hits is E=0.008724.)


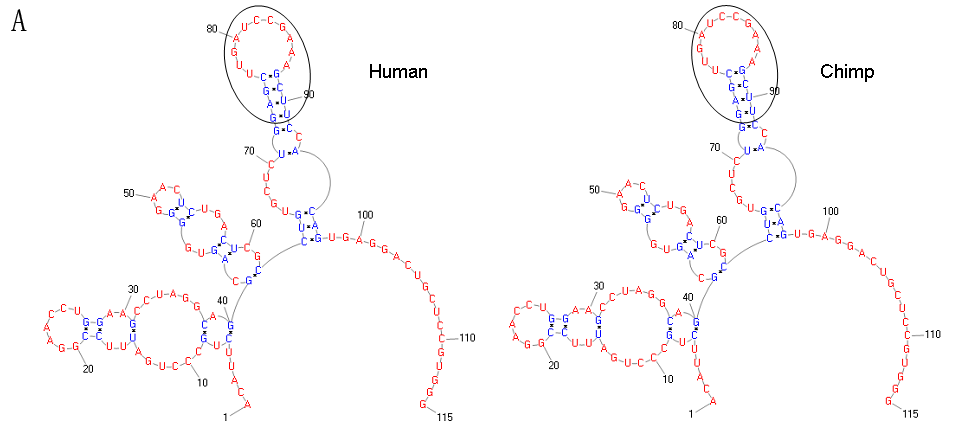


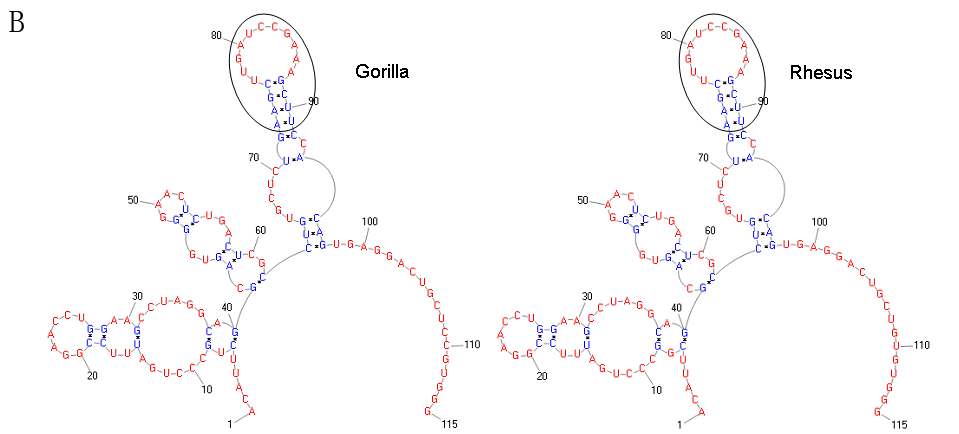


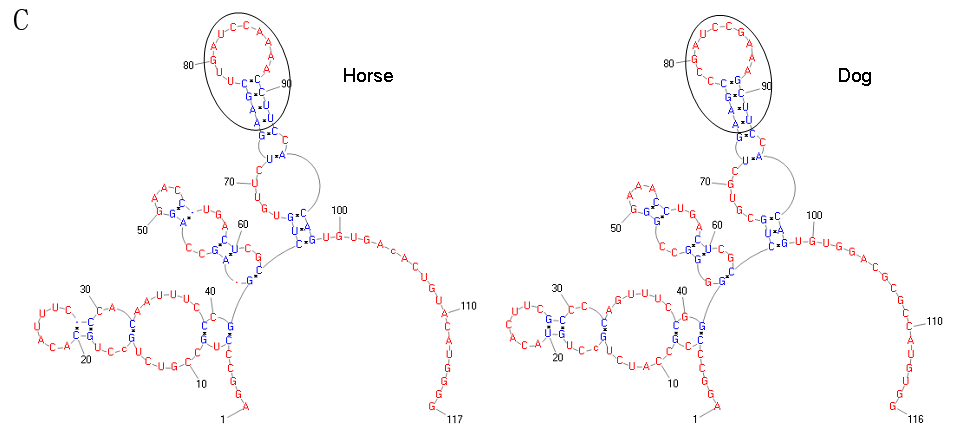


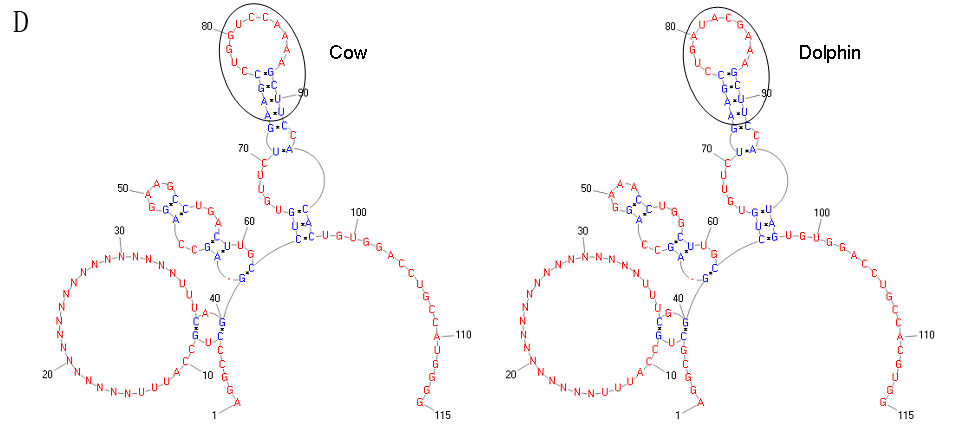


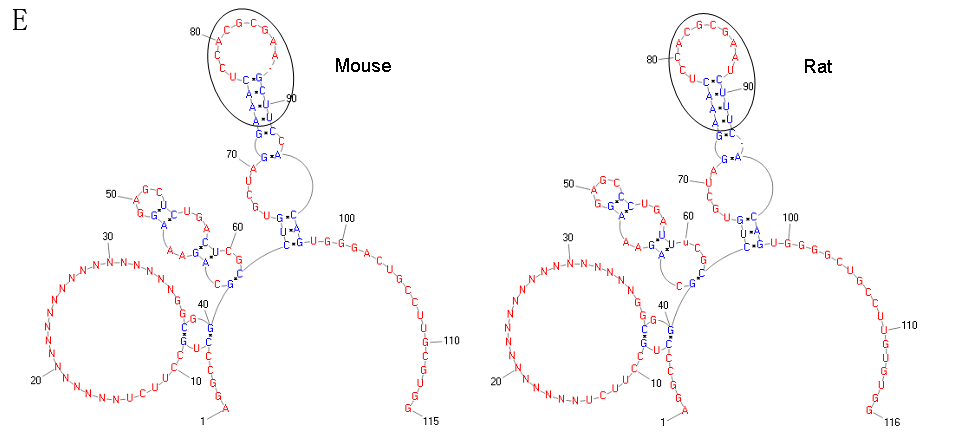


Figure S1 The structure of exon1 in 10 mammals predicted by PMmulti and used by Infernal (A) In human and chimpanzee. (B) In gorilla and rhesus. (C) In horse and dog. (D) In cow and dolphin. (E) In mouse and rat.


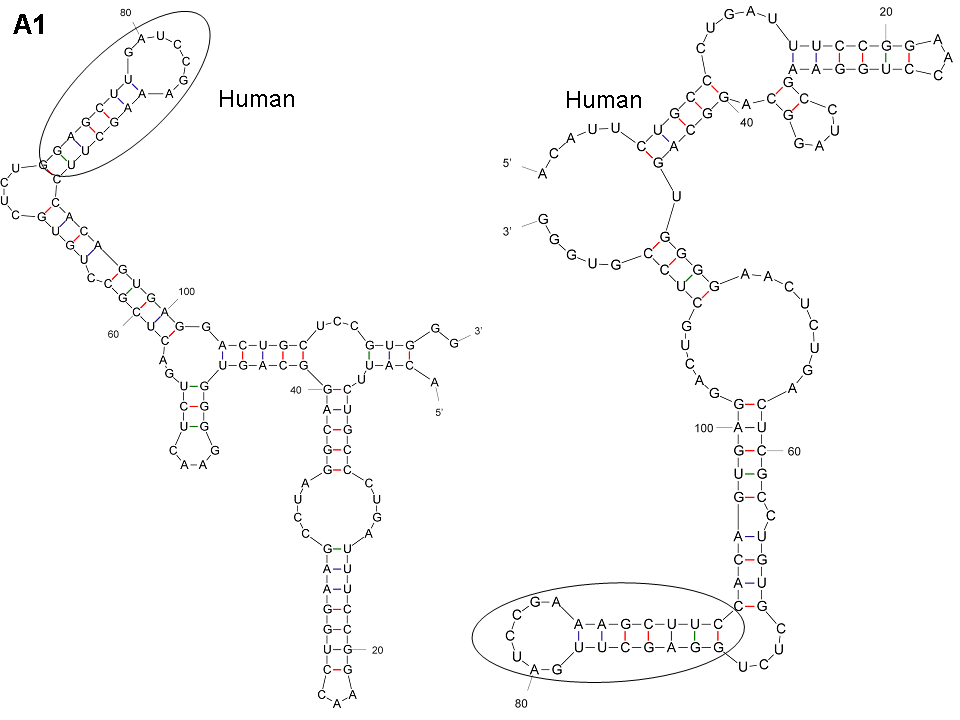

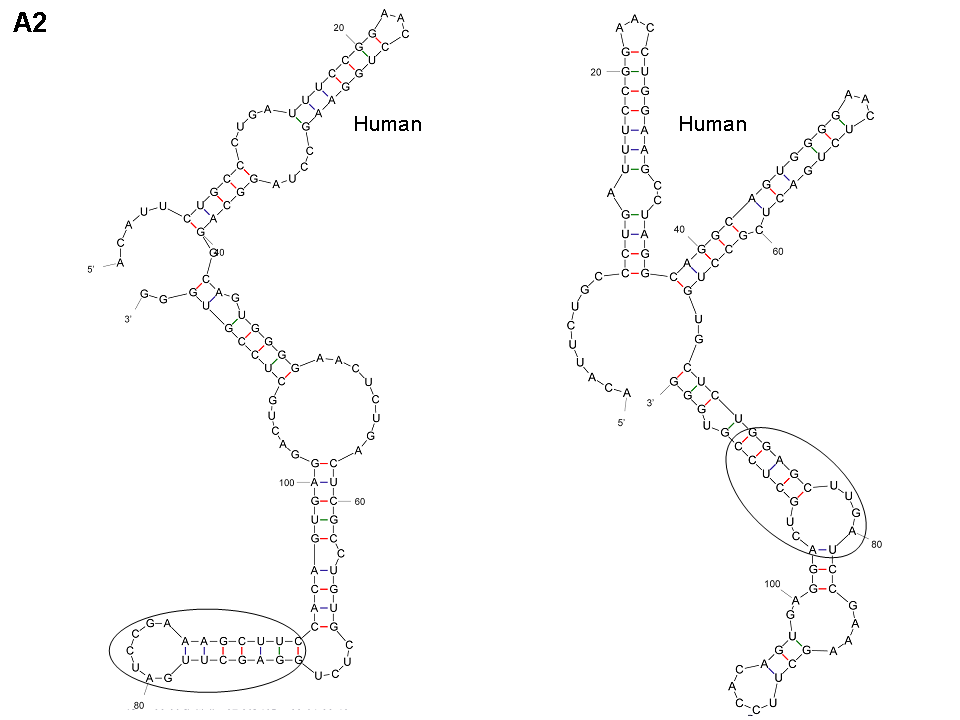

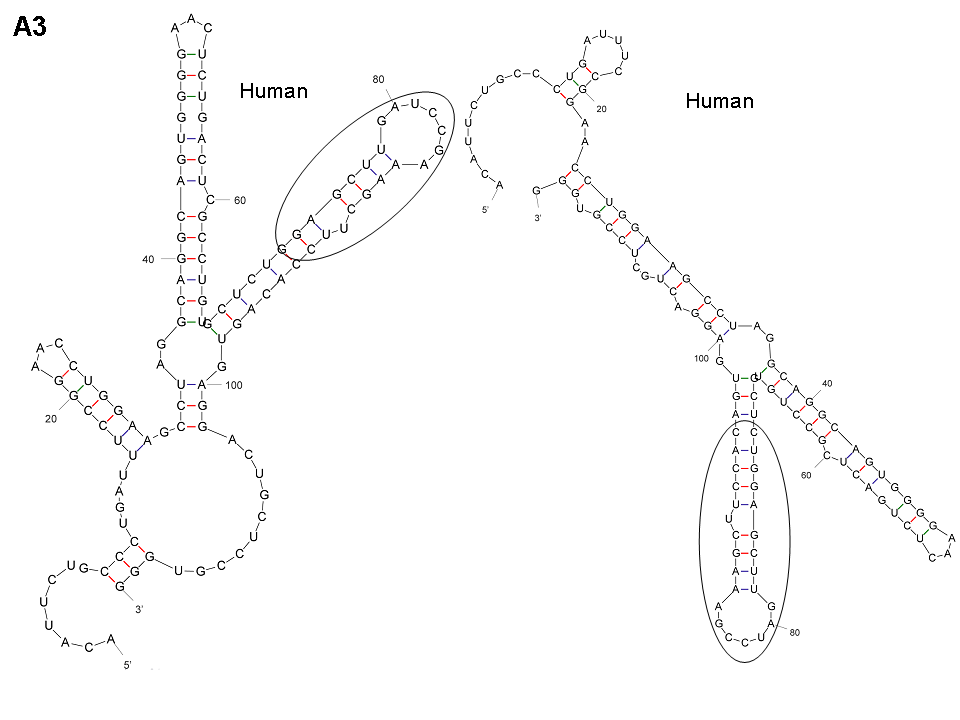

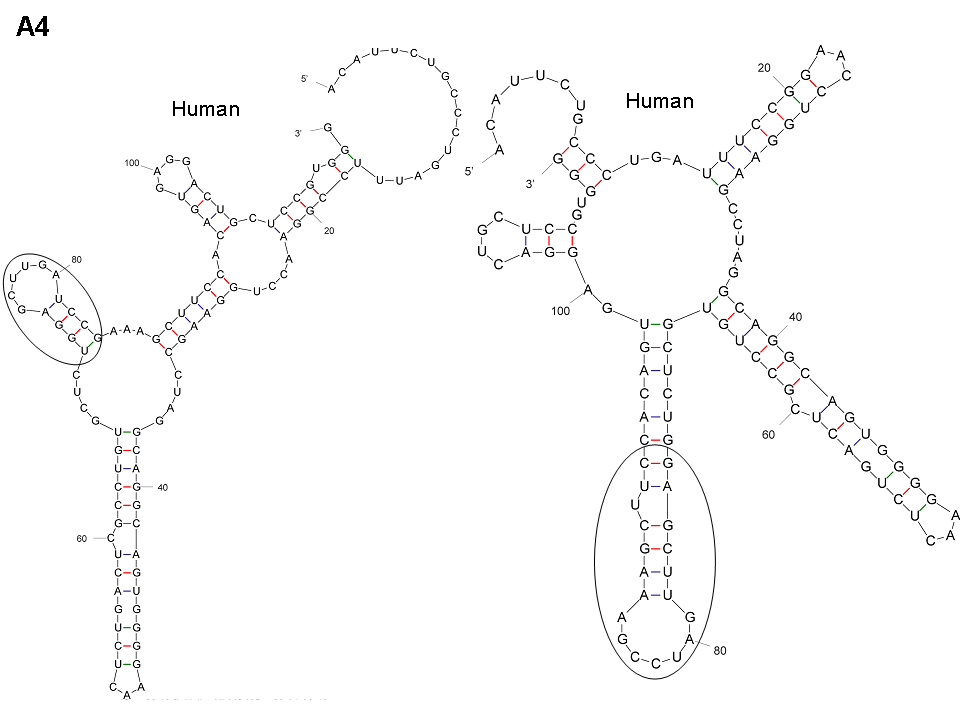

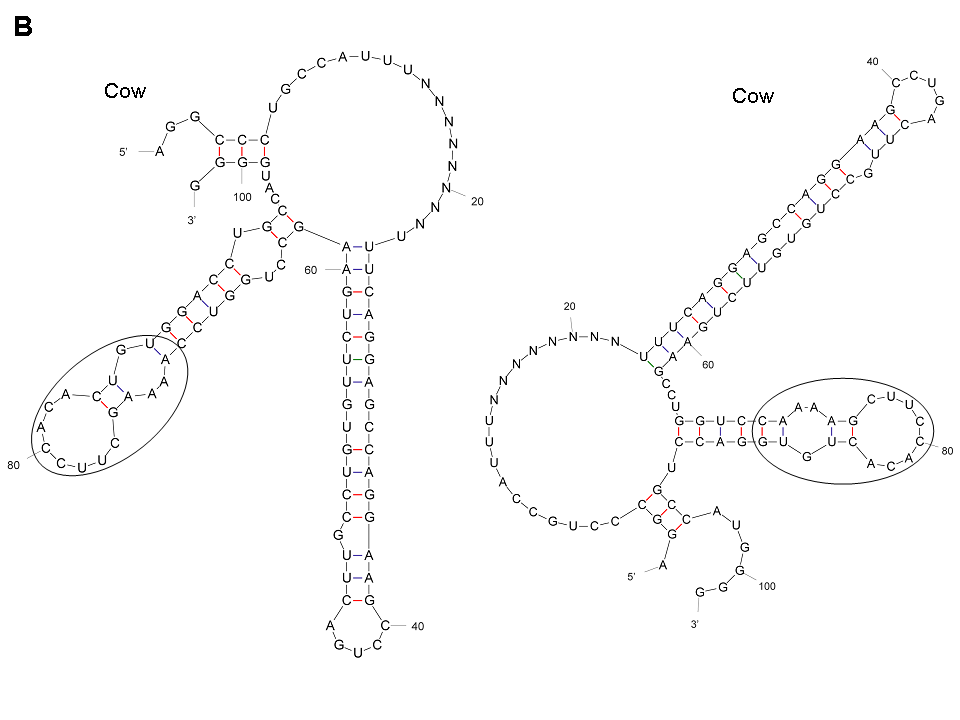

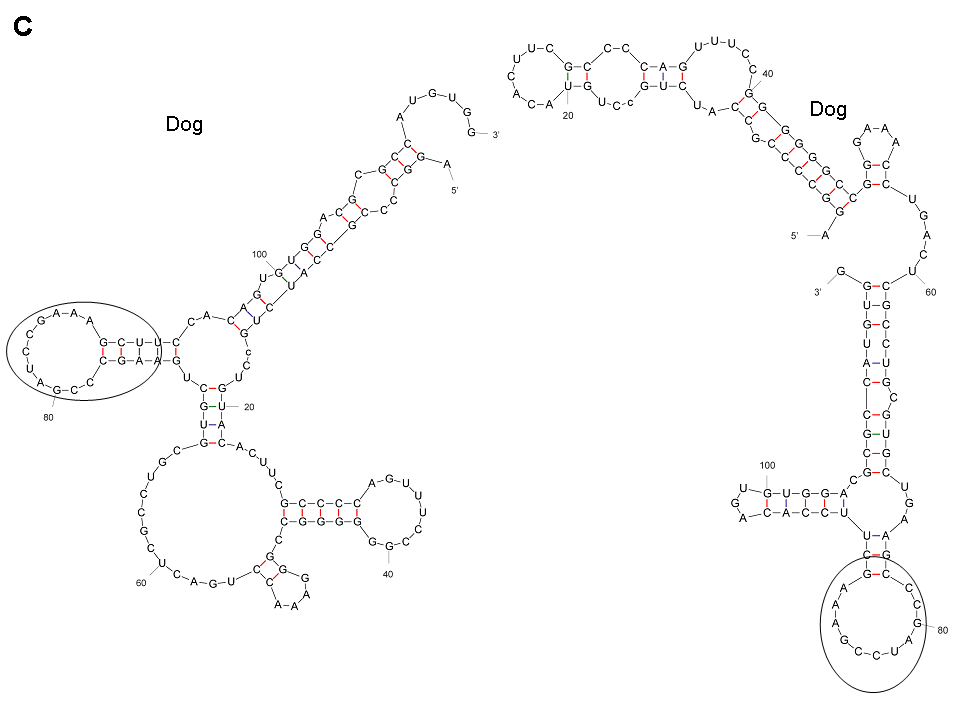

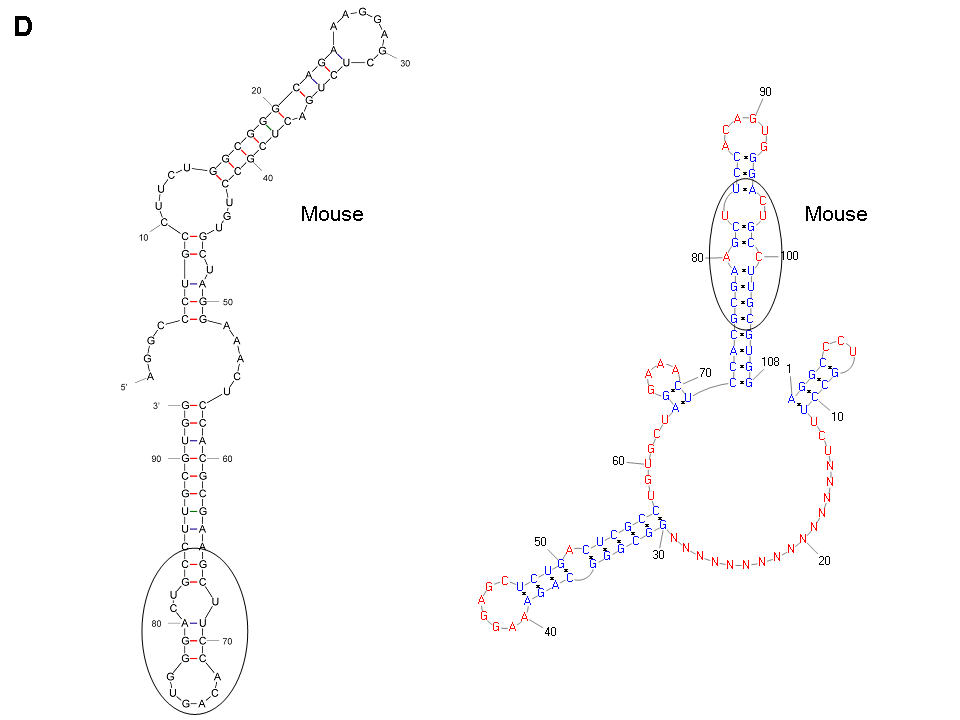


Figure S2. Structures of exon1 predicted by Mfold. (A1-A4) The 8 structures in human. (B) The 2 structures in cow. (C) The 2 structures in dog. (D) The 2 structure in mouse (the right one is displayed by PseudoViewer because there is a defect in Mfold’s display).


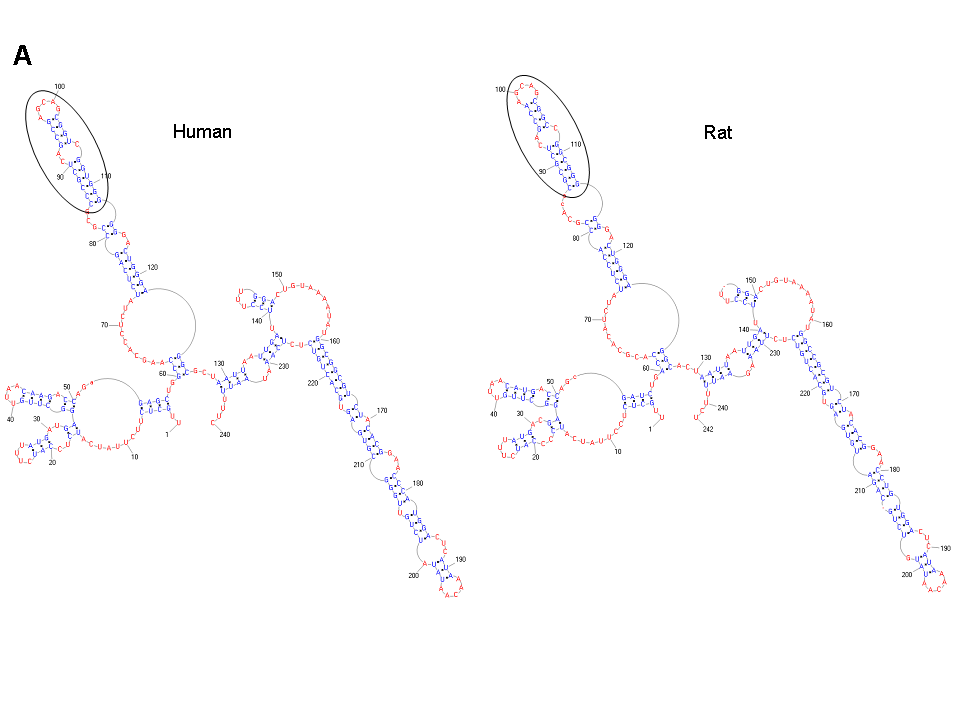


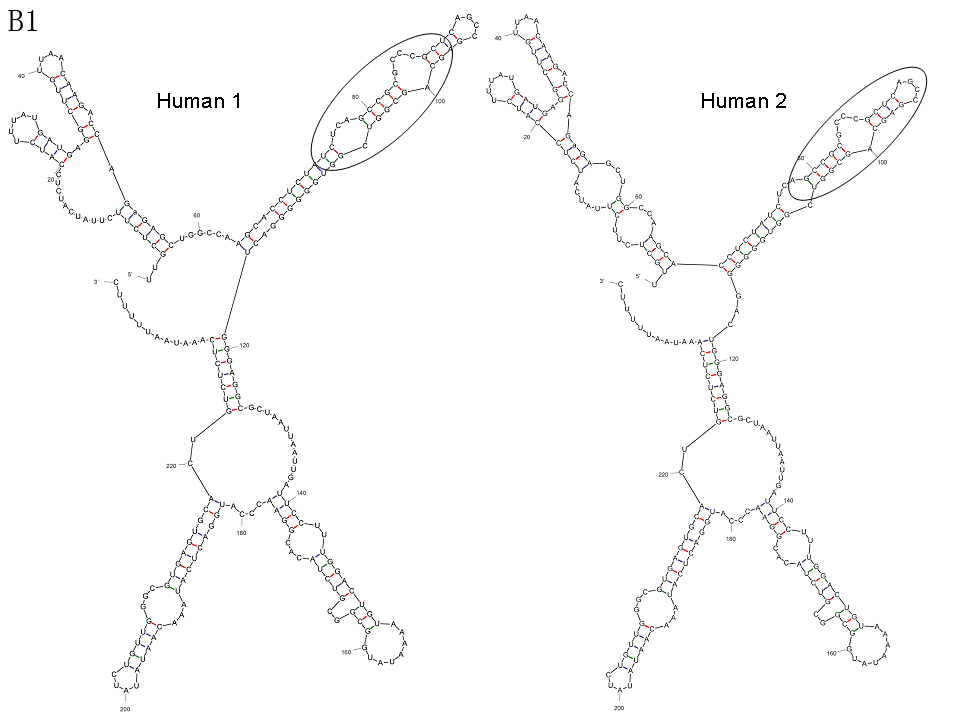


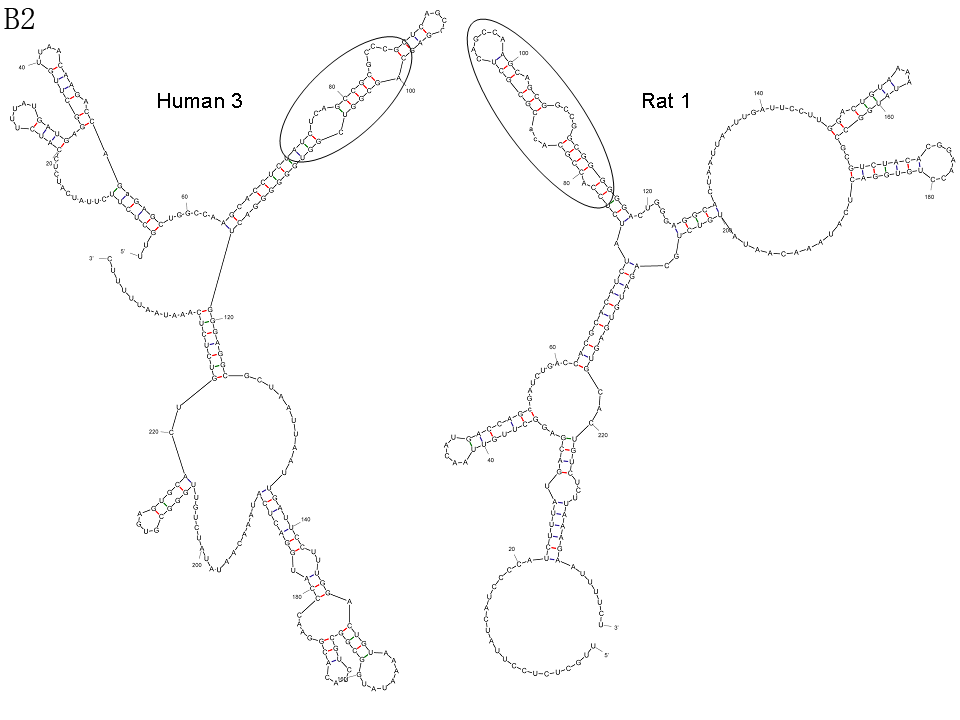


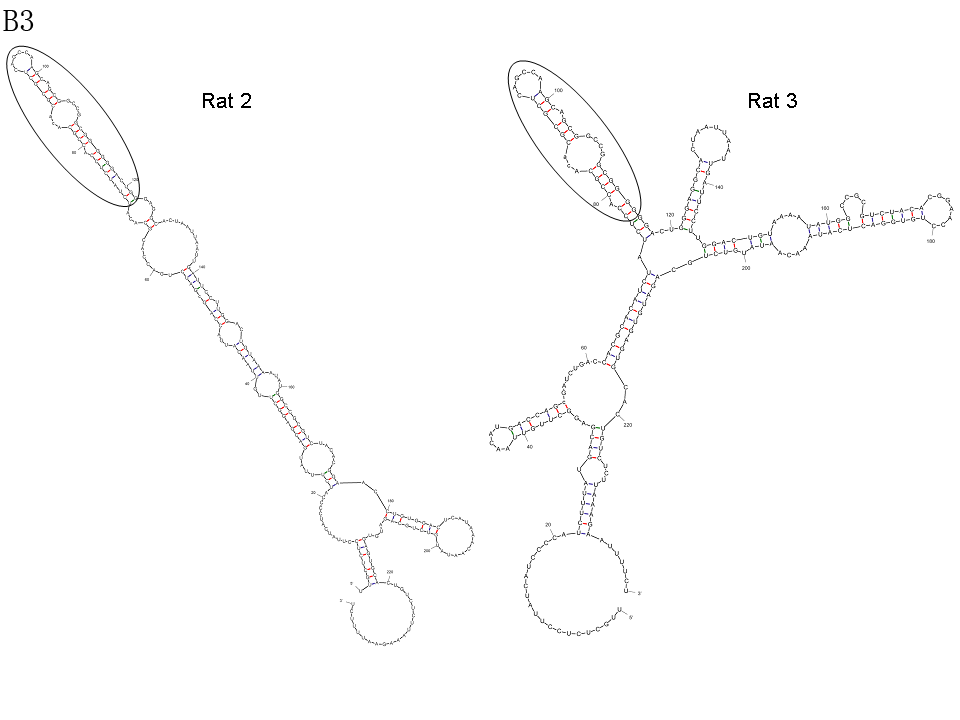


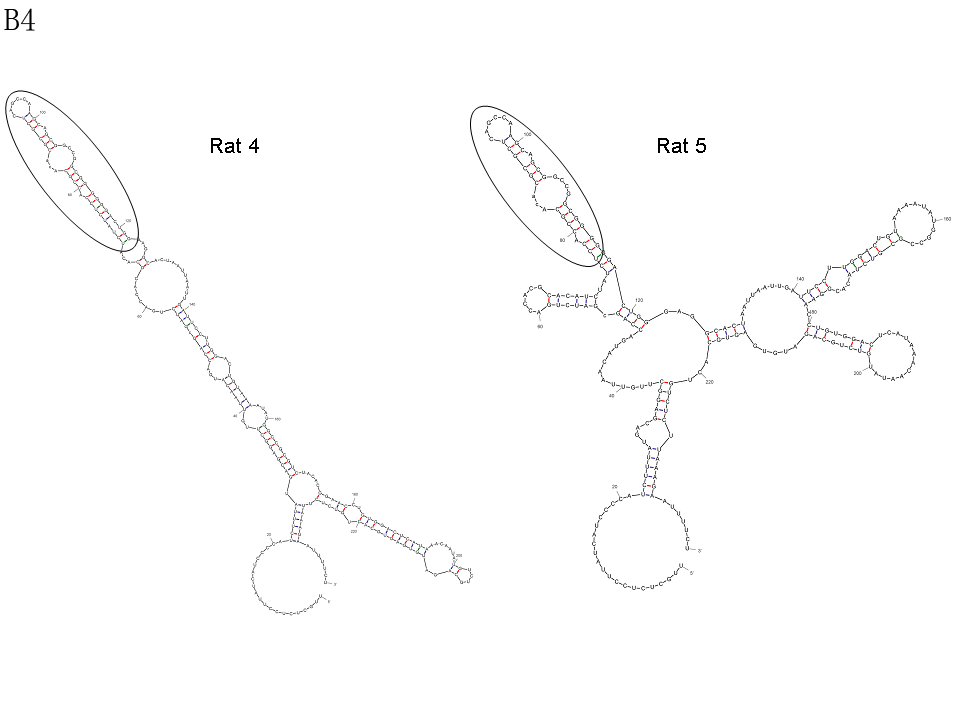


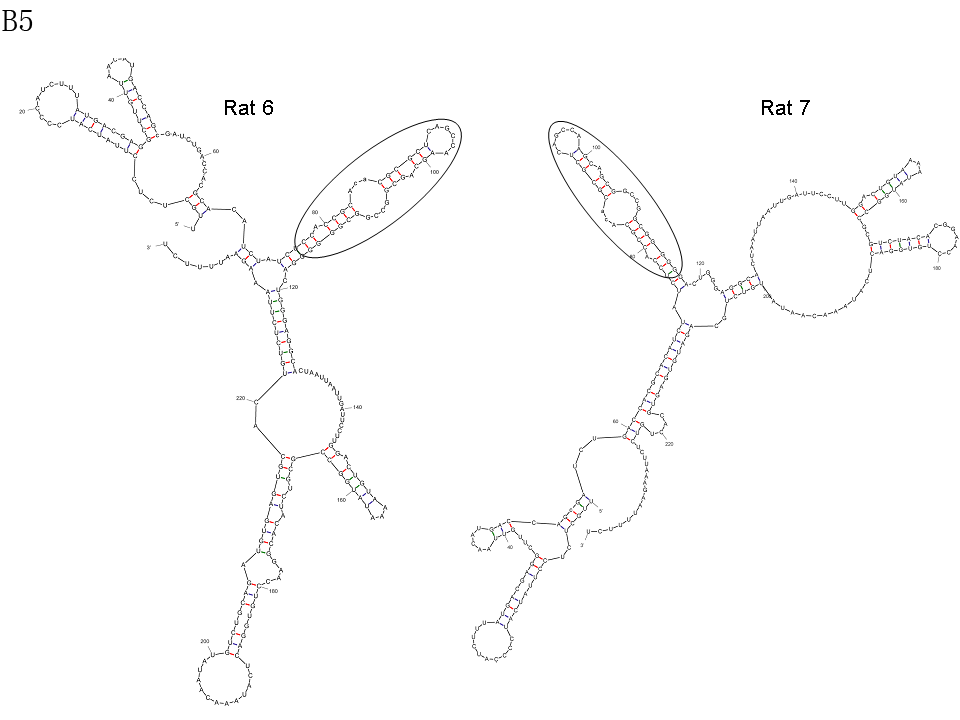


Figure S3. Predicted structures of domain B of exon6. (A) The structure in human and rat predicted by PMmulti and used by Infernal. (B) The structures predicted by Mfold, 3 in human and 7 in rat.
