# Supplementary material for: The sequence, structure and evolutionary features of HOTAIR in mammals
Source: BMC Evol Biol. 2011 Apr 16;11:102. doi: 10.1186/1471-2148-11-102 (PMC3103462; doi:10.1186/1471-2148-11-102)
Supplement: Additional file 3 — This file contains predicted structures of full HOTAIR in human and rat. [file 1471-2148-11-102-S3.PDF]

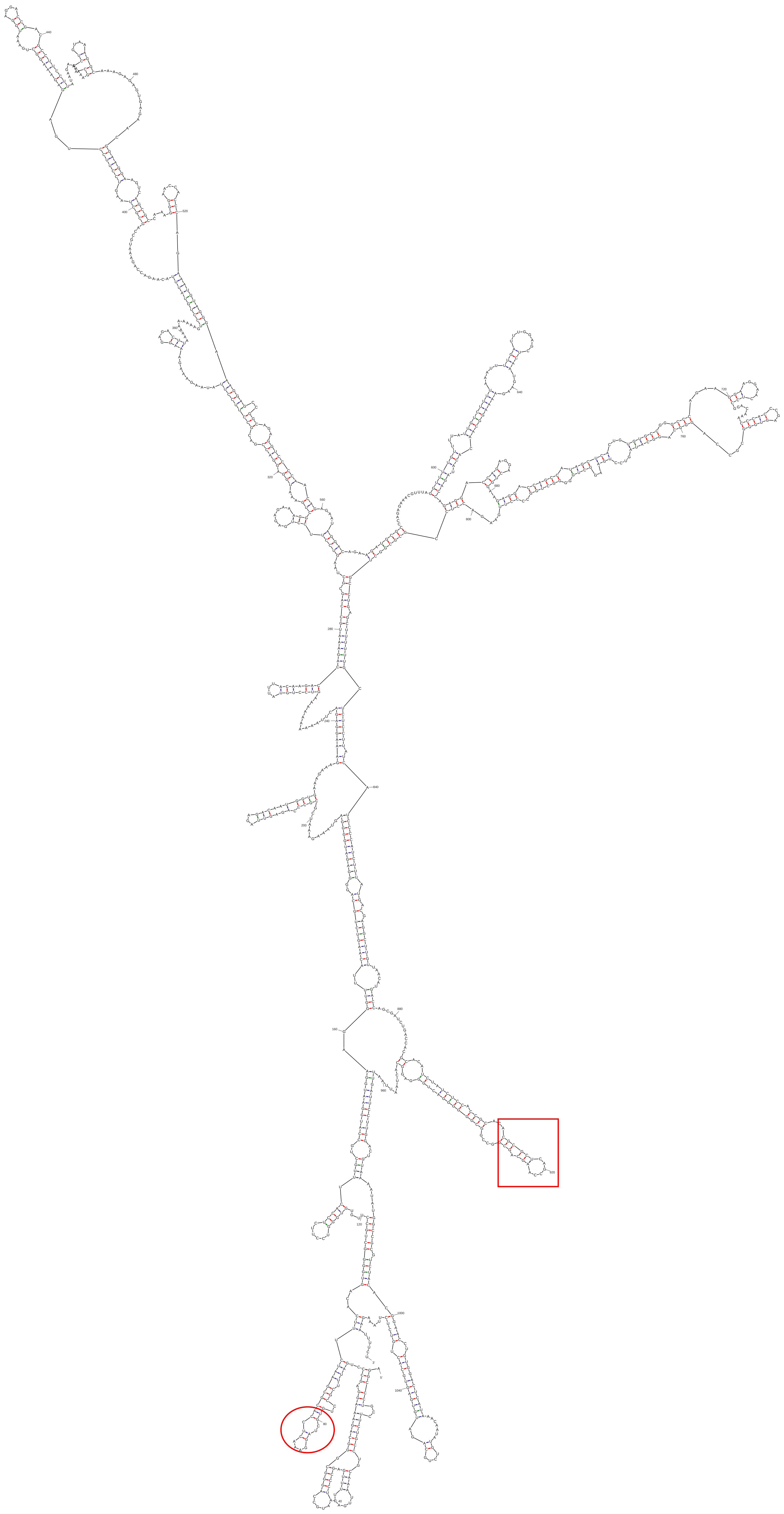

dG = -294.43 [Initially -336.90] rat-full

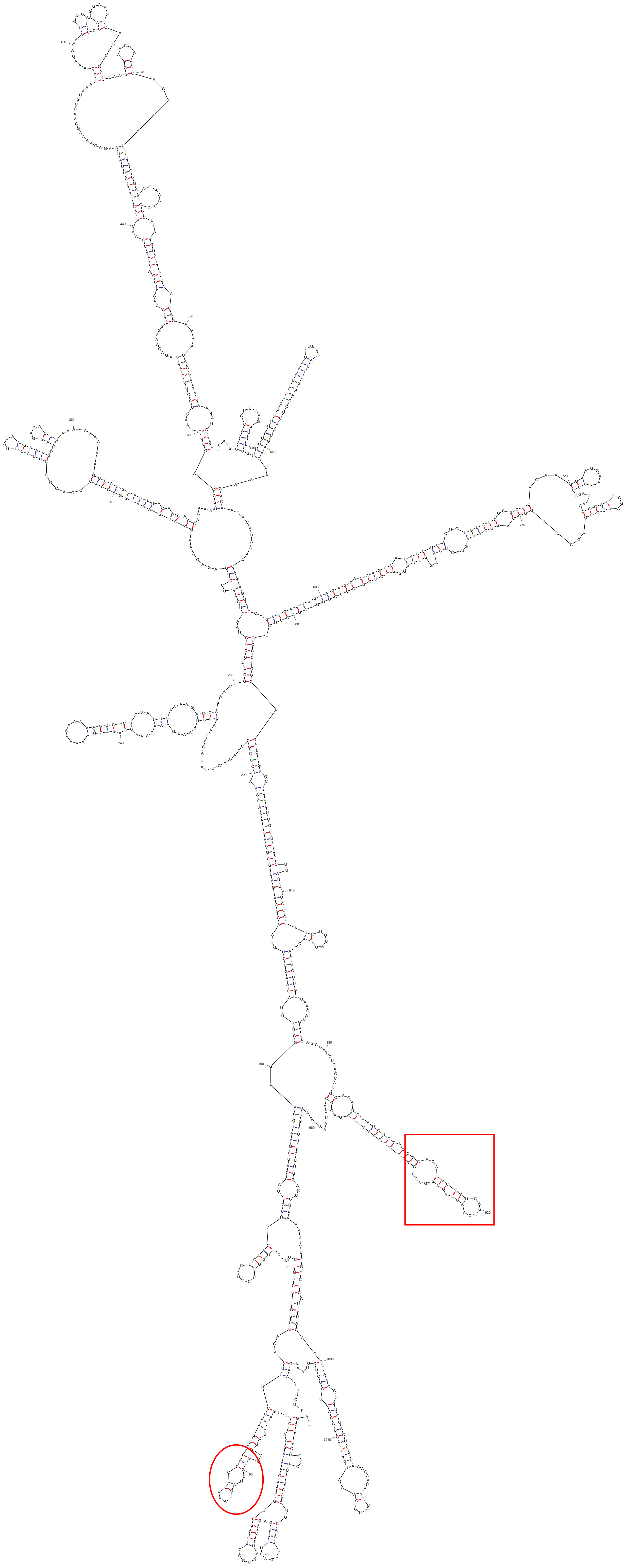

dG = -290.73 [Initially -333.50] rat-full

Output of sir_graph (©)
mfold_util 4.6

Created Thu Mar 10 19:48:38 2011

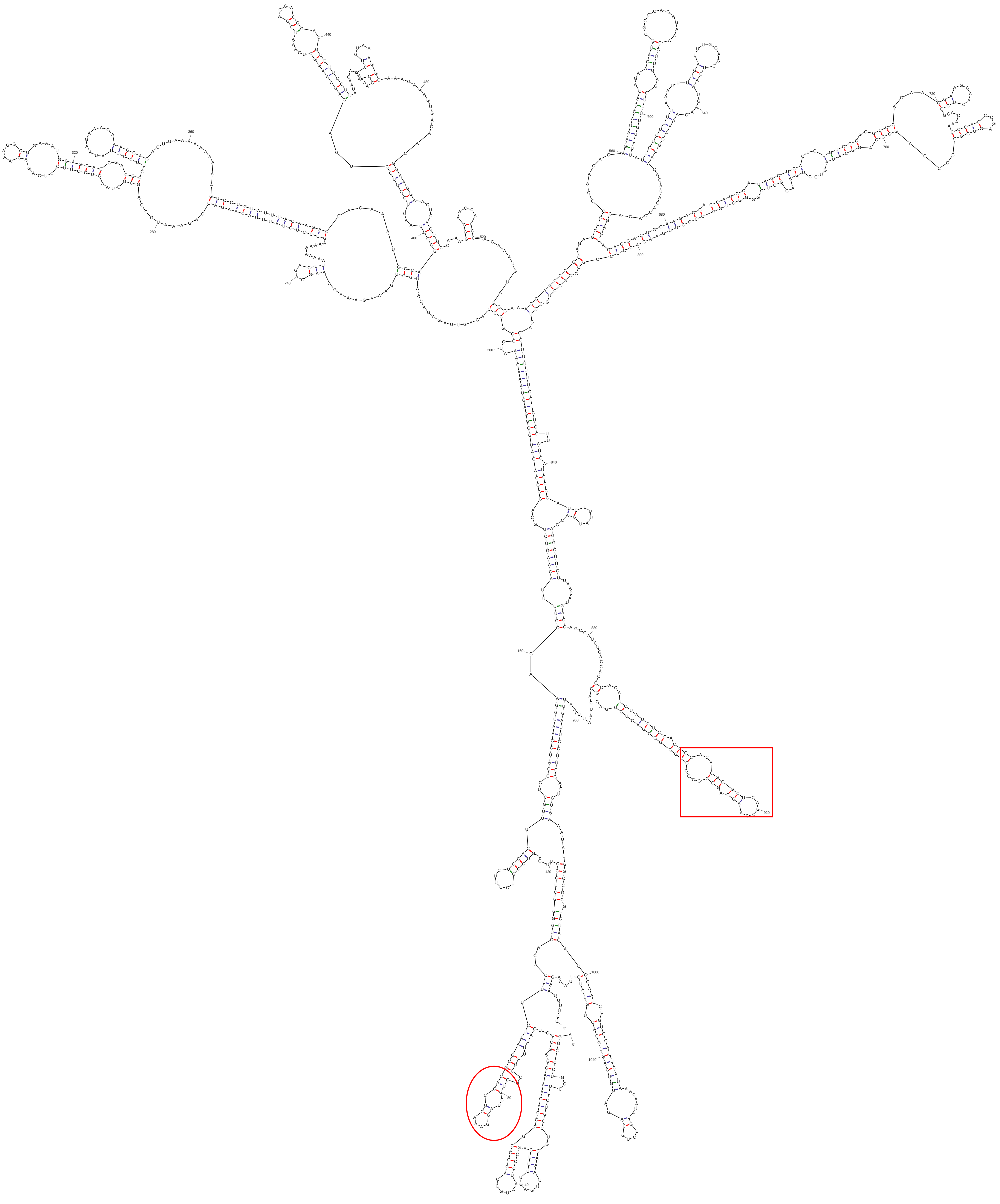

dG = -288.91 [Initially -333.40] rat-full

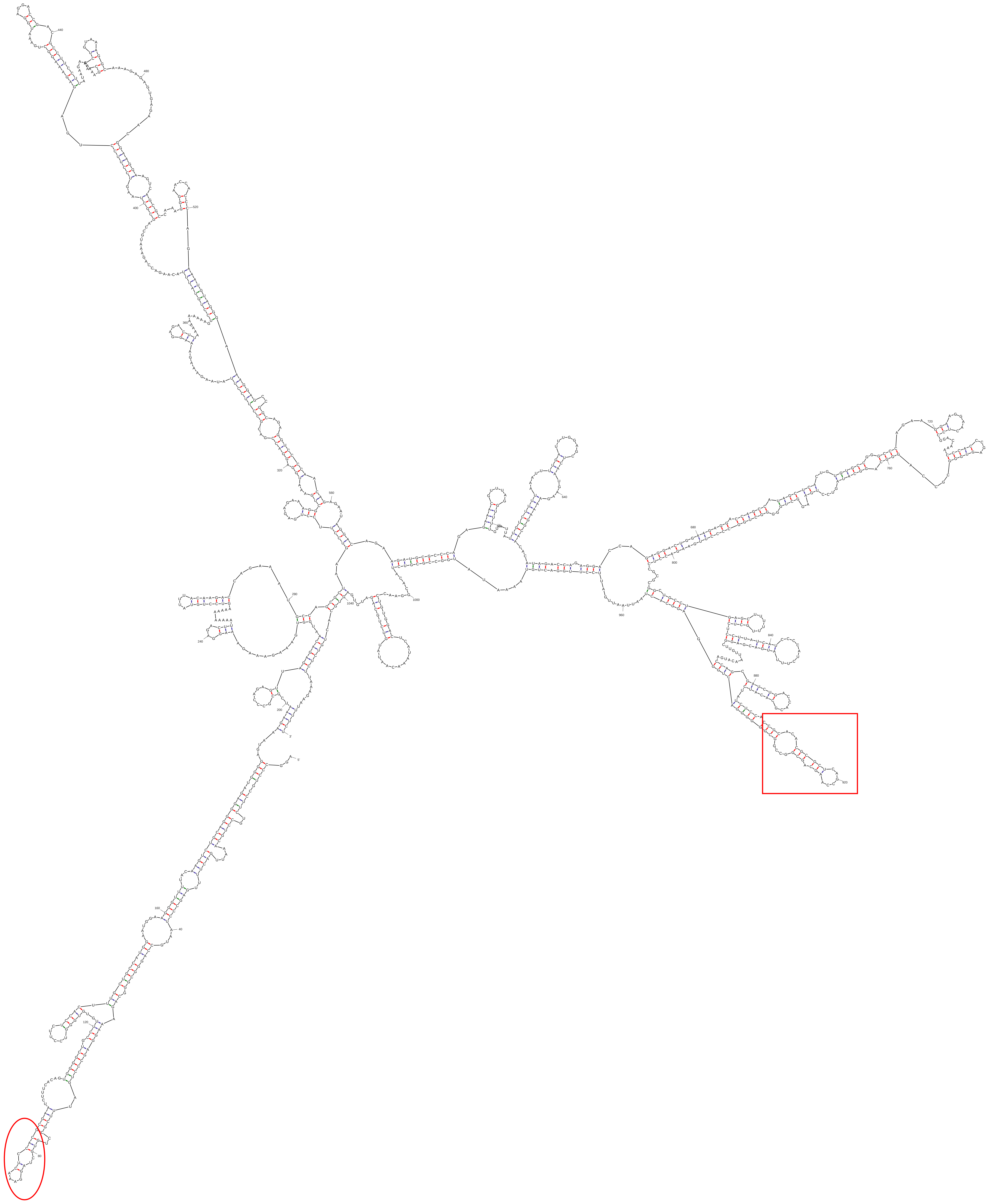

dG = -294.52 [Initially -332.40] rat-full

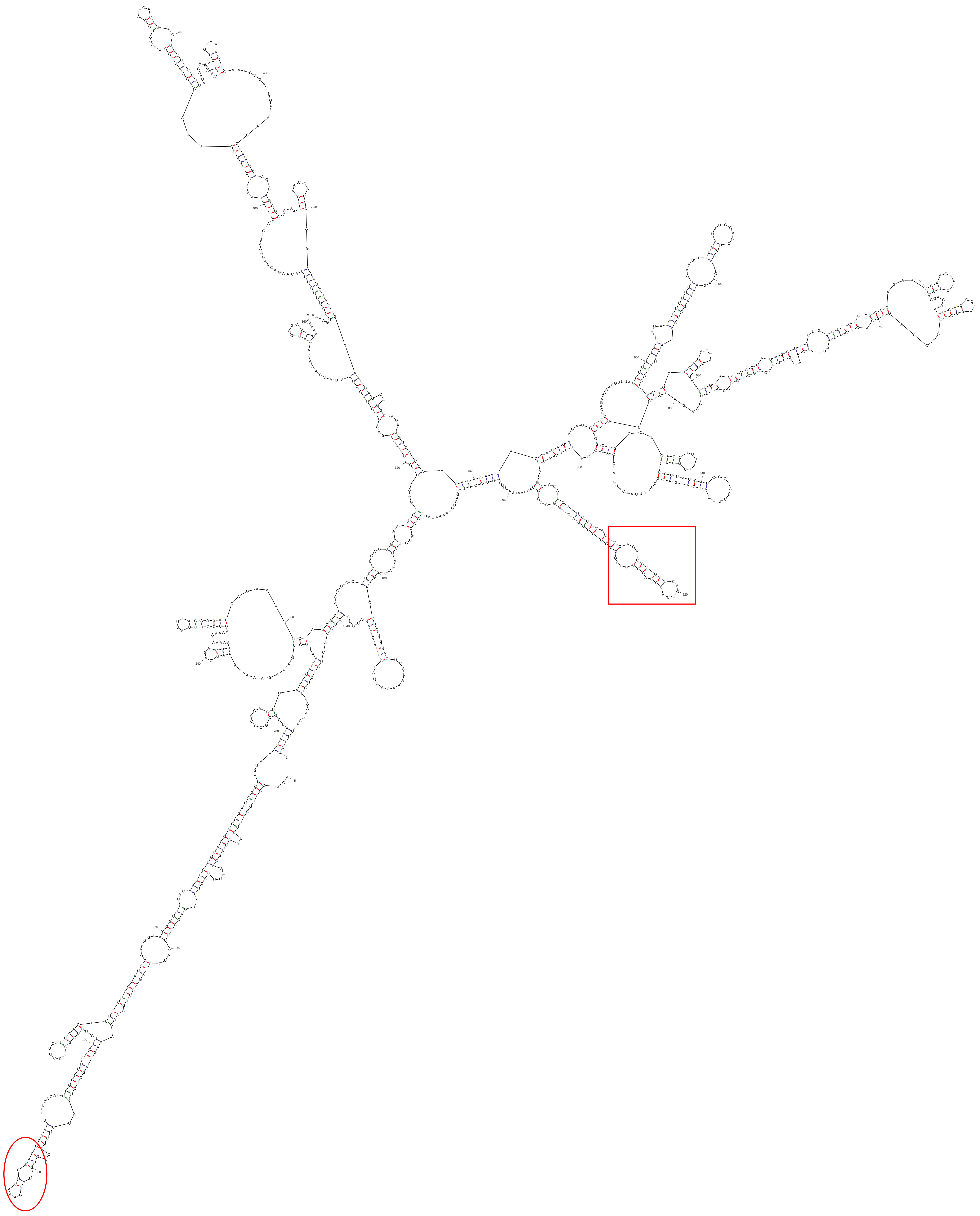

dG = -284.29 [Initially -332.10] rat-full

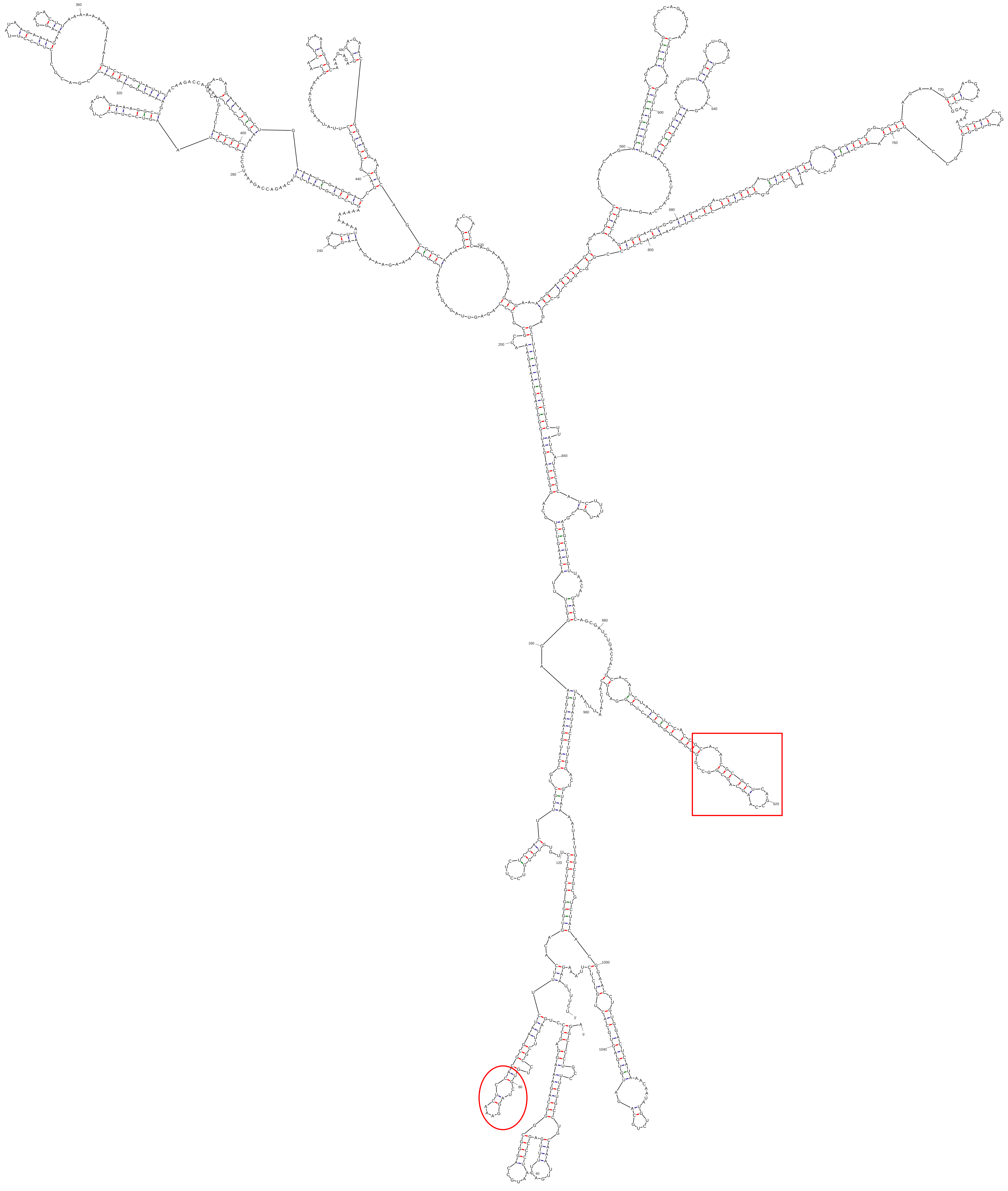

dG = -286.44 [Initially -332.00] rat-full

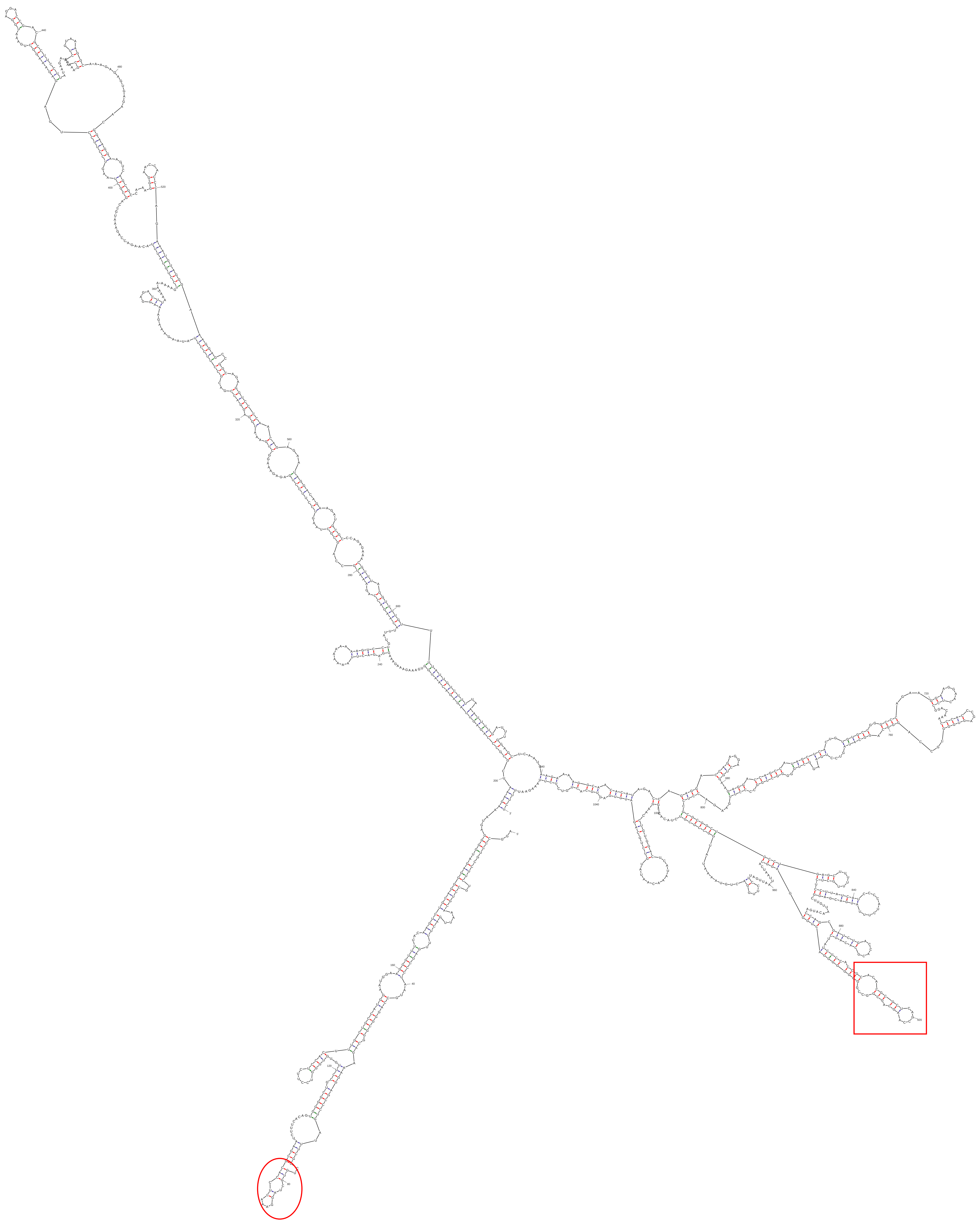

dG = -296.79 [Initially -331.90] rat-full

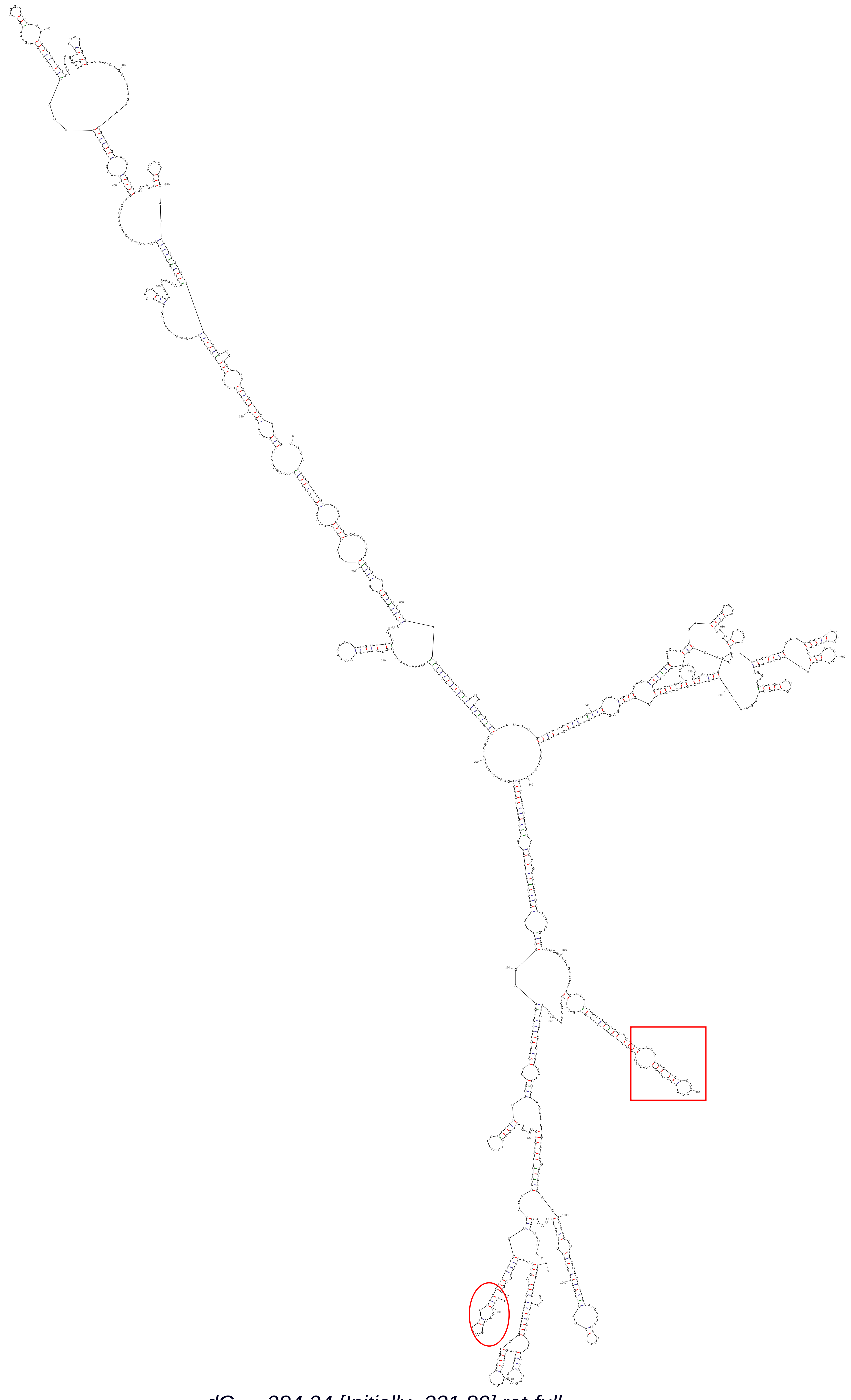

dG = -284.24 [Initially -331.80] rat-full

Output of sir_graph (©)
mfold_util 4.6

Created Thu Mar 10 19:48:40 2011

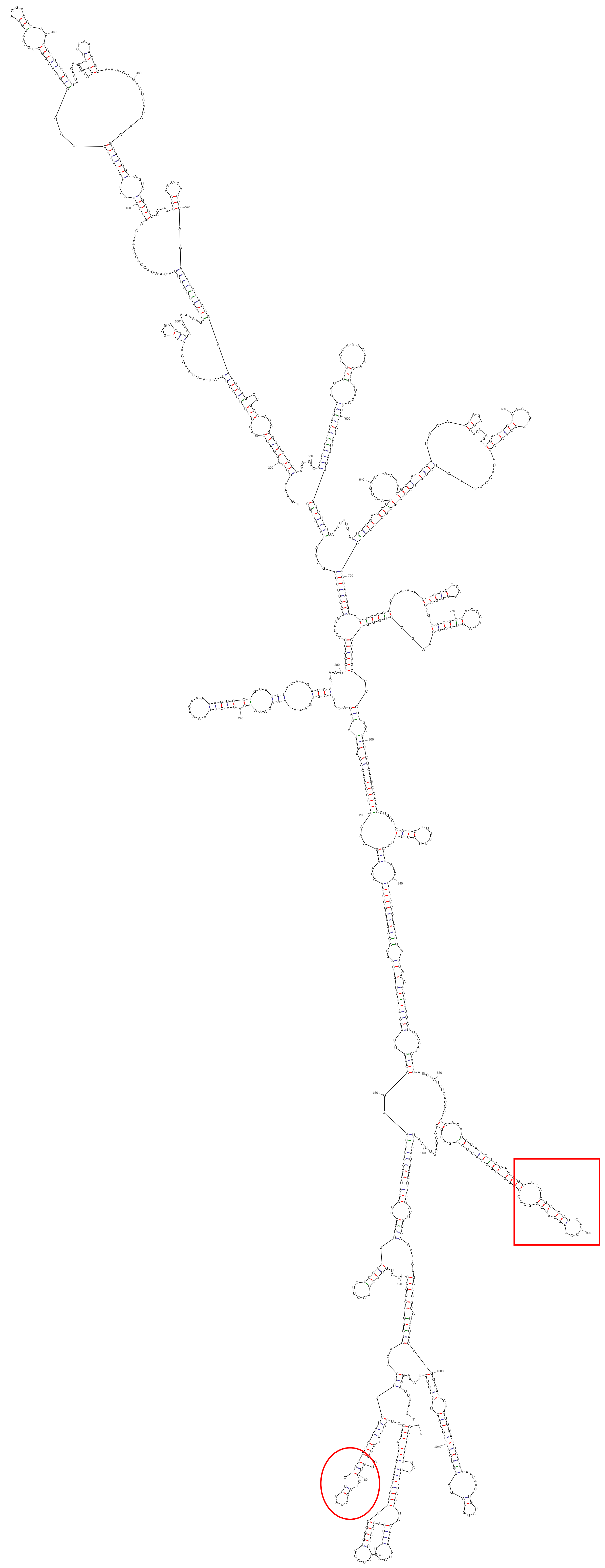

dG = -282.97 [Initially -331.70] rat-full

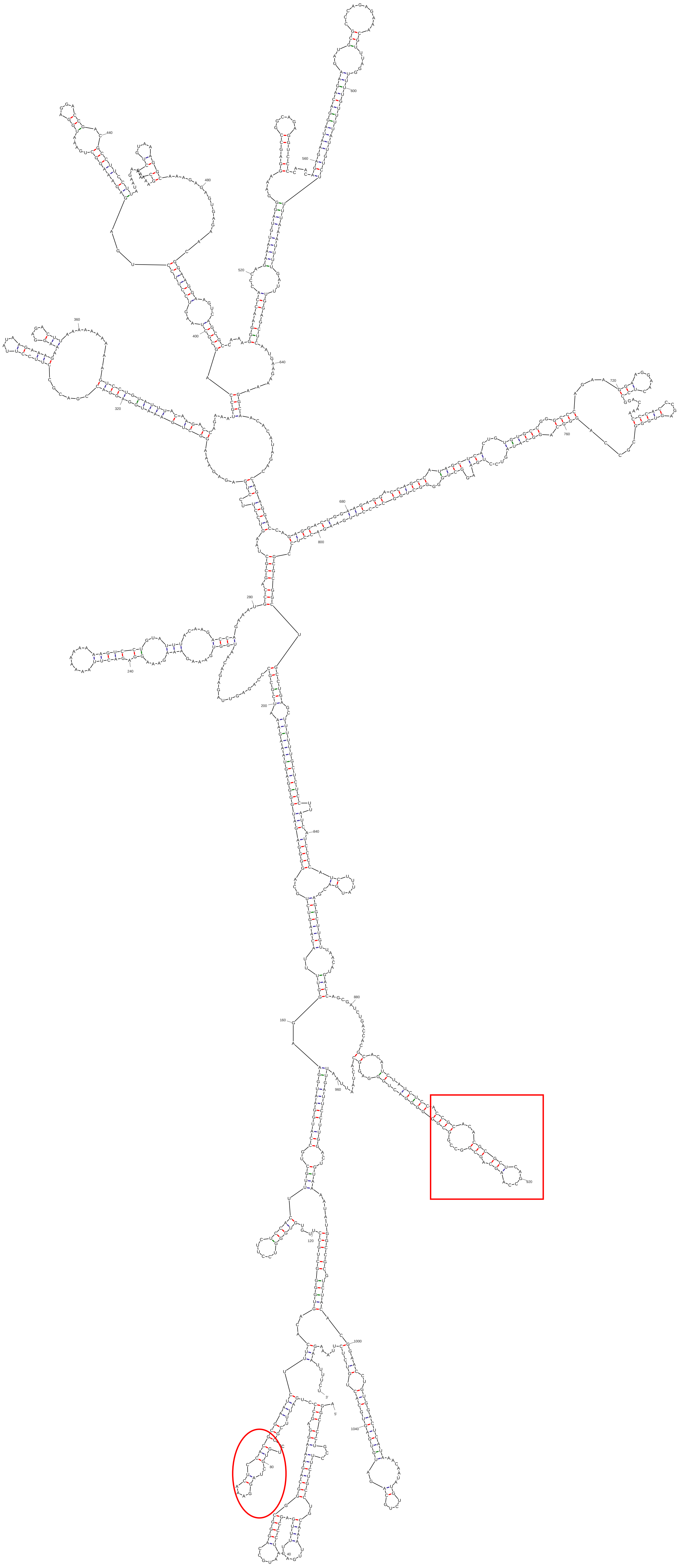

dG = -286.29 [Initially -331.60] rat-full

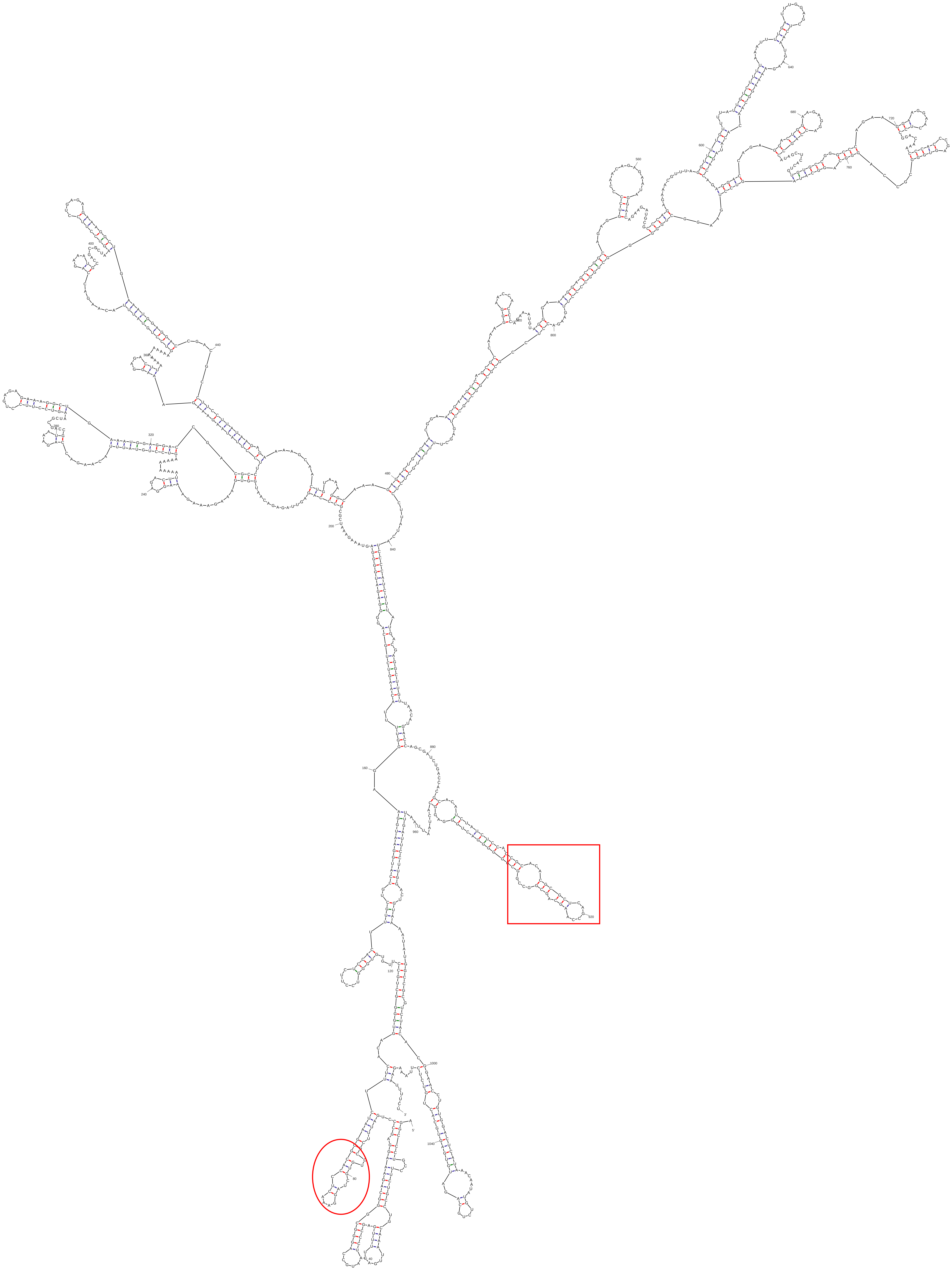

dG = -280.83 [Initially -331.40] rat-full

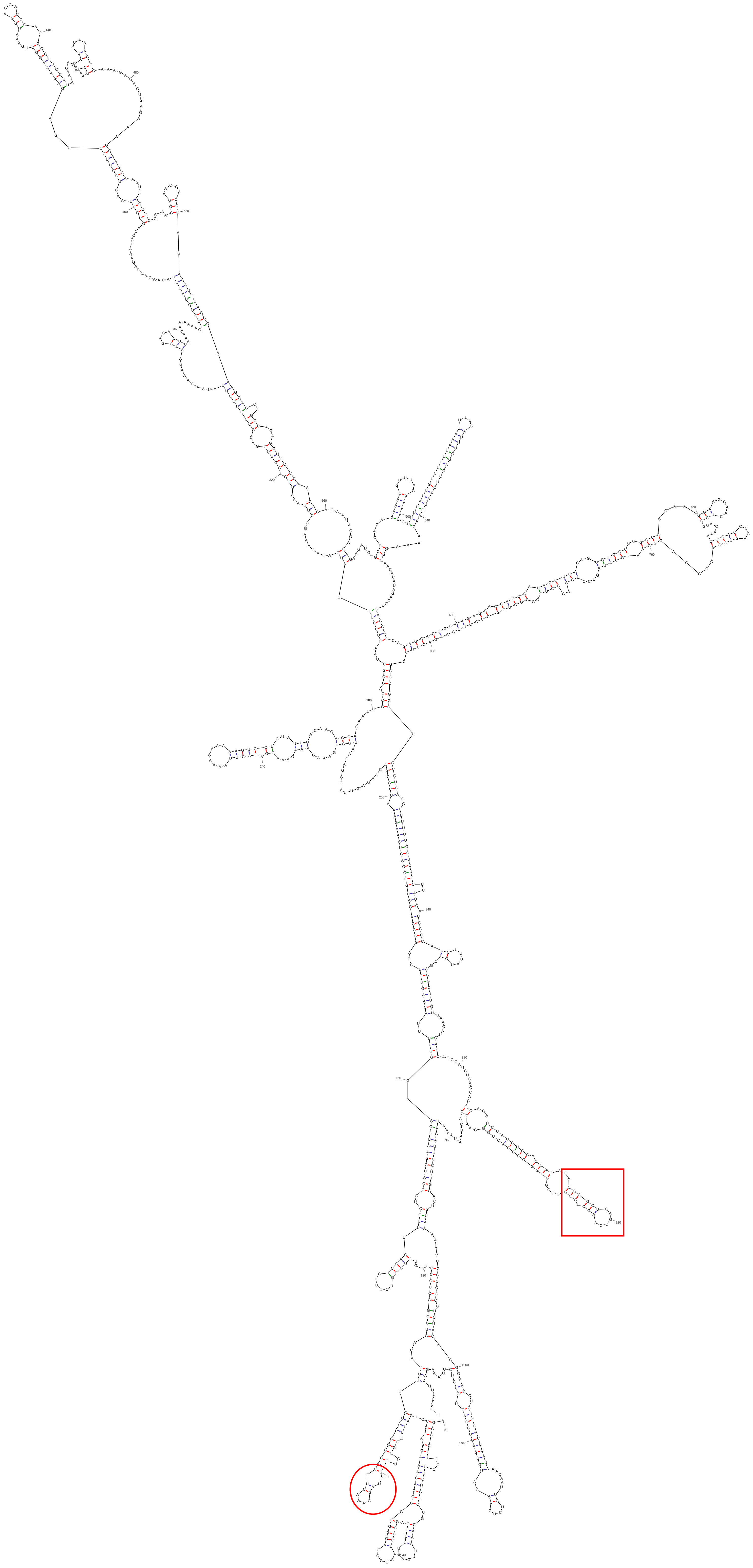

dG = -293.29 [Initially -336.00] rat-full

Output of sir_graph (©)
mfold_util 4.6

Created Thu Mar 10 19:48:42 2011

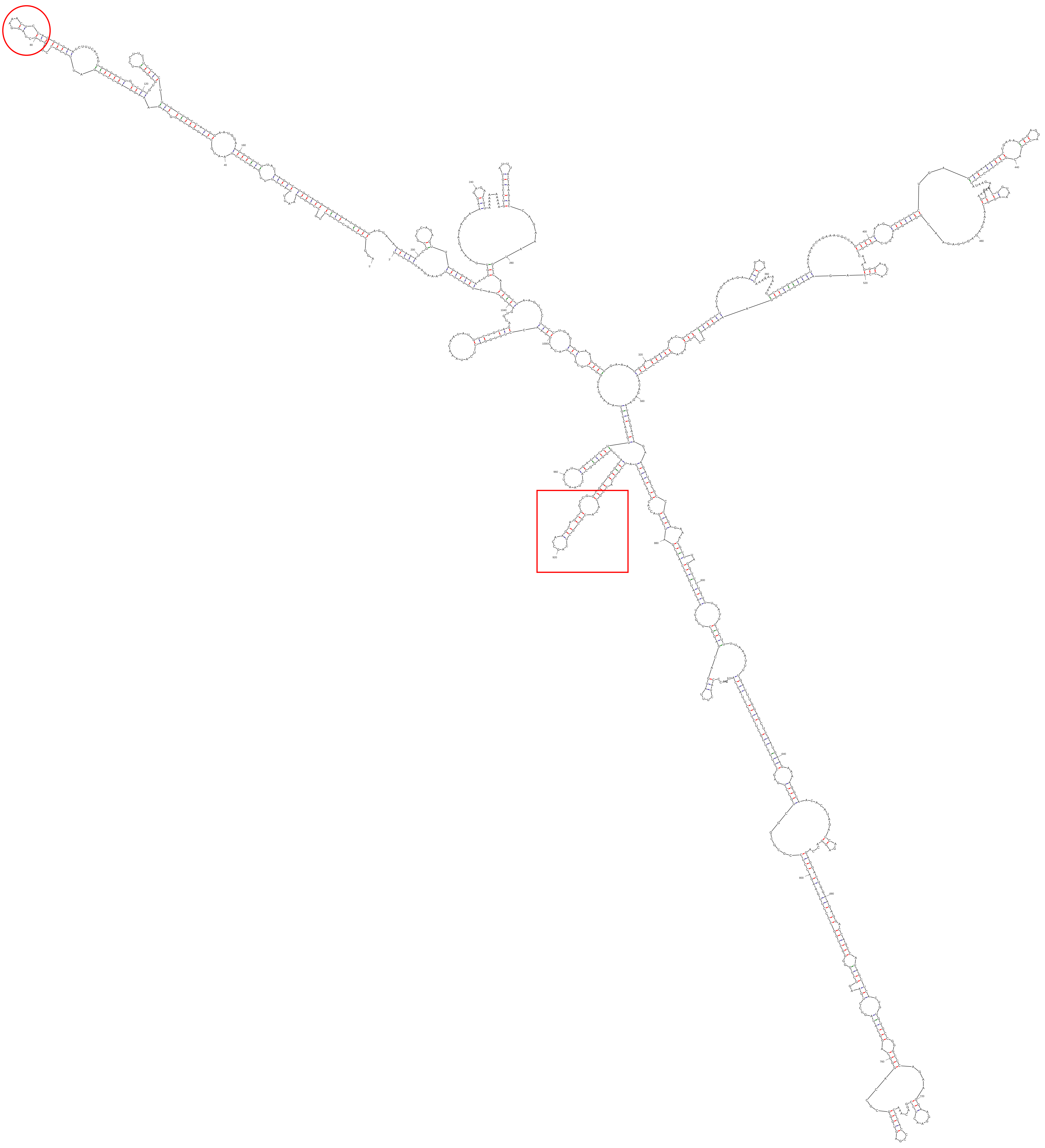

dG = -289.54 [Initially -330.90] rat-full

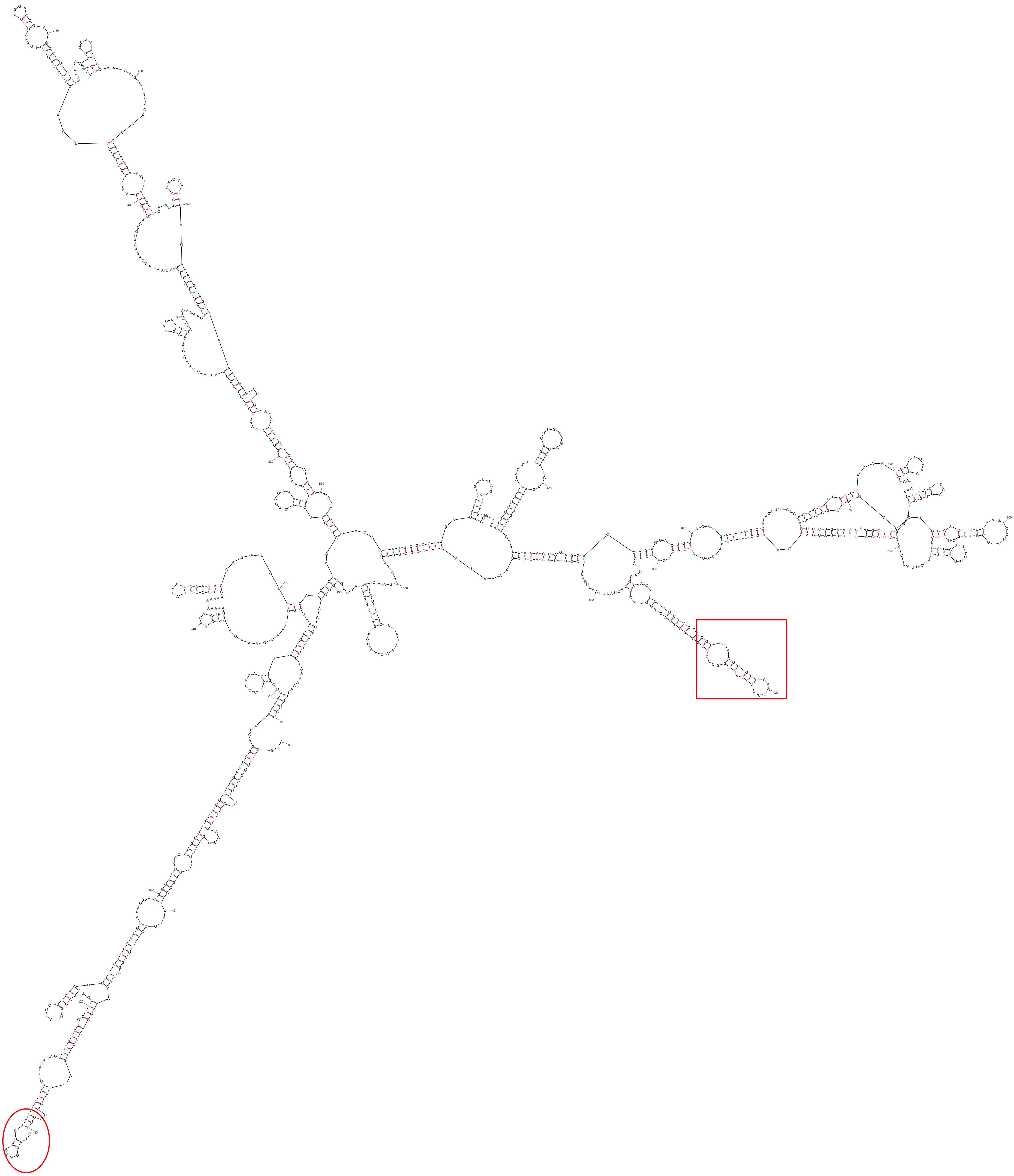

dG = -287.57 [Initially -330.60] rat-full

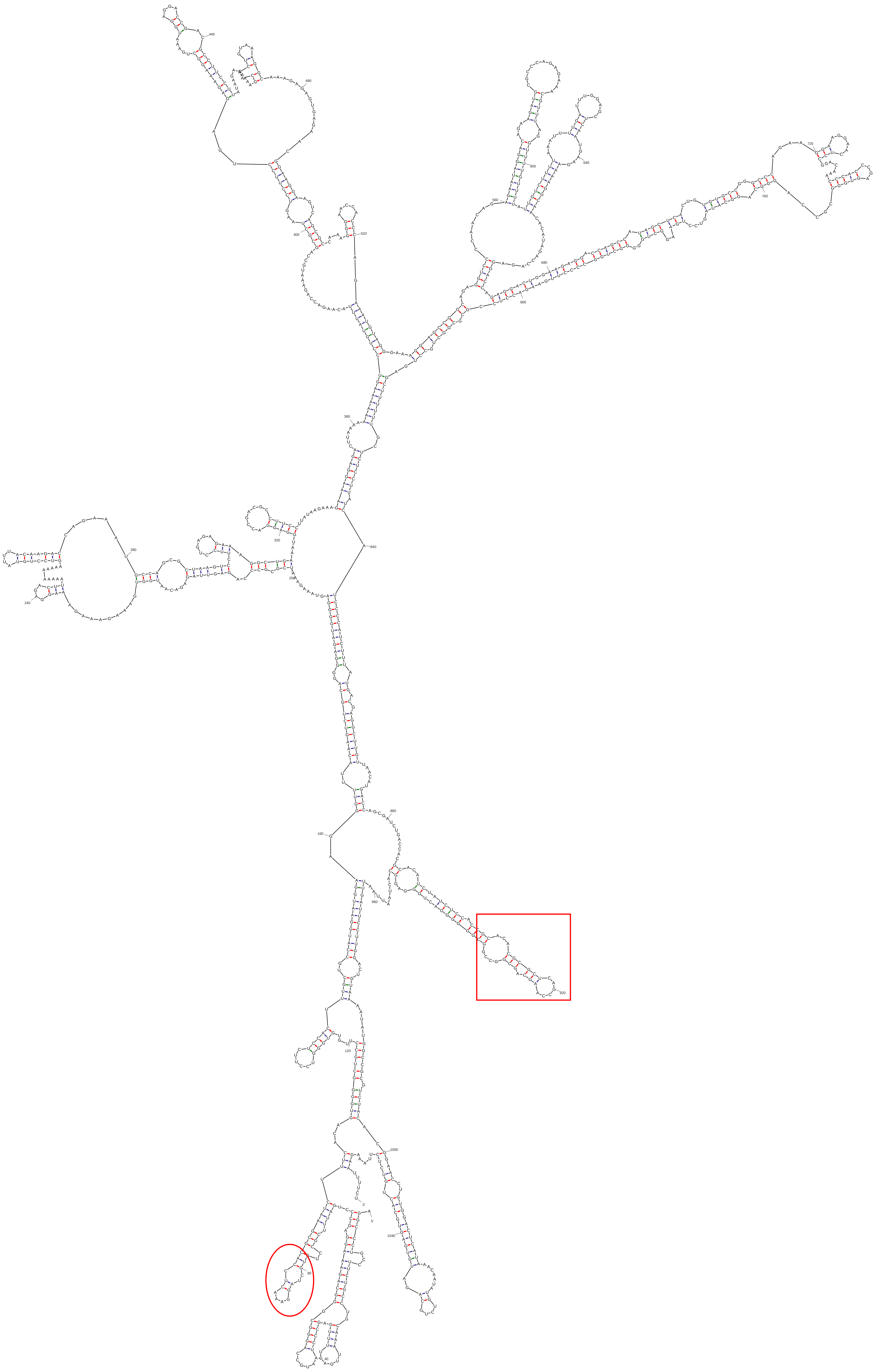

dG = -284.97 [Initially -330.00] rat-full

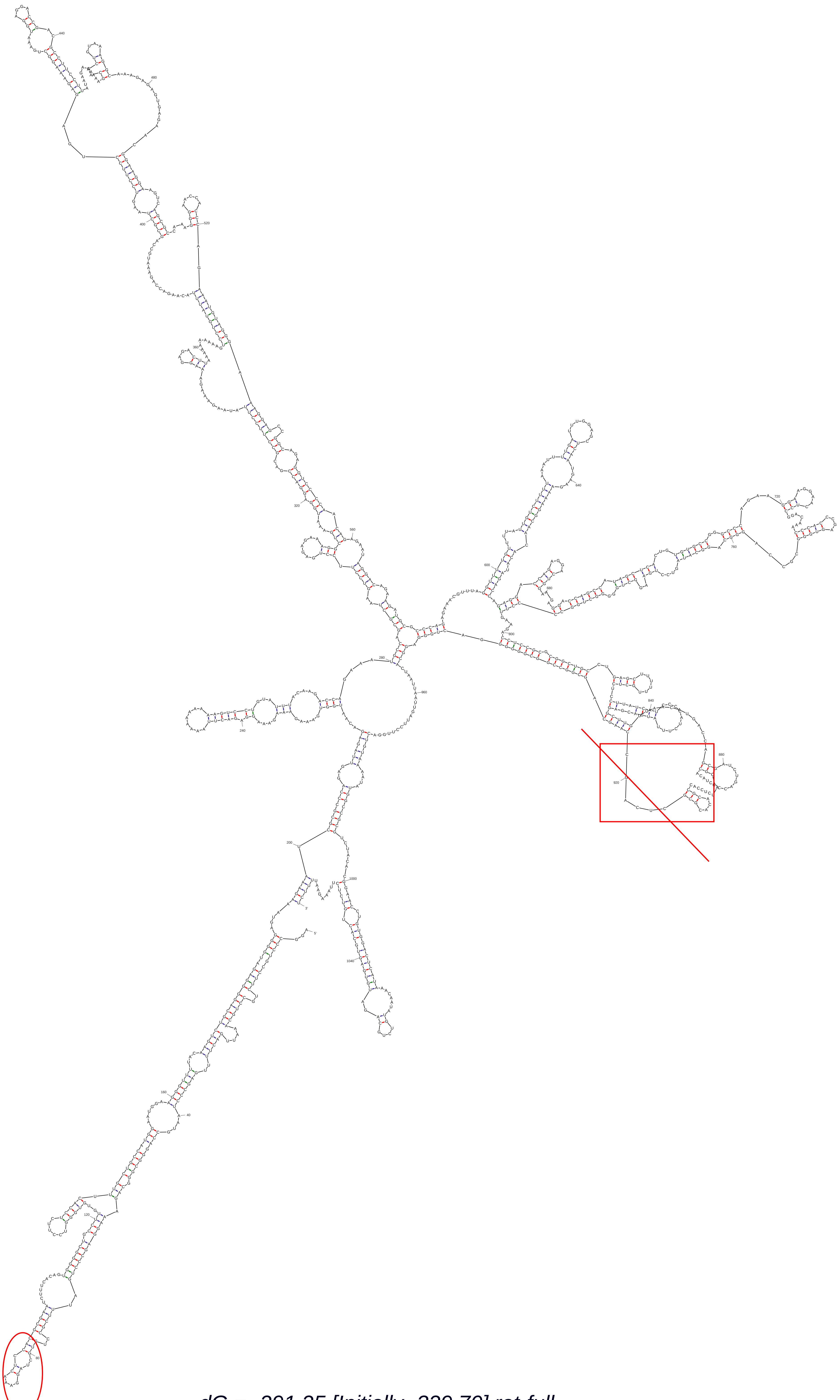

dG = -291.25 [Initially -329.70] rat-full

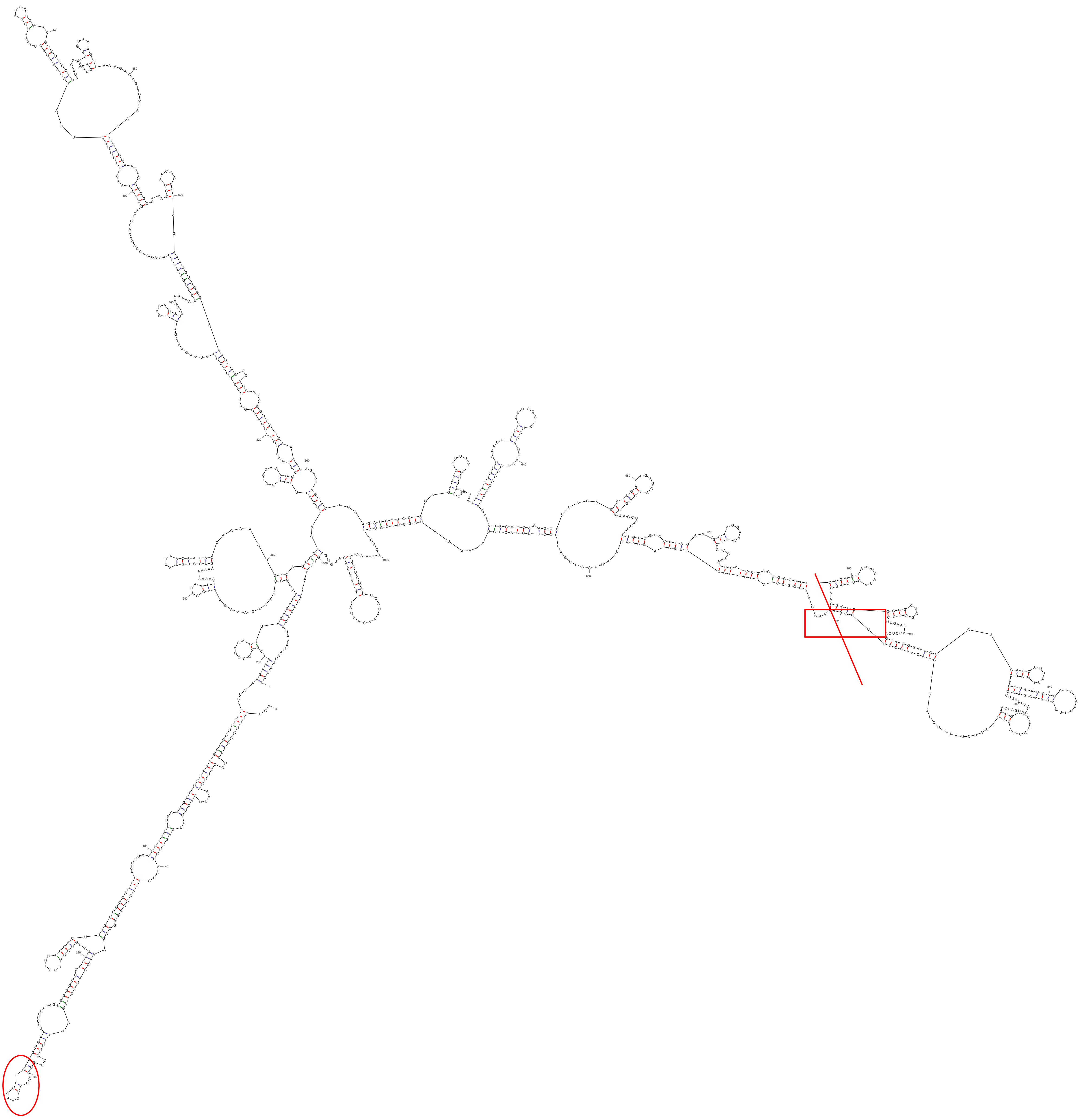

dG = -287.26 [Initially -329.70] rat-full

Output of sir_graph (©)
mfold_util 4.6

Created Thu Mar 10 19:48:43 2011

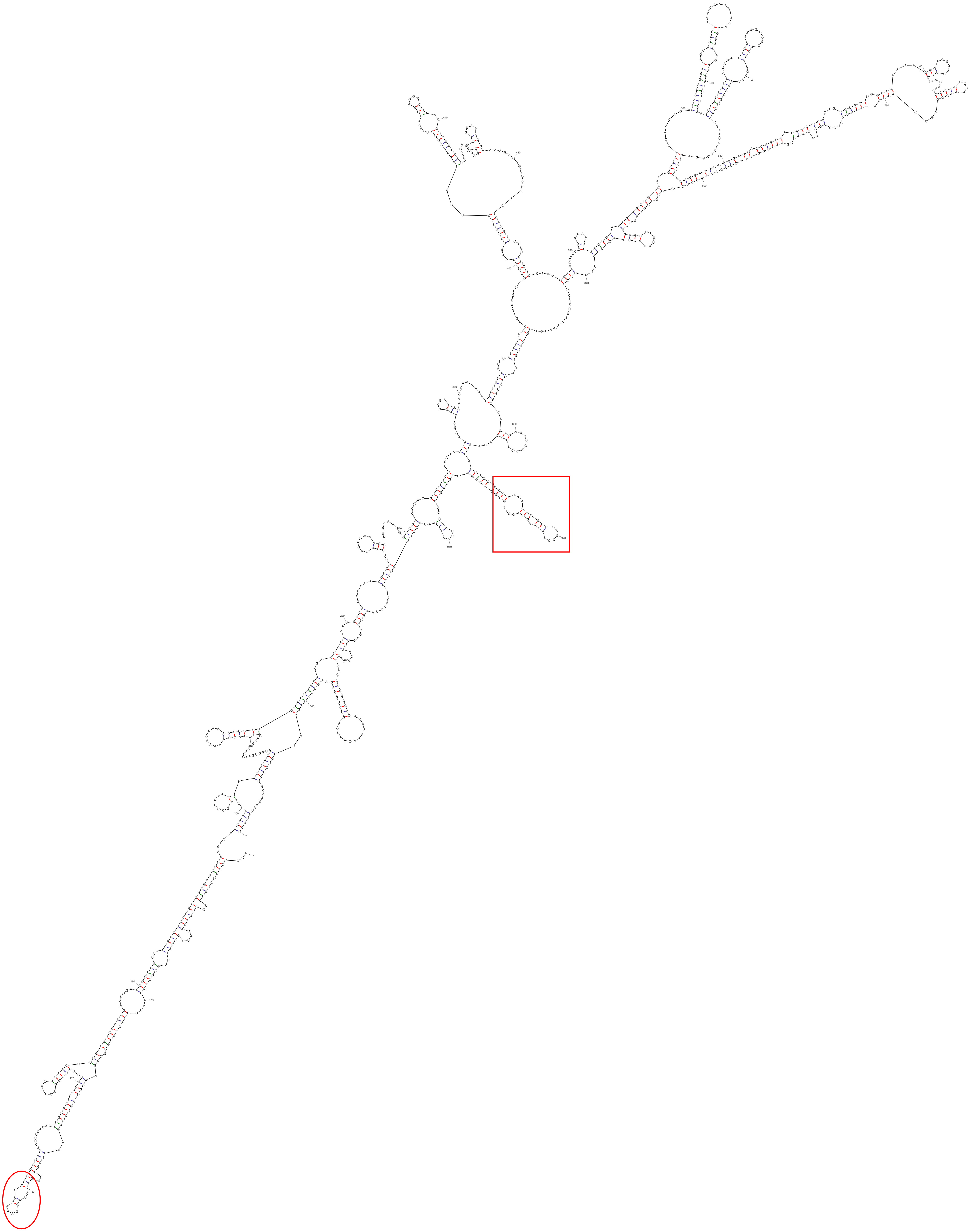

dG = -286.76 [Initially -329.50] rat-full

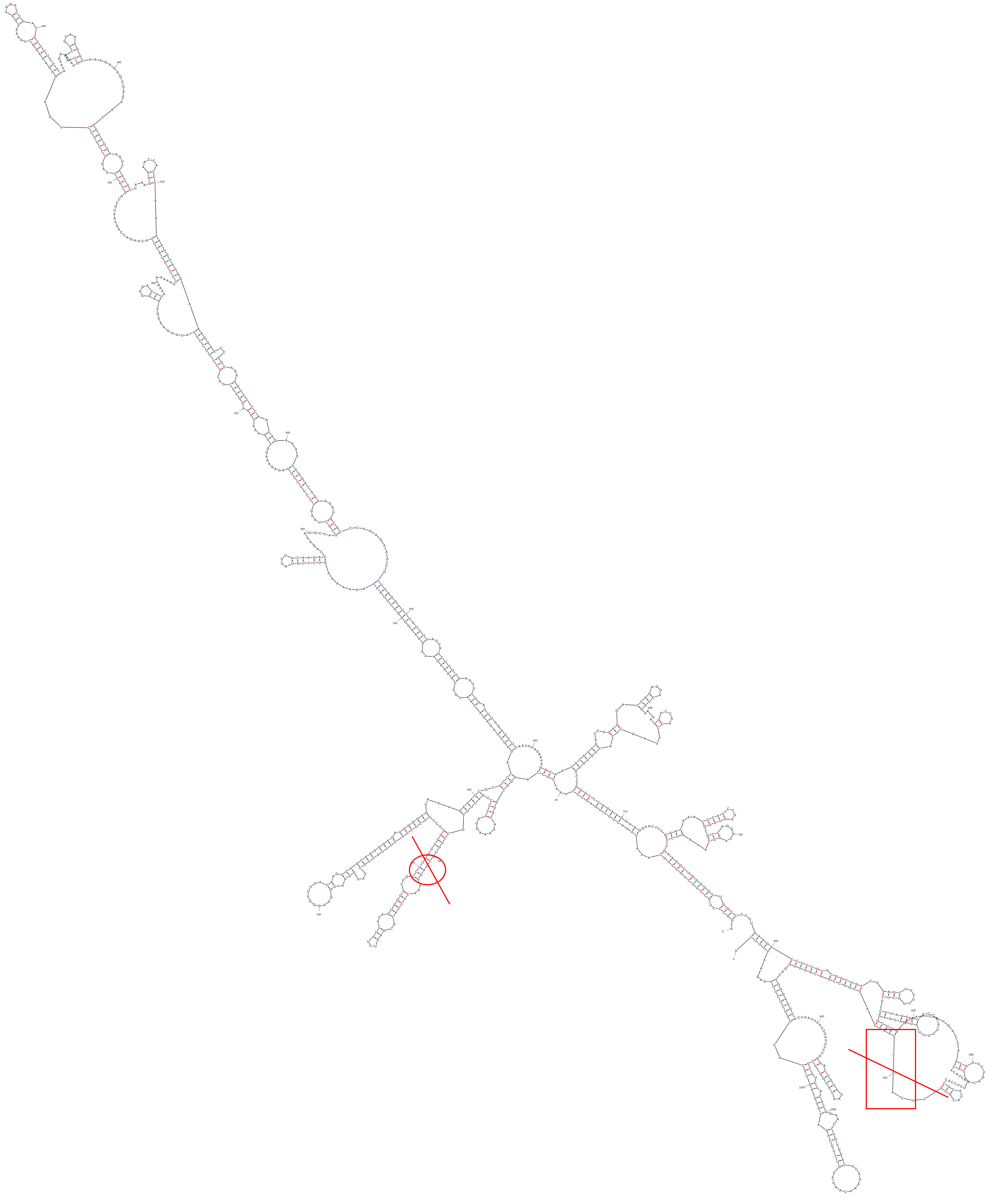

dG = -277.94 [Initially -326.90] rat-full

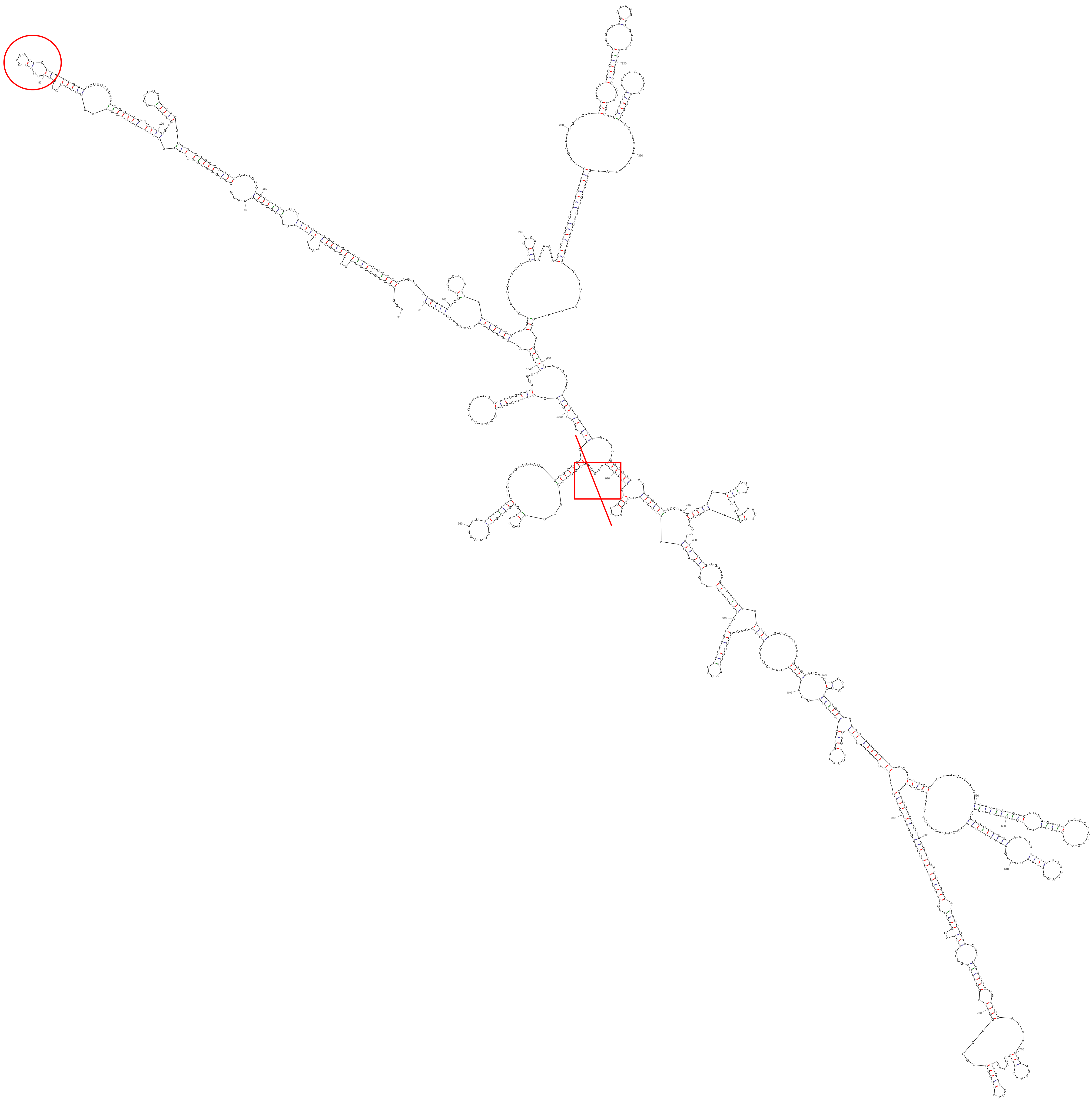

dG = -284.80 [Initially -326.70] rat-full

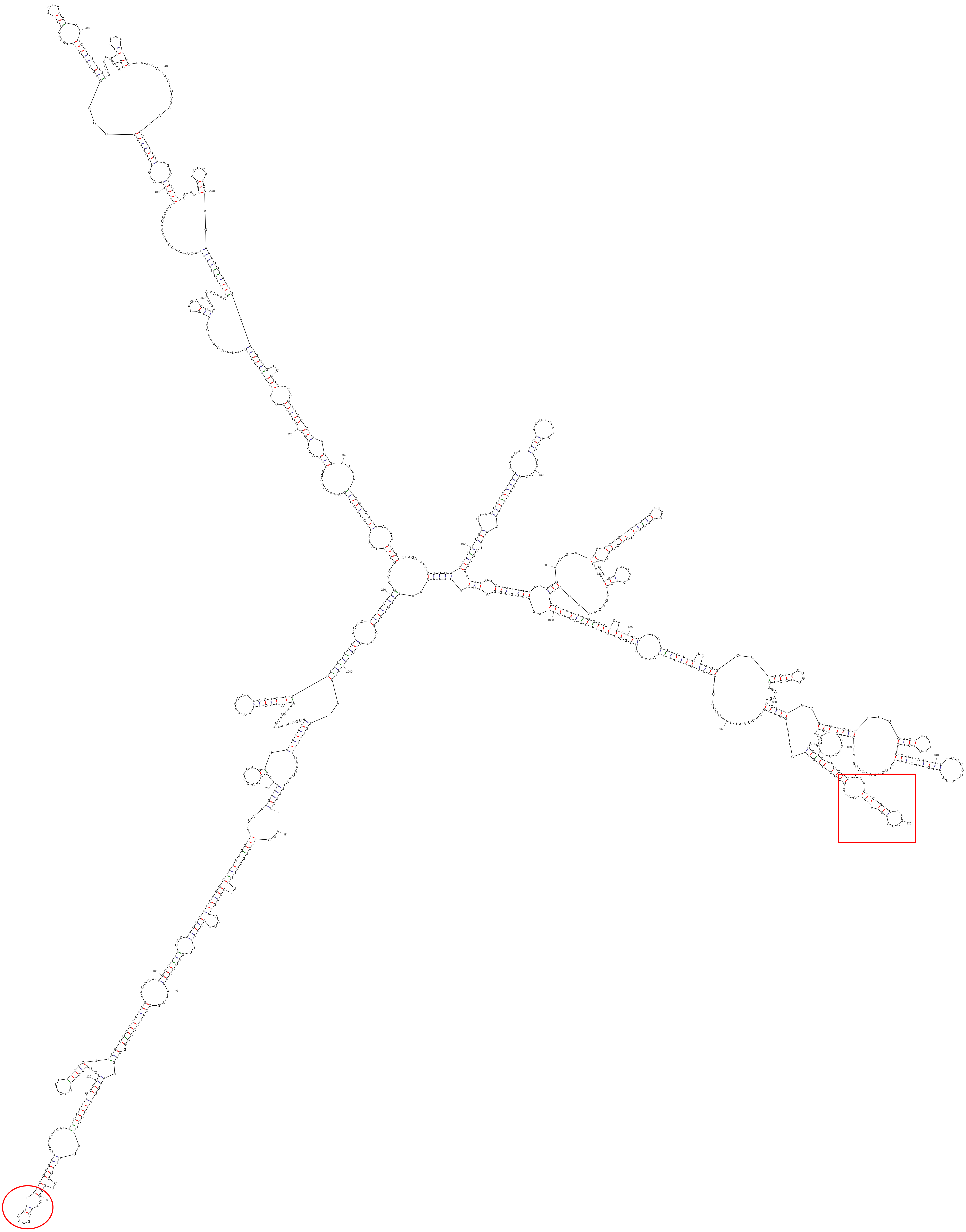

dG = -283.70 [Initially -326.30] rat-full

Output of sir_graph (©)
mfold_util 4.6

Created Thu Mar 10 19:48:45 2011

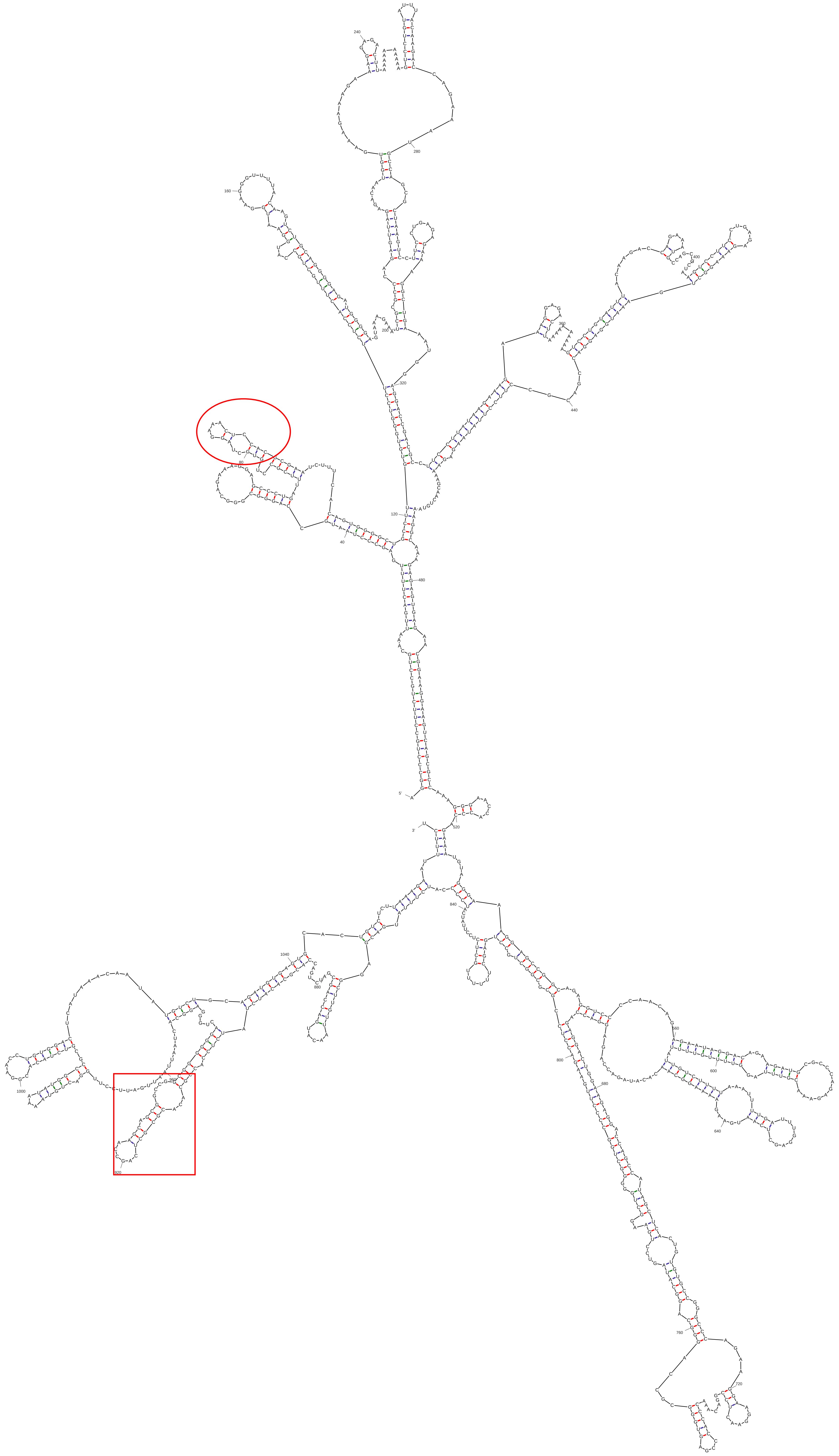

dG = -281.75 [Initially -326.20] rat-full

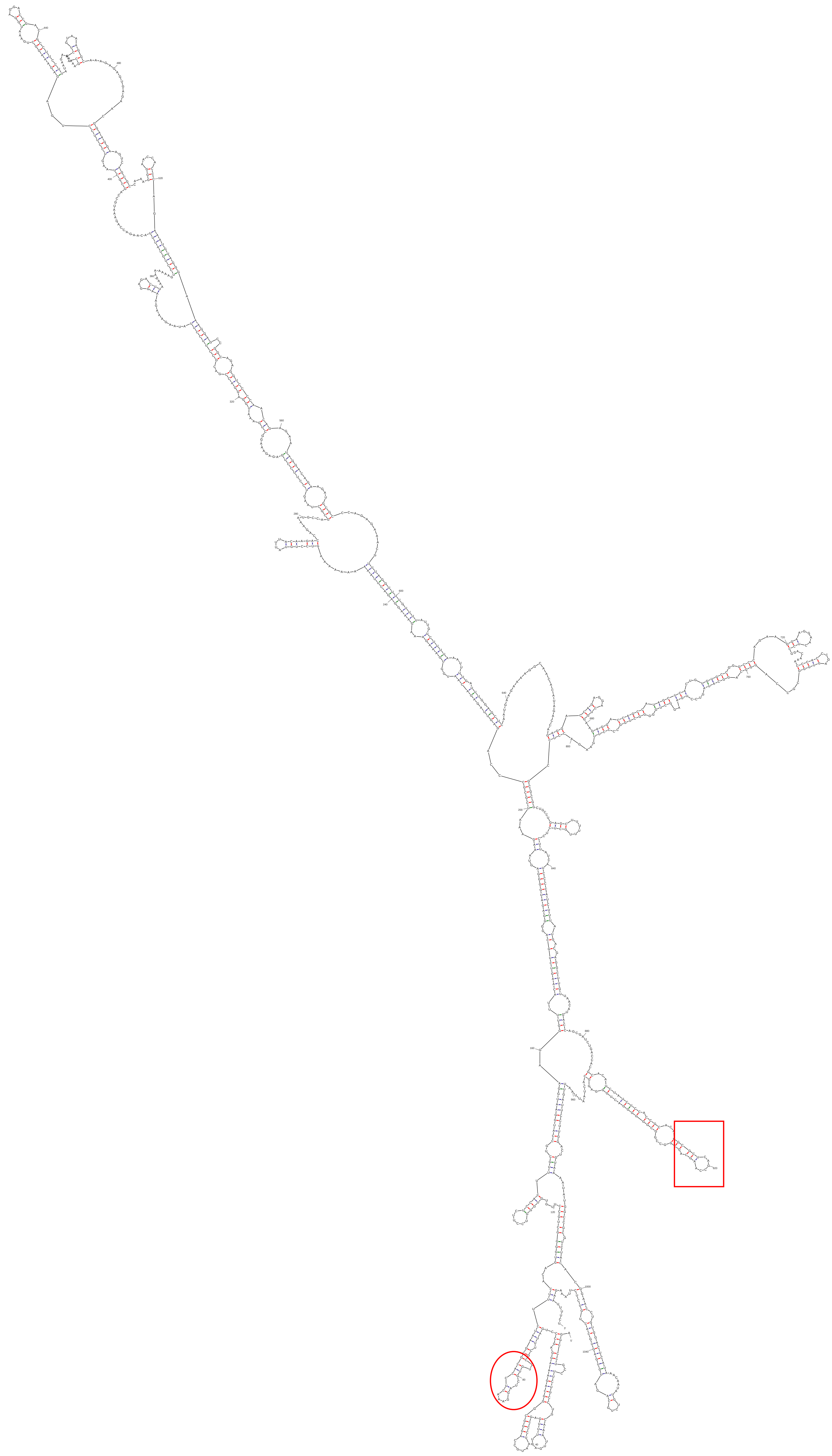

dG = -290.42 [Initially -335.80] rat-full

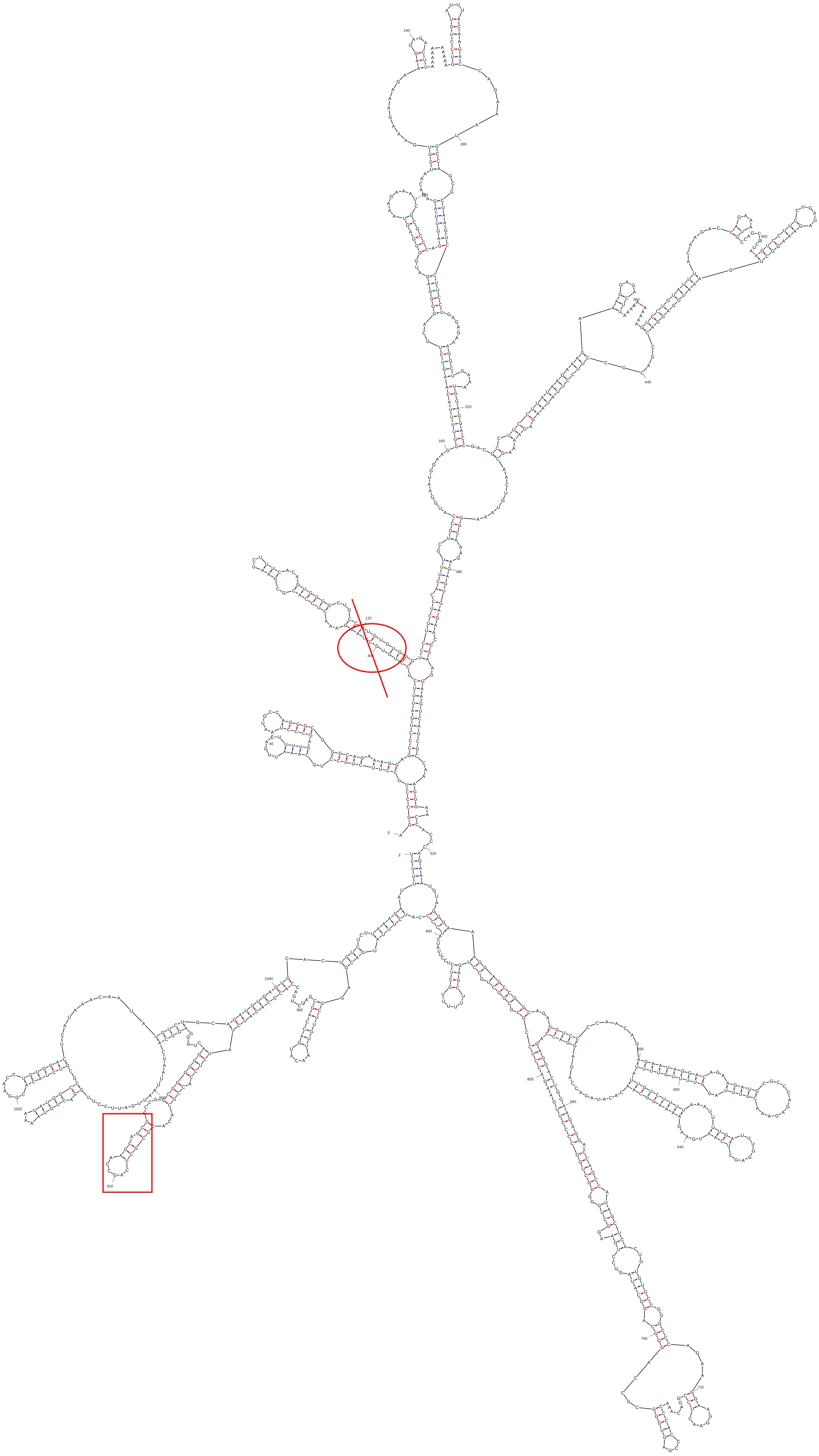

dG = -279.88 [Initially -326.00] rat-full

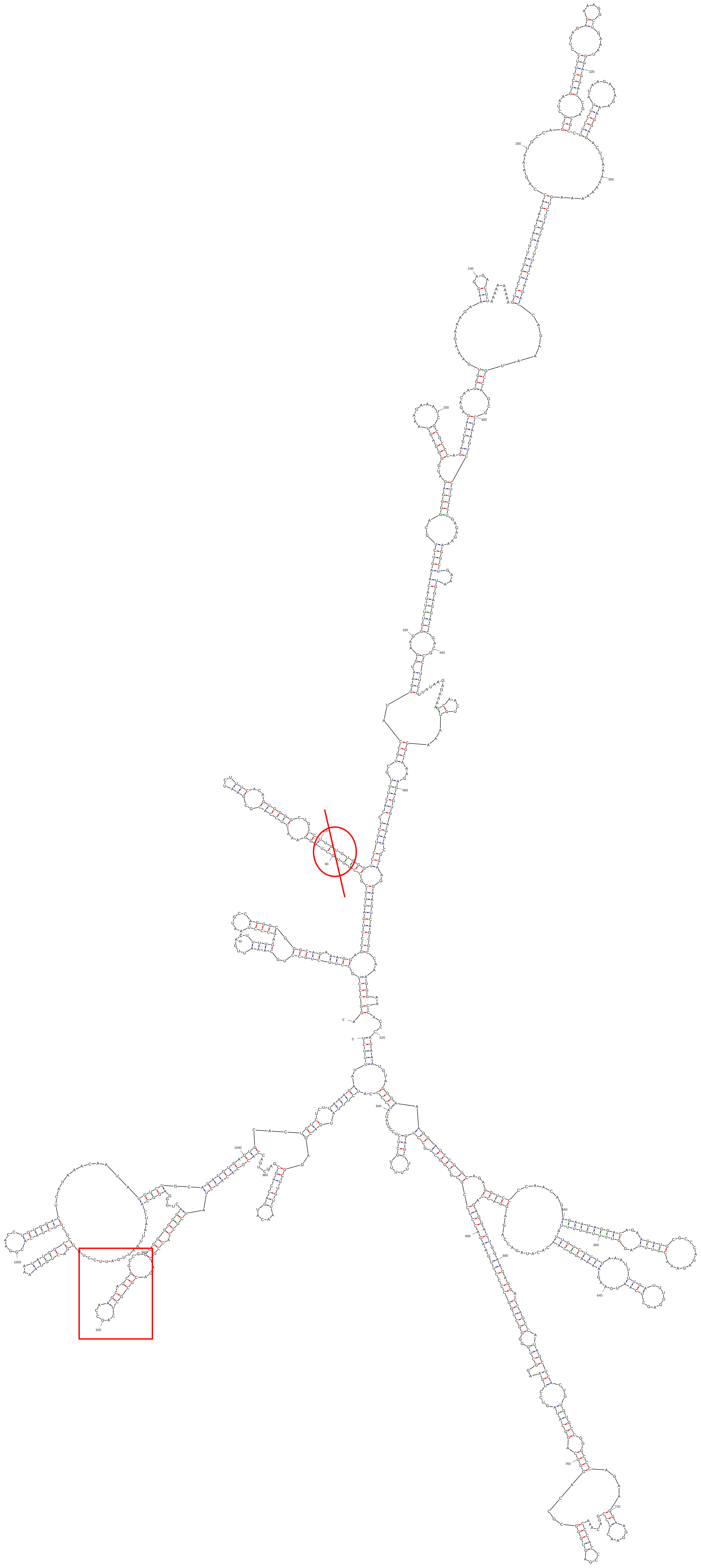

dG = -281.38 [Initially -325.60] rat-full

Output of sir_graph (©)
mfold_util 4.6

Created Thu Mar 10 19:48:46 2011

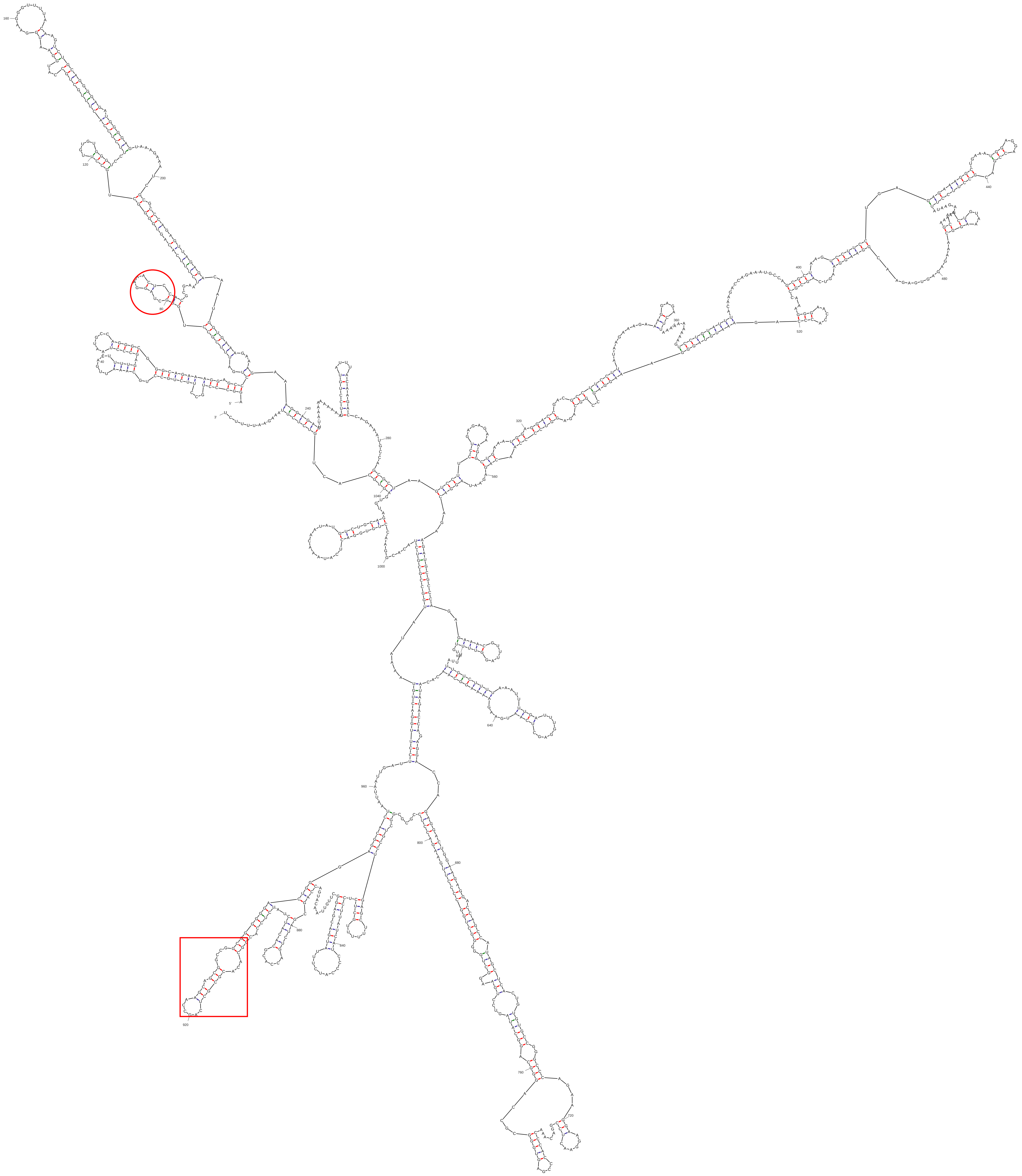

dG = -286.12 [Initially -325.60] rat-full

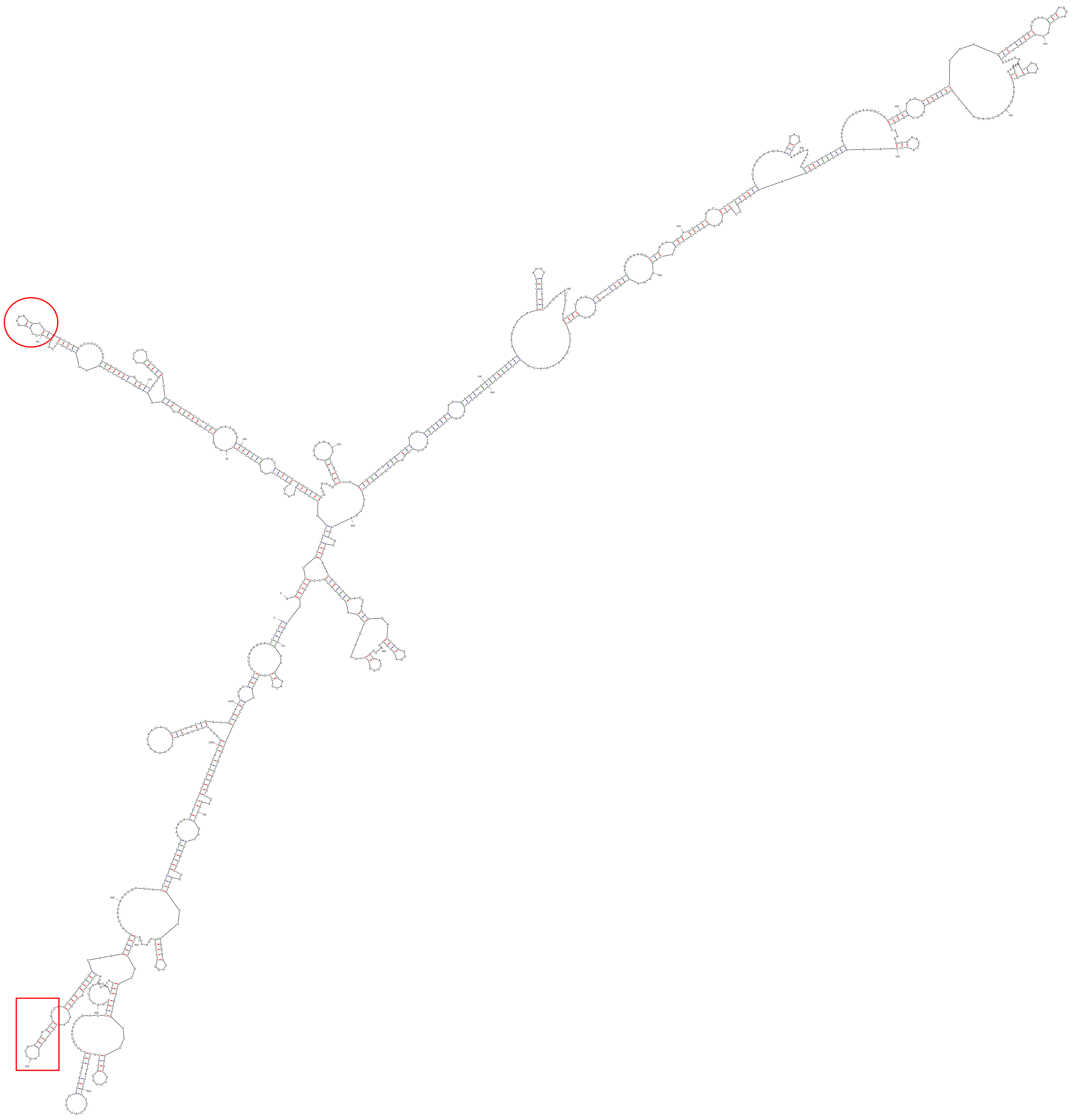

dG = -284.56 [Initially -325.60] rat-full

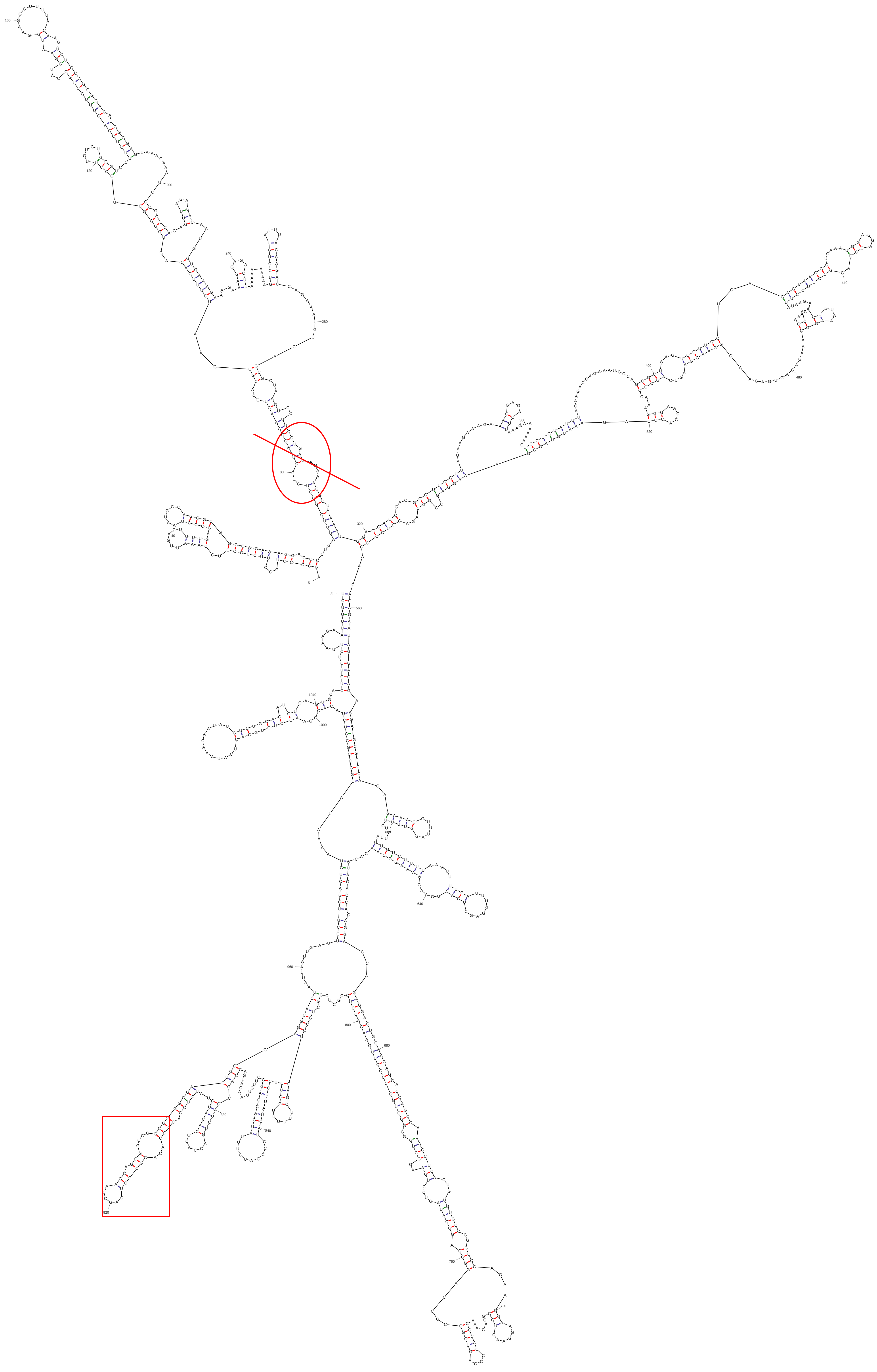

dG = -284.25 [Initially -325.50] rat-full

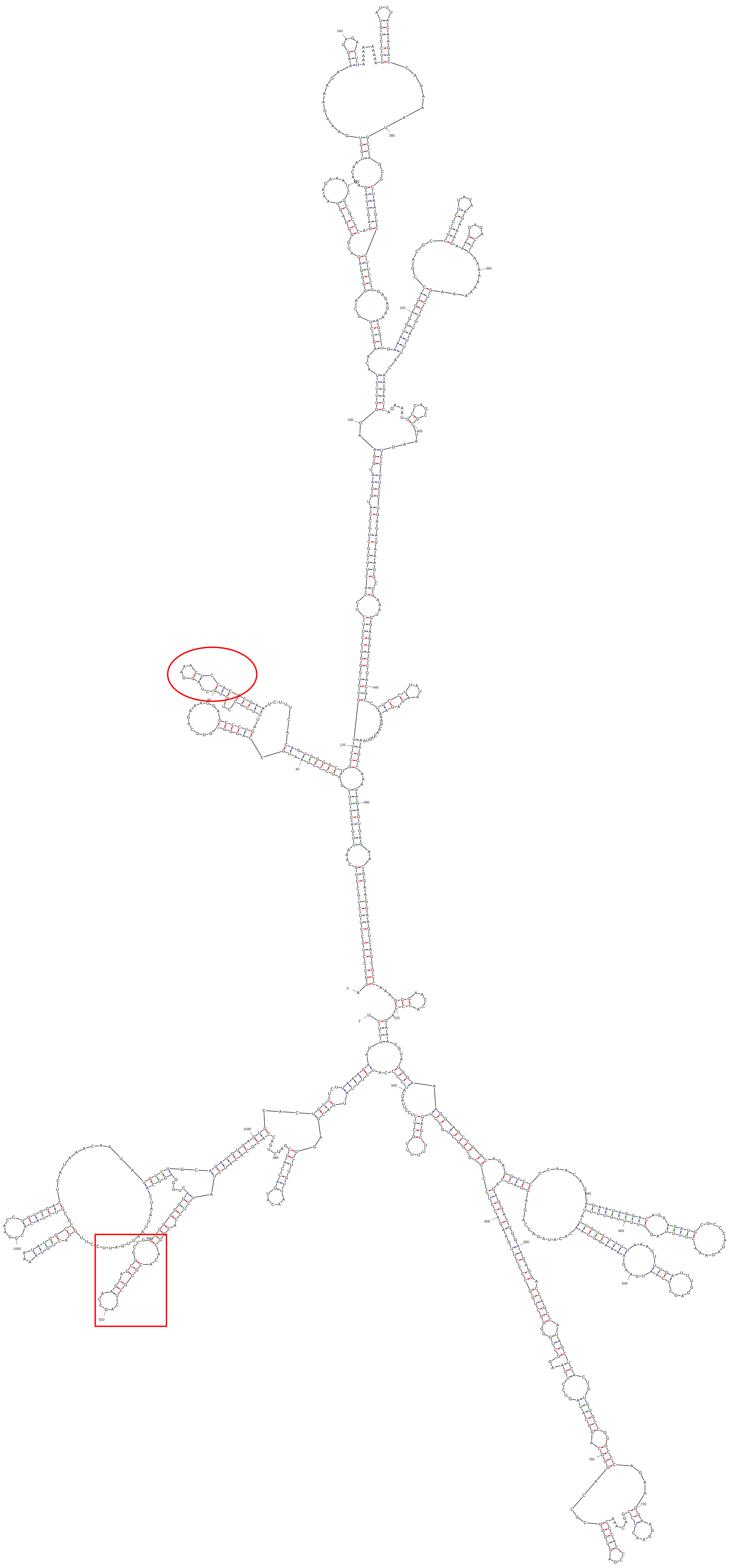

dG = -281.87 [Initially -324.40] rat-full

Output of sir_graph (©)
mfold_util 4.6

Created Thu Mar 10 19:48:47 2011

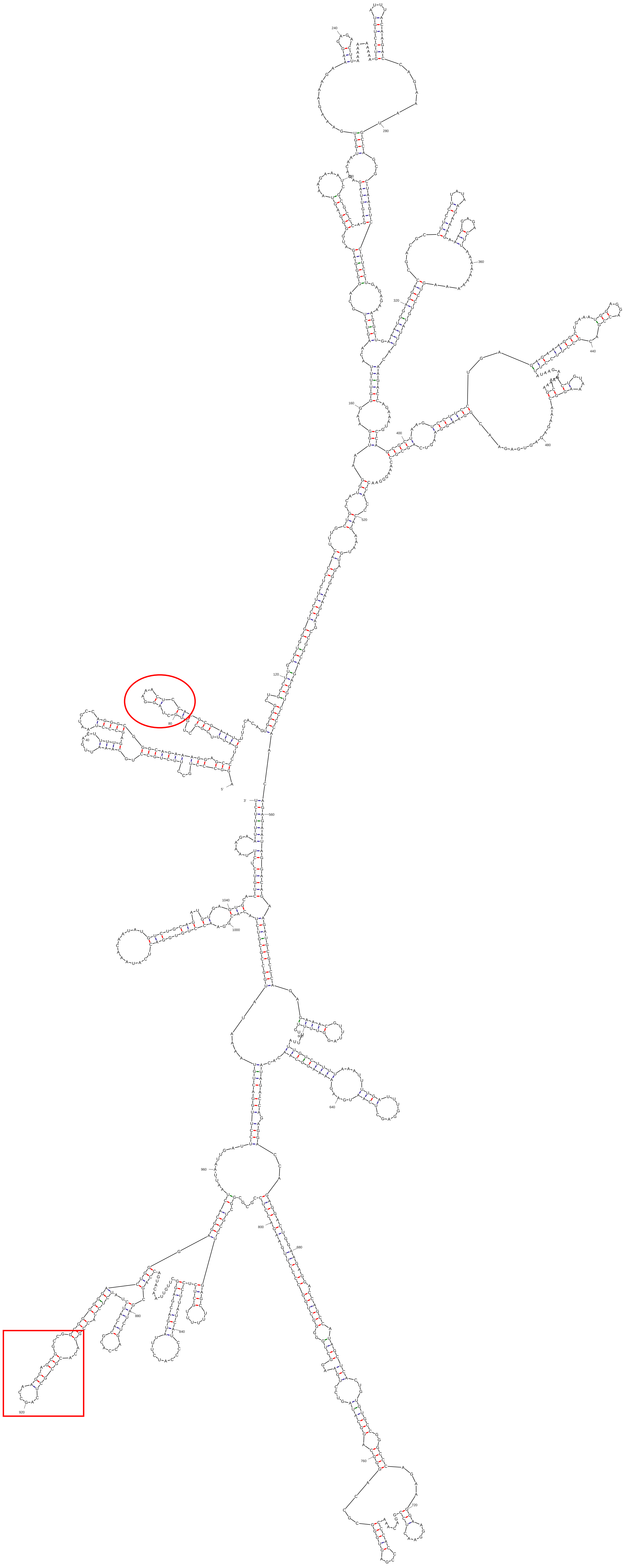

dG = -286.19 [Initially -323.10] rat-full

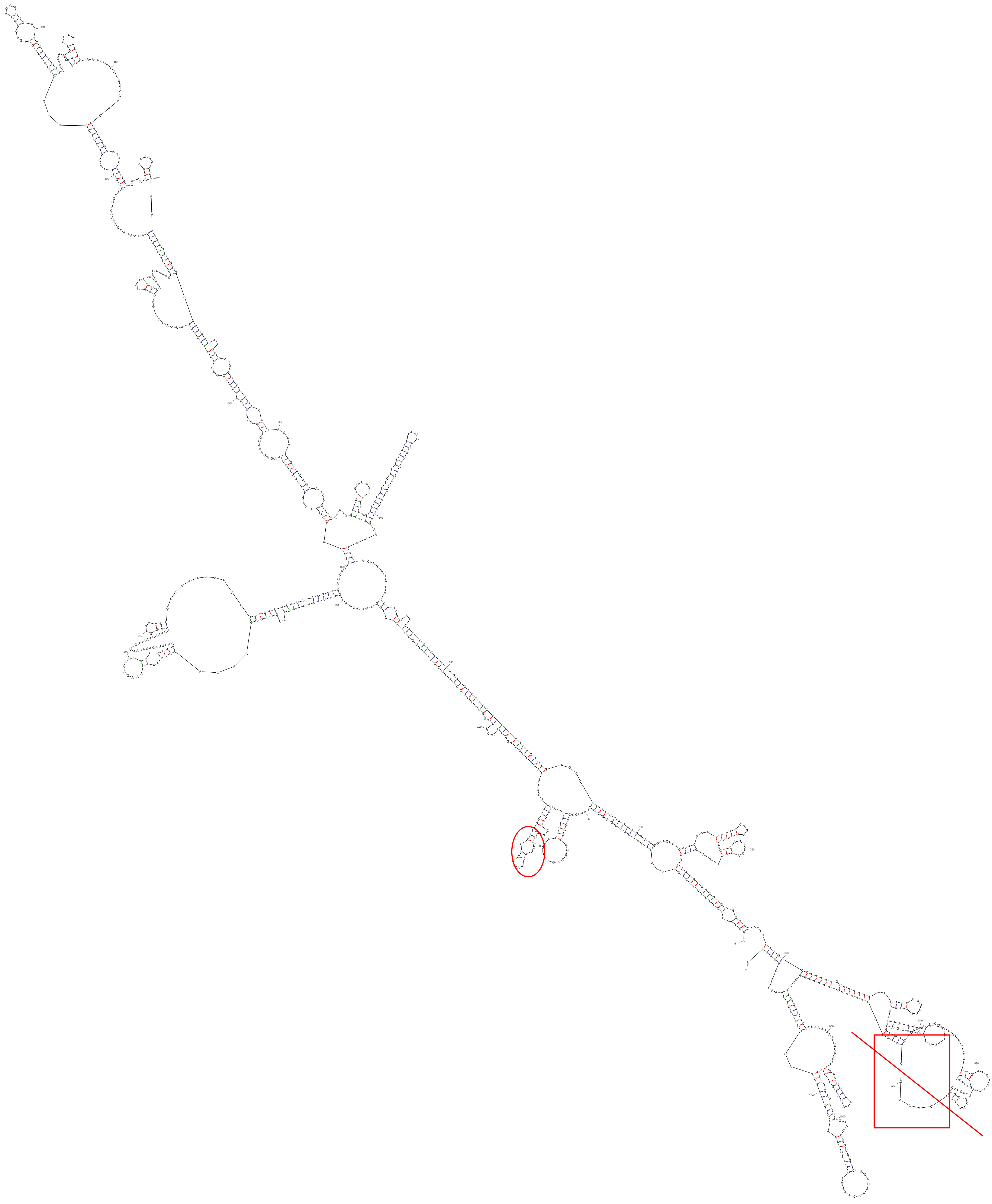

dG = -278.01 [Initially -321.10] rat-full

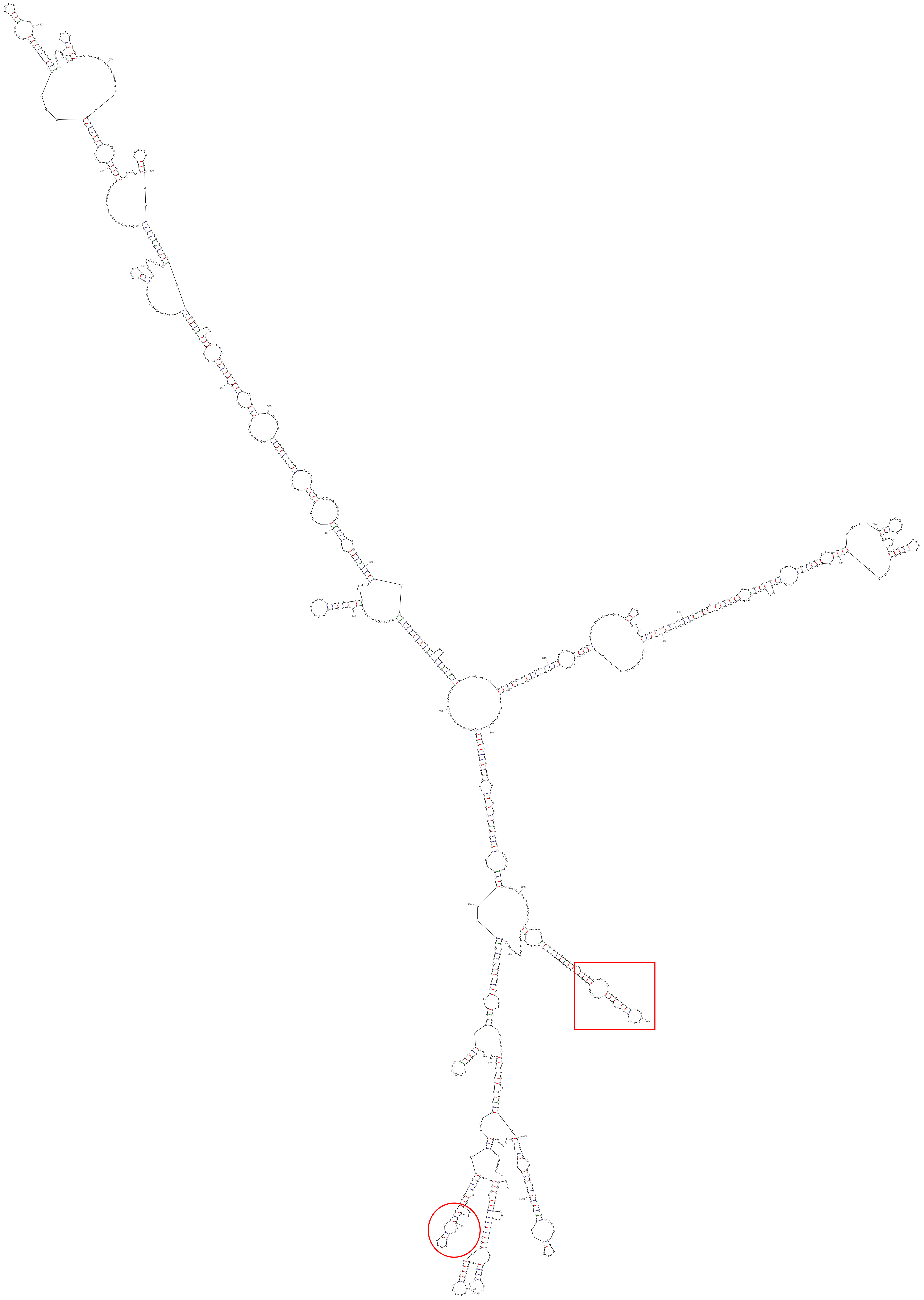

dG = -293.64 [Initially -335.60] rat-full

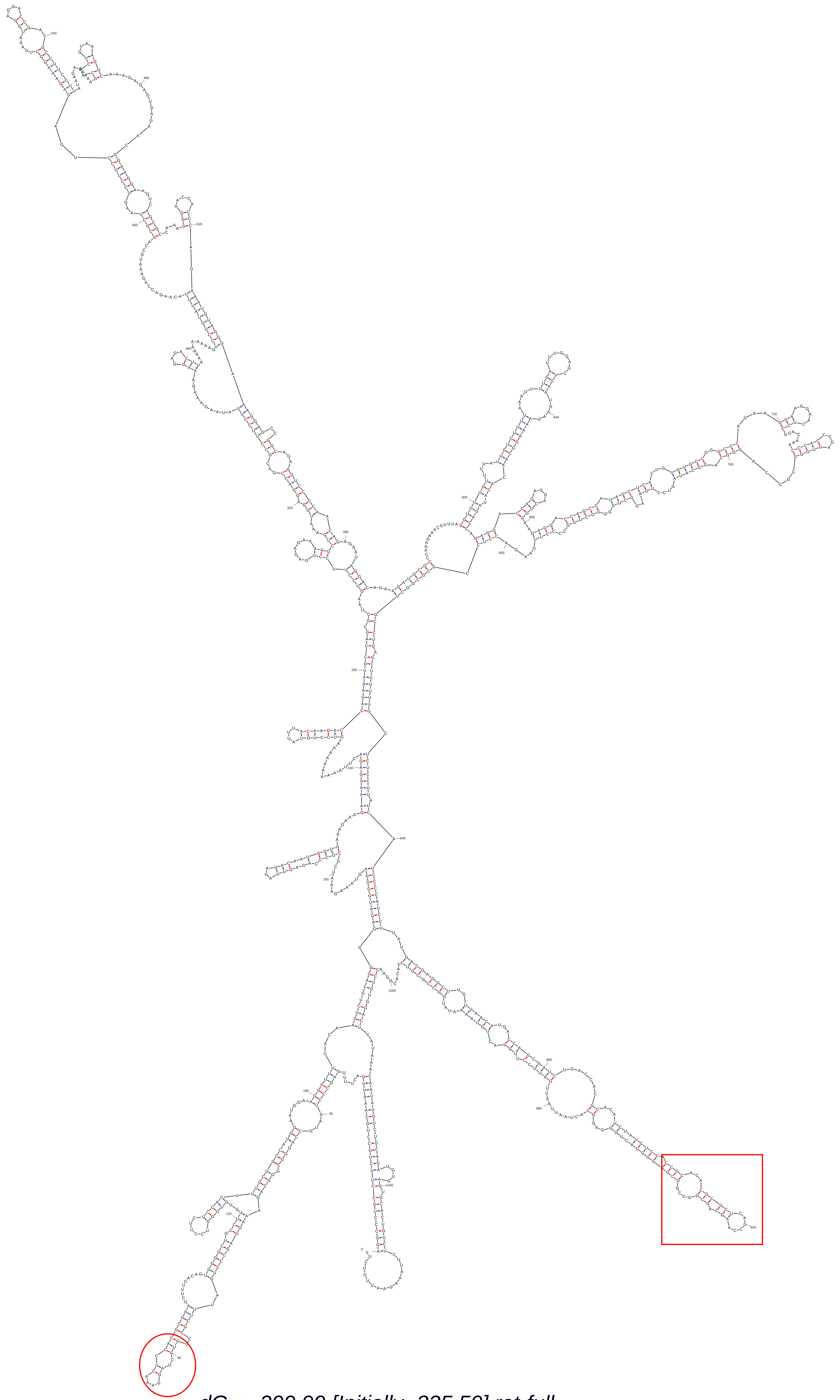

dG = -299.99 [Initially -335.50] rat-full

Output of sir_graph (©)
mfold_util 4.6

Created Thu Mar 10 19:48:49 2011

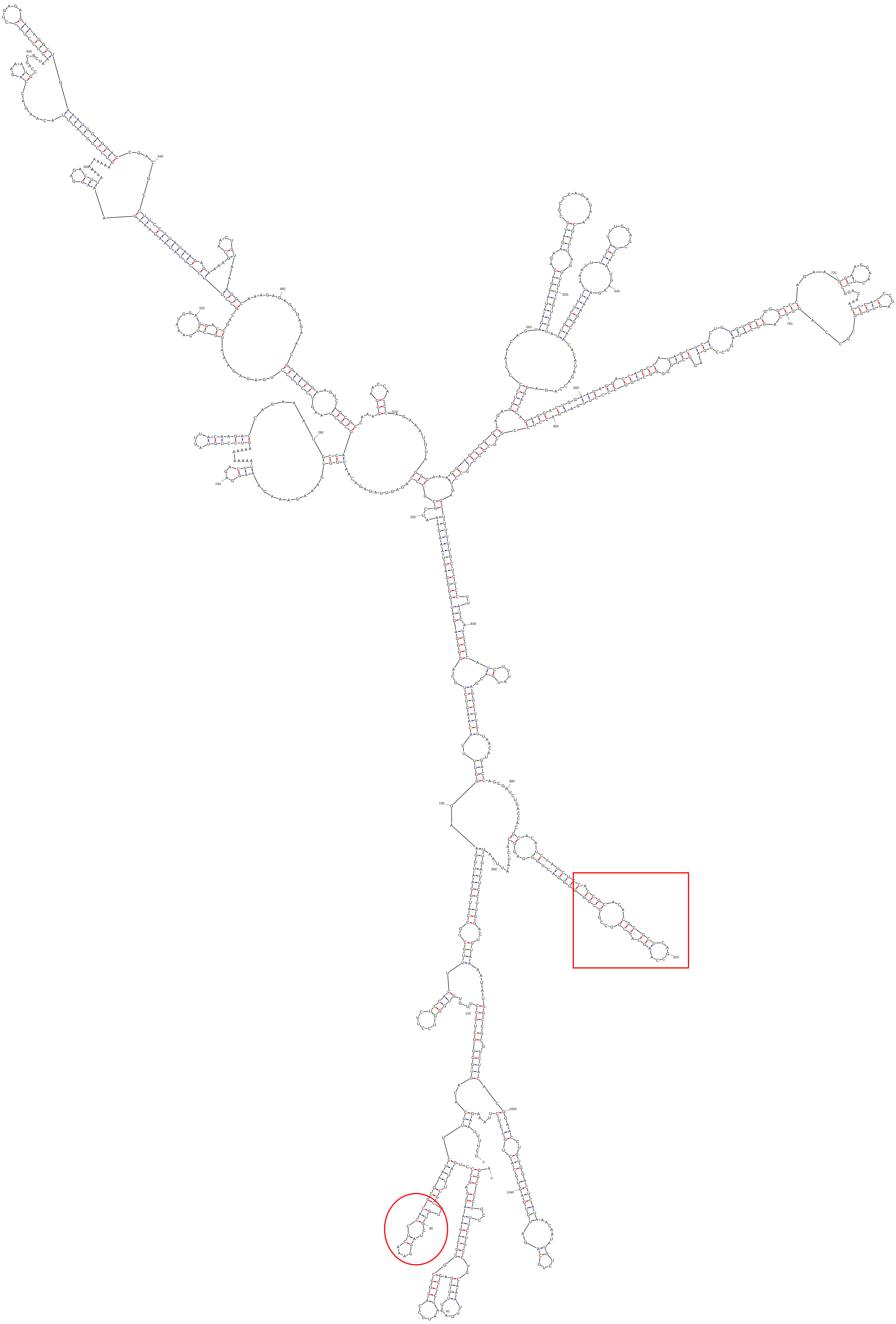

dG = -286.20 [Initially -334.80] rat-full

Output of sir_graph (©)
mfold_util 4.6

Created Thu Mar 10 19:48:49 2011

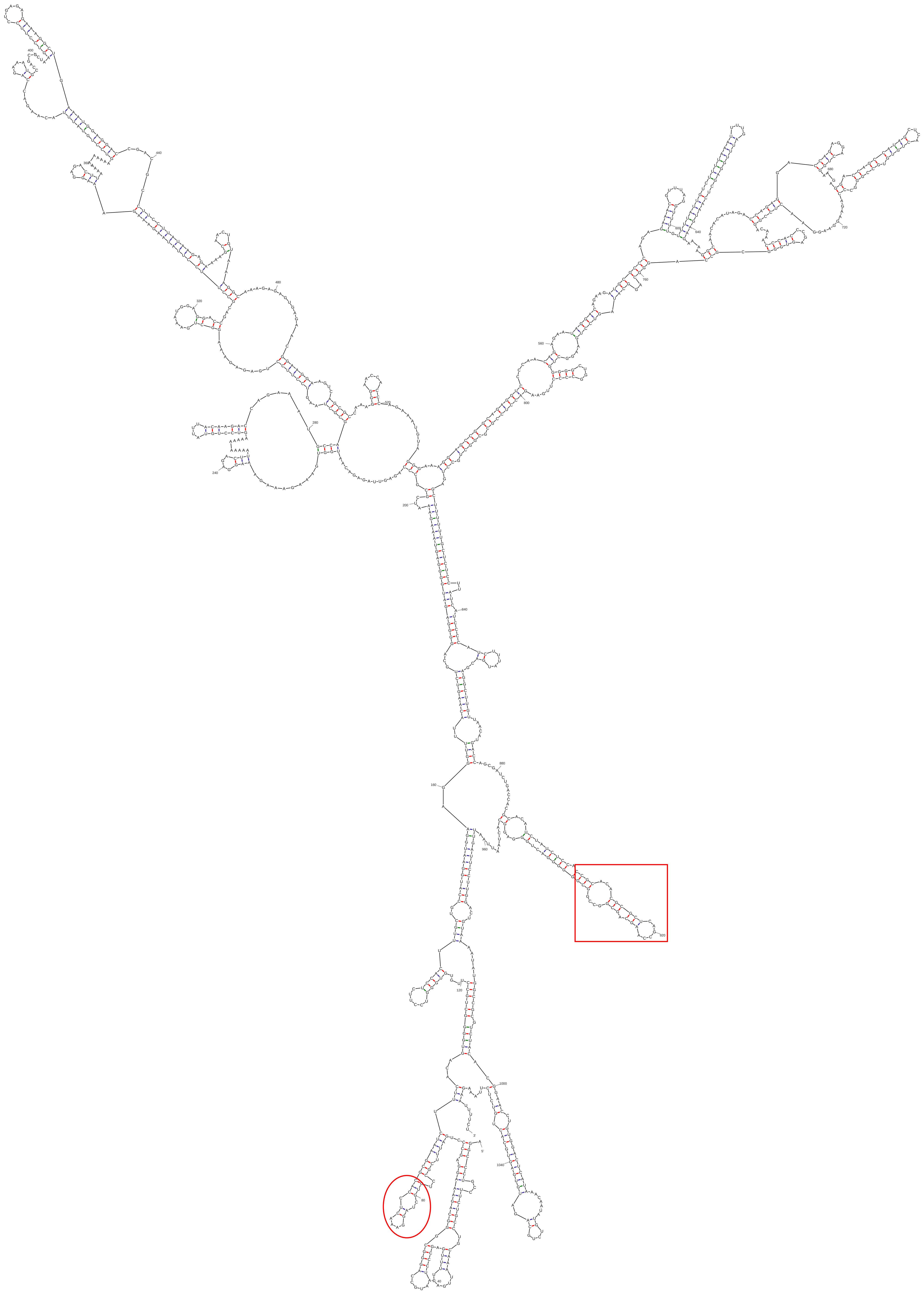

dG = -284.86 [Initially -334.50] rat-full

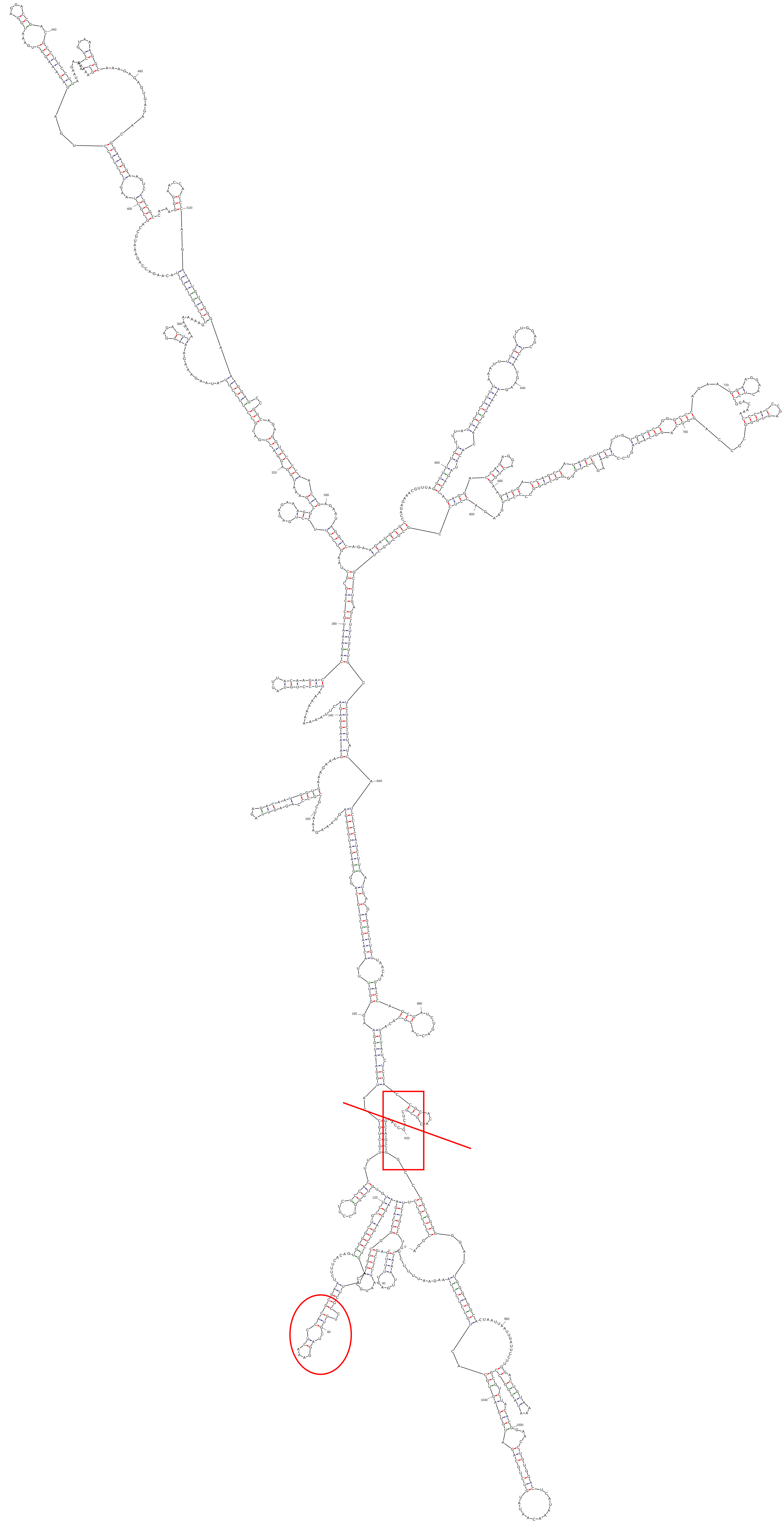

dG = -293.62 [Initially -334.00] rat-full

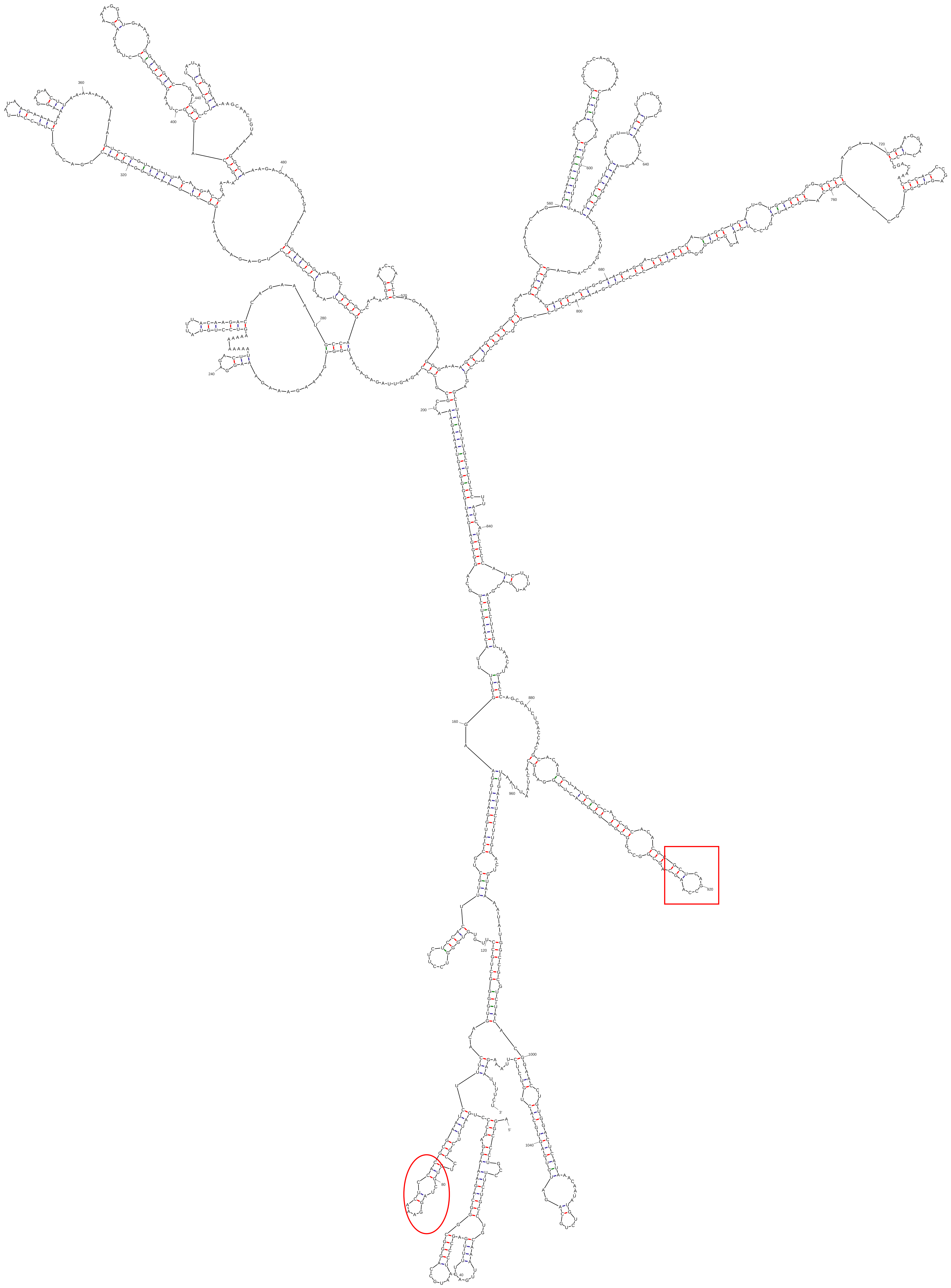

dG = -286.40 [Initially -333.80] rat-full

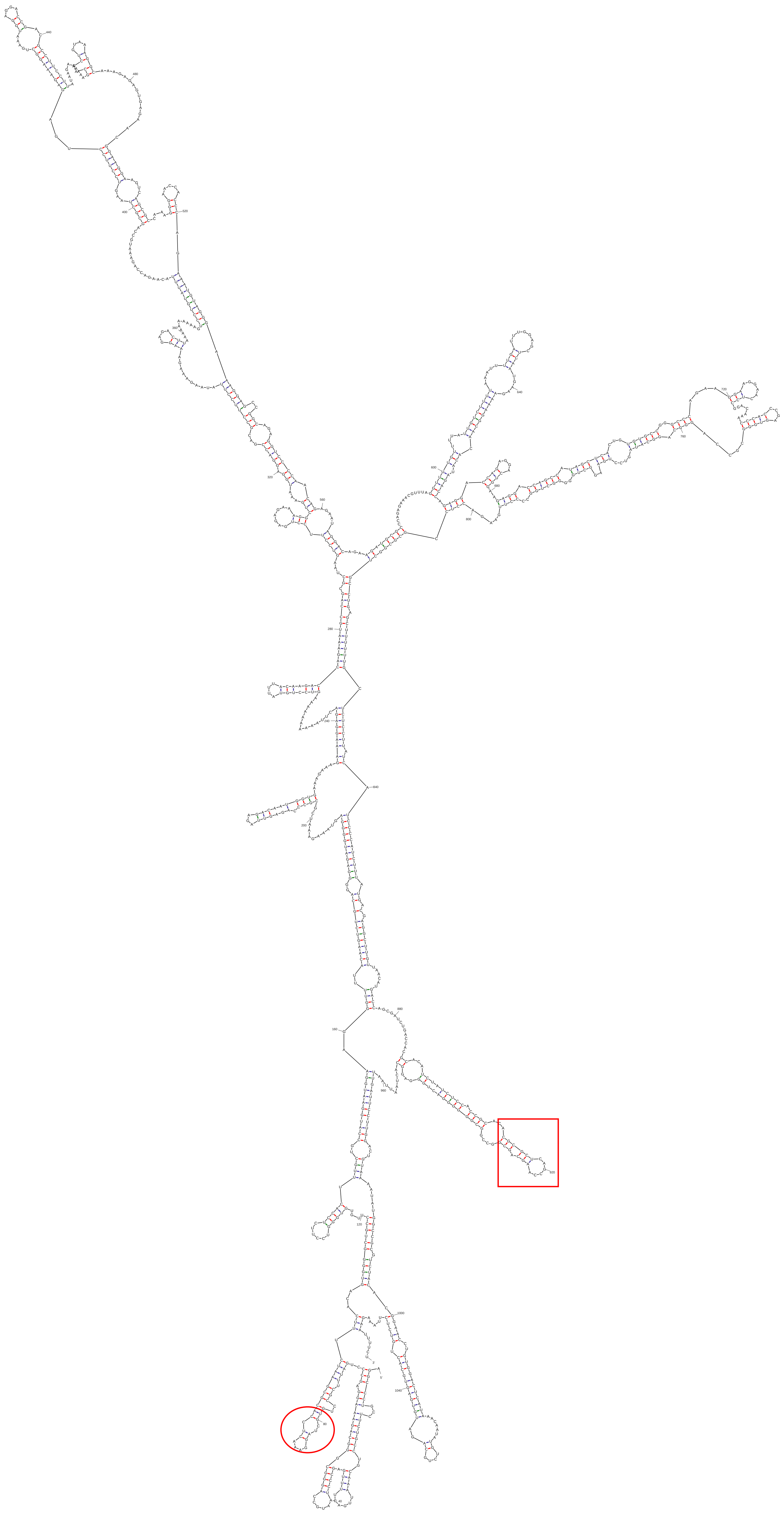

dG = -294.43 [Initially -336.90] rat-full

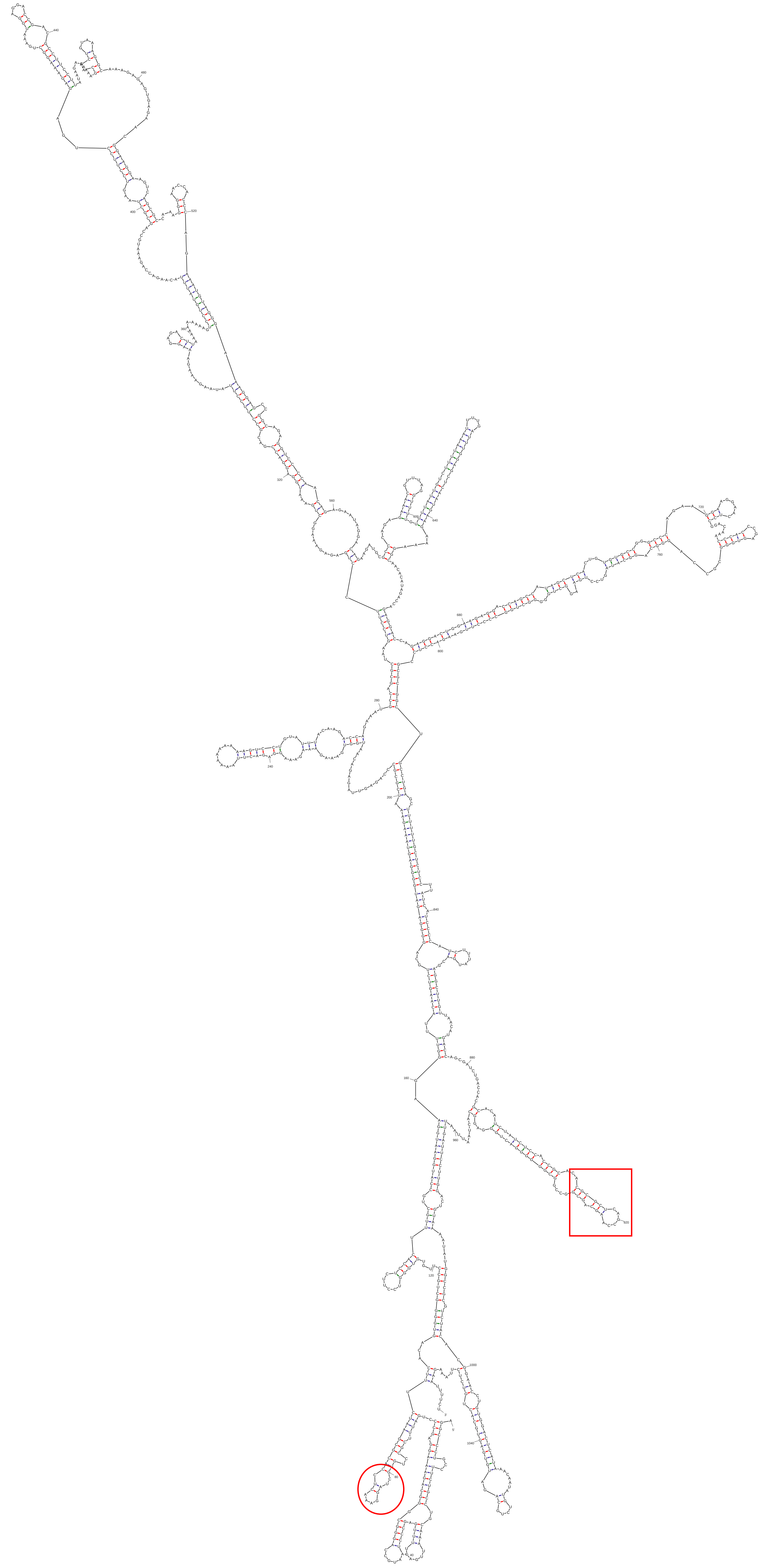

dG = -293.29 [Initially -336.00] rat-full

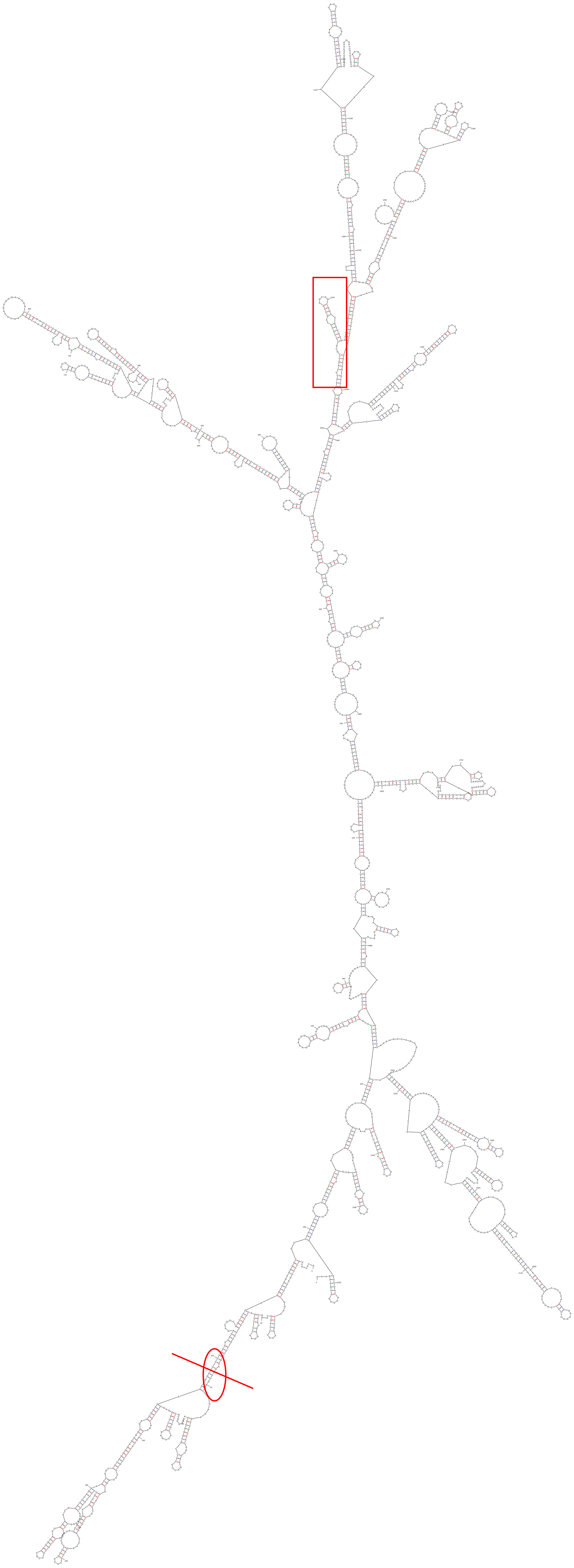

dG = -686.40 [Initially -772.00] human-full

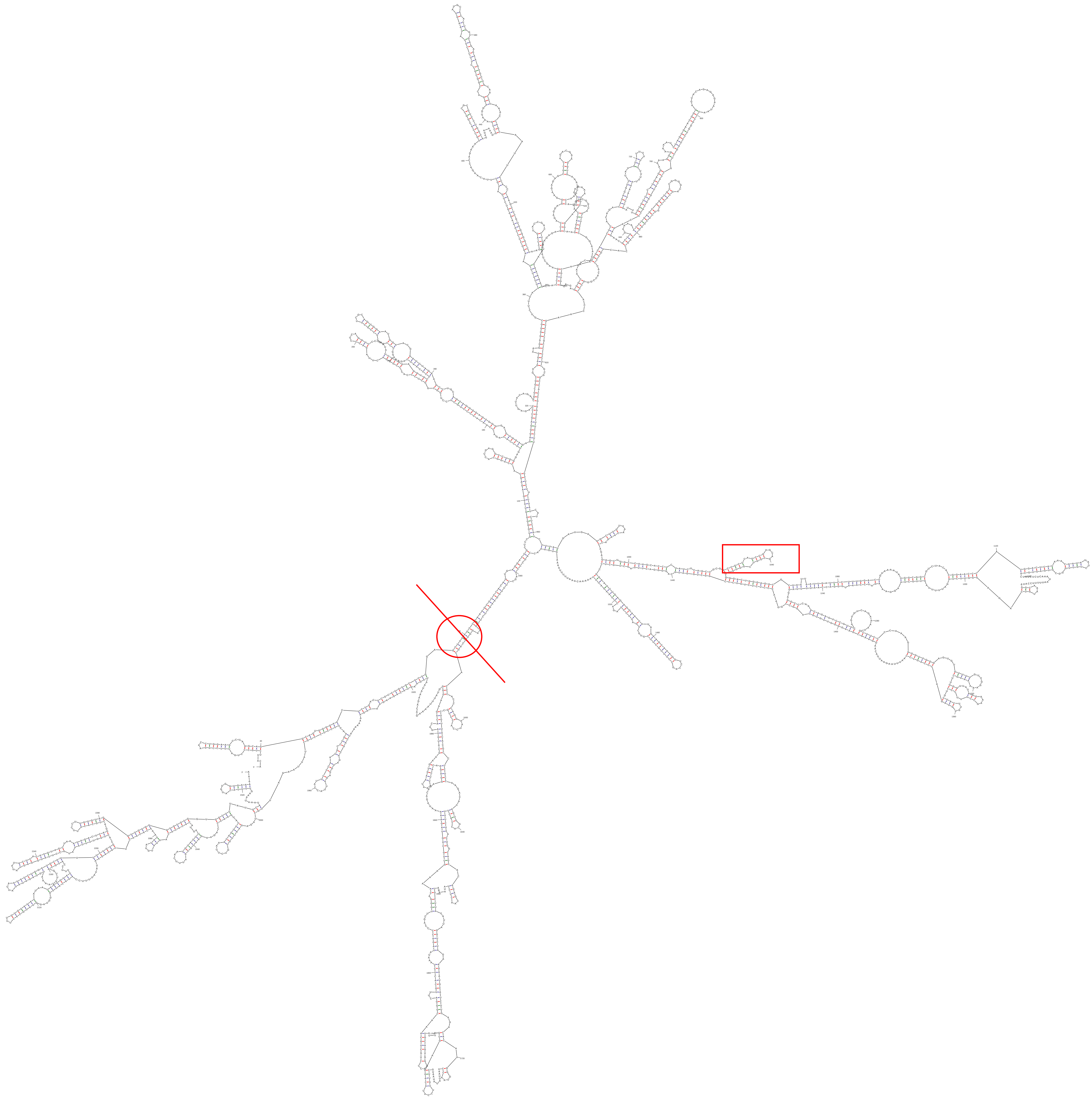

dG = -686.53 [Initially -768.60] human-full

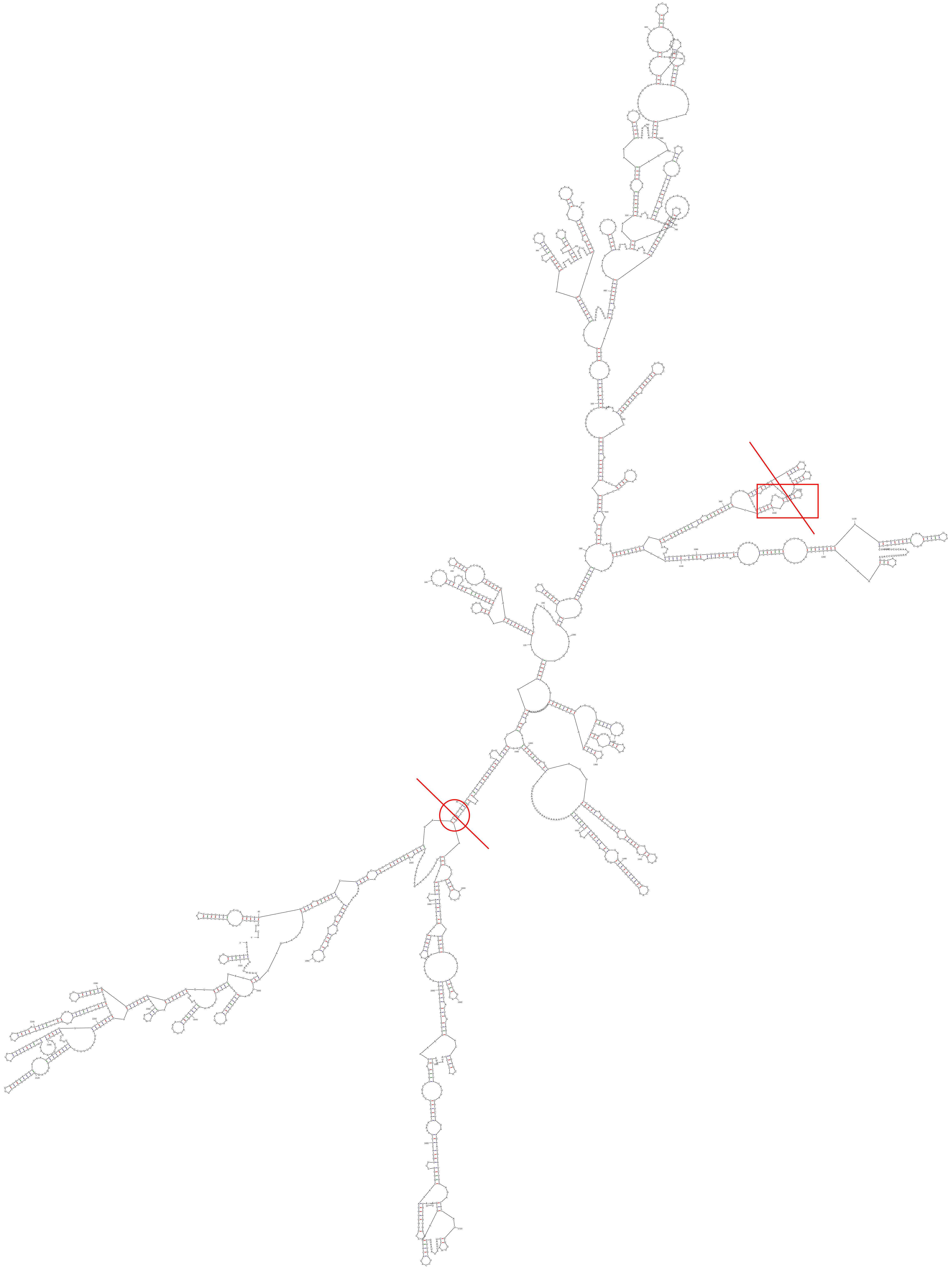

dG = -665.36 [Initially -768.50] human-full

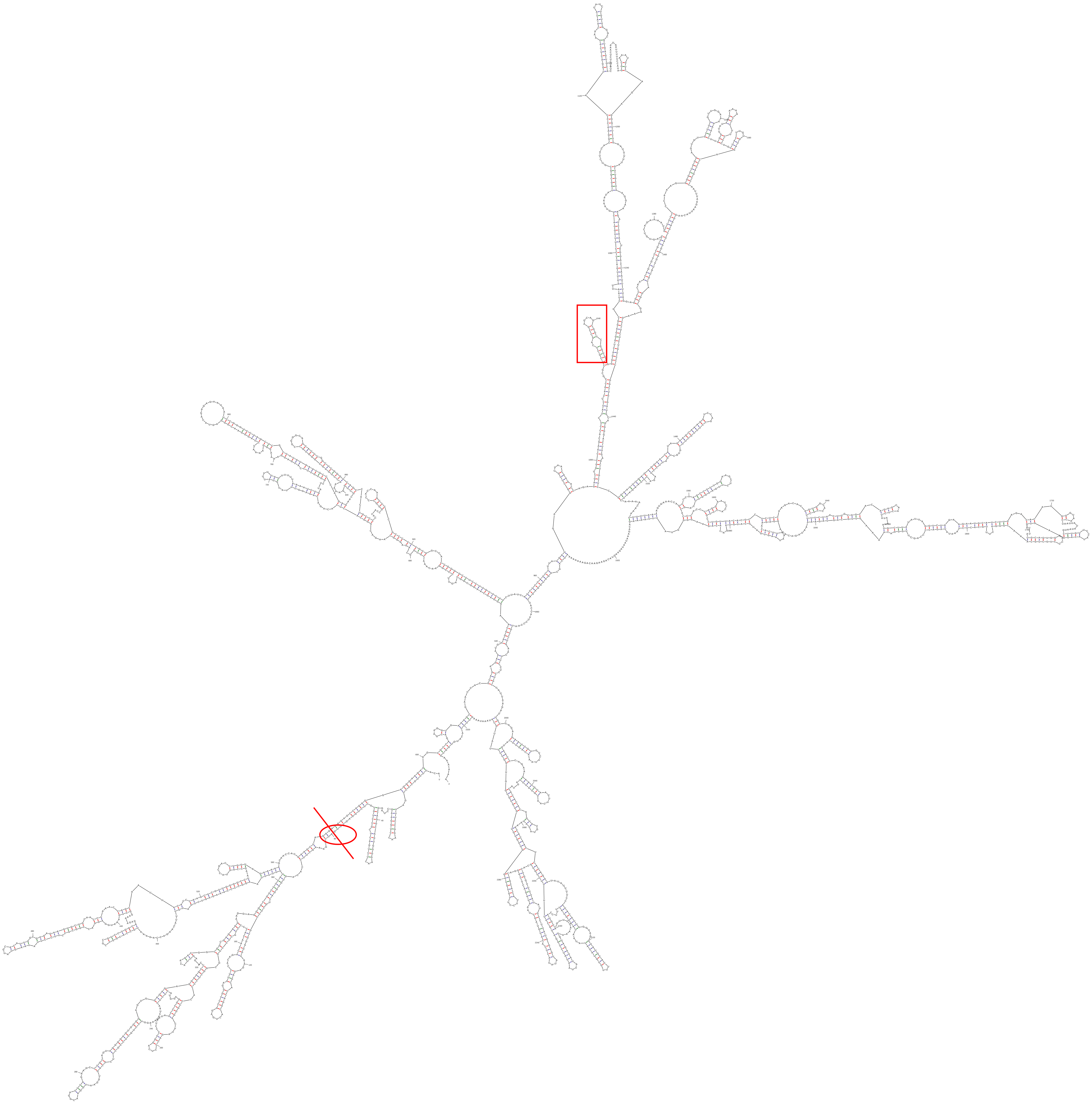

dG = -683.72 [Initially -768.50] human-full

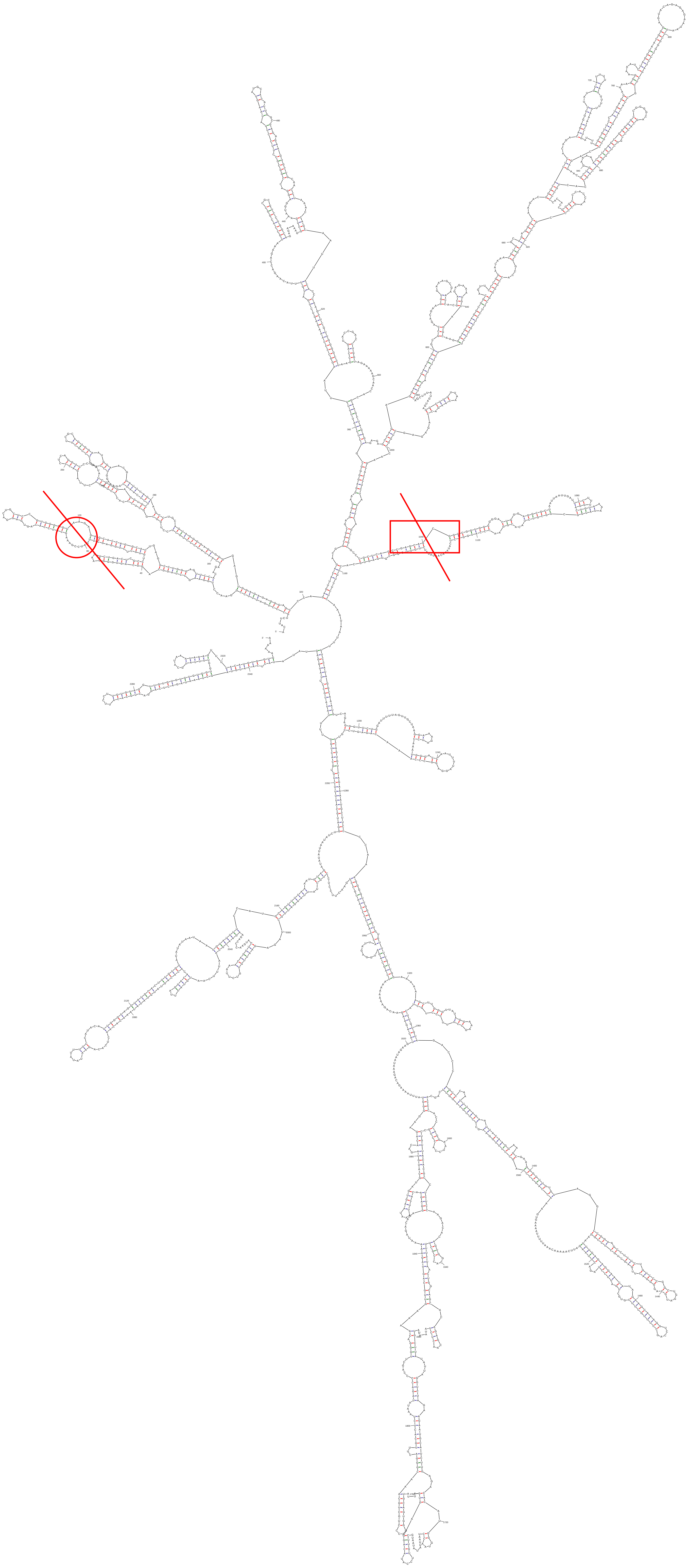

dG = -666.95 [Initially -767.90] human-full

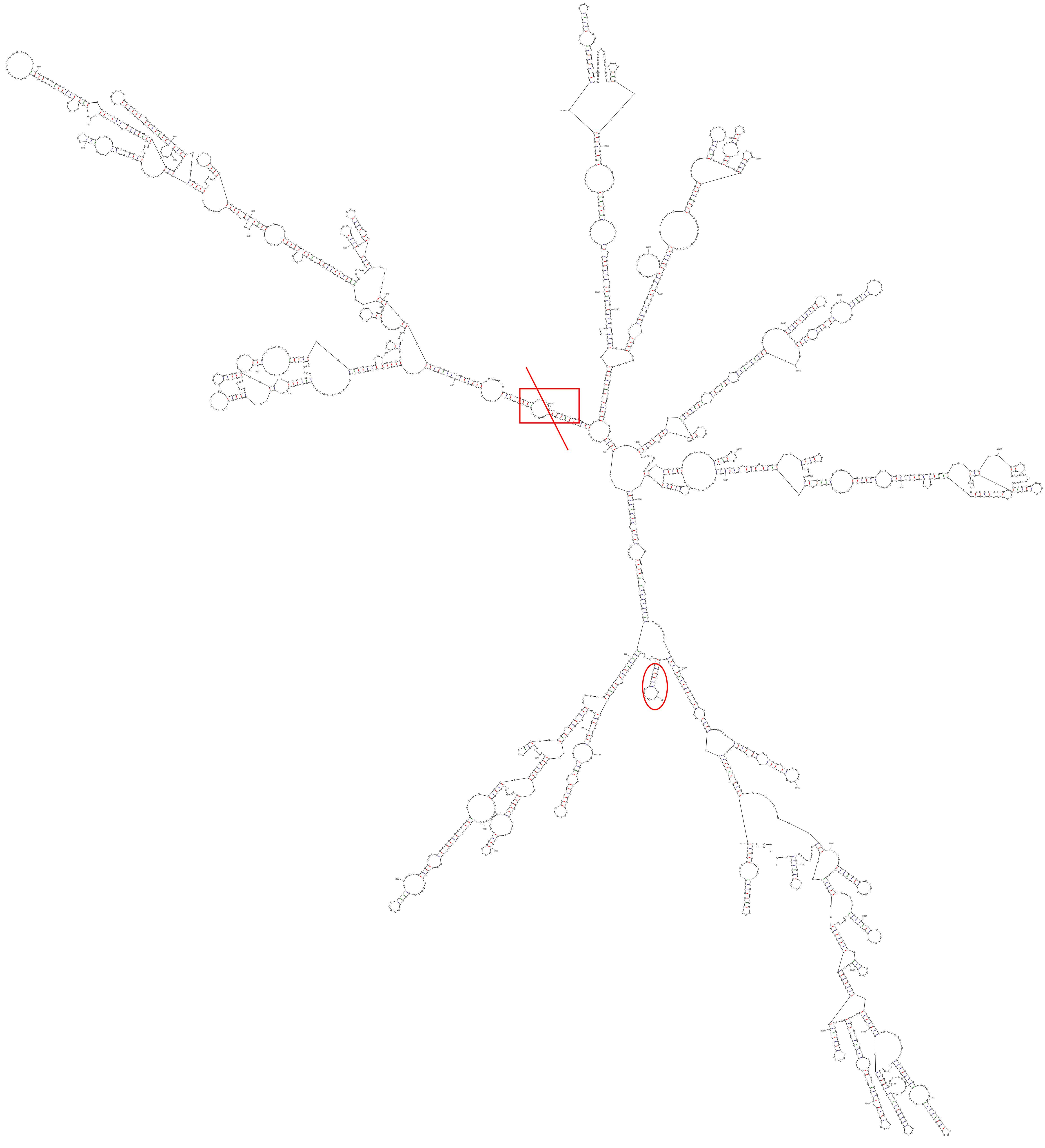

dG = -678.13 [Initially -767.70] human-full

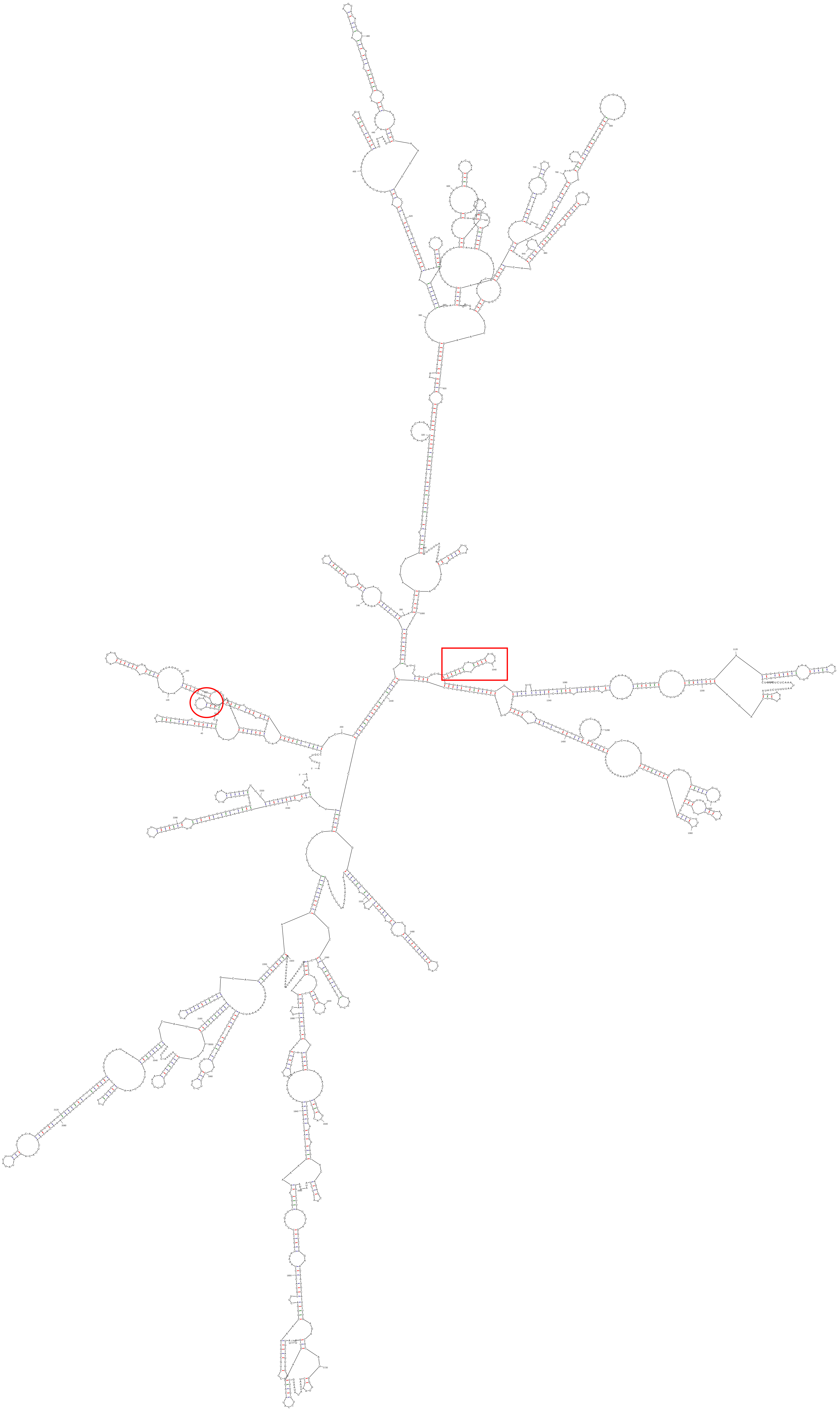

dG = -679.67 [Initially -767.60] human-full

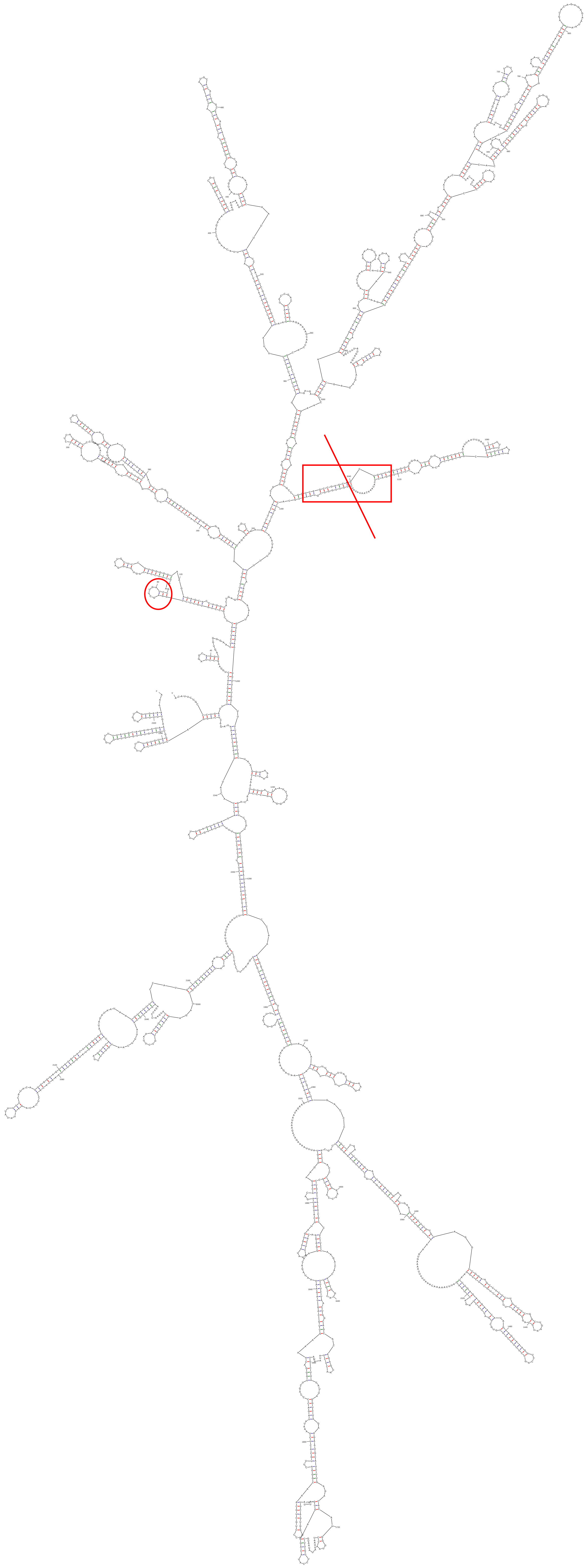

dG = -668.98 [Initially -767.10] human-full

Output of sir_graph (©)
mfold_util 4.6

Created Thu Mar 10 19:49:00 2011

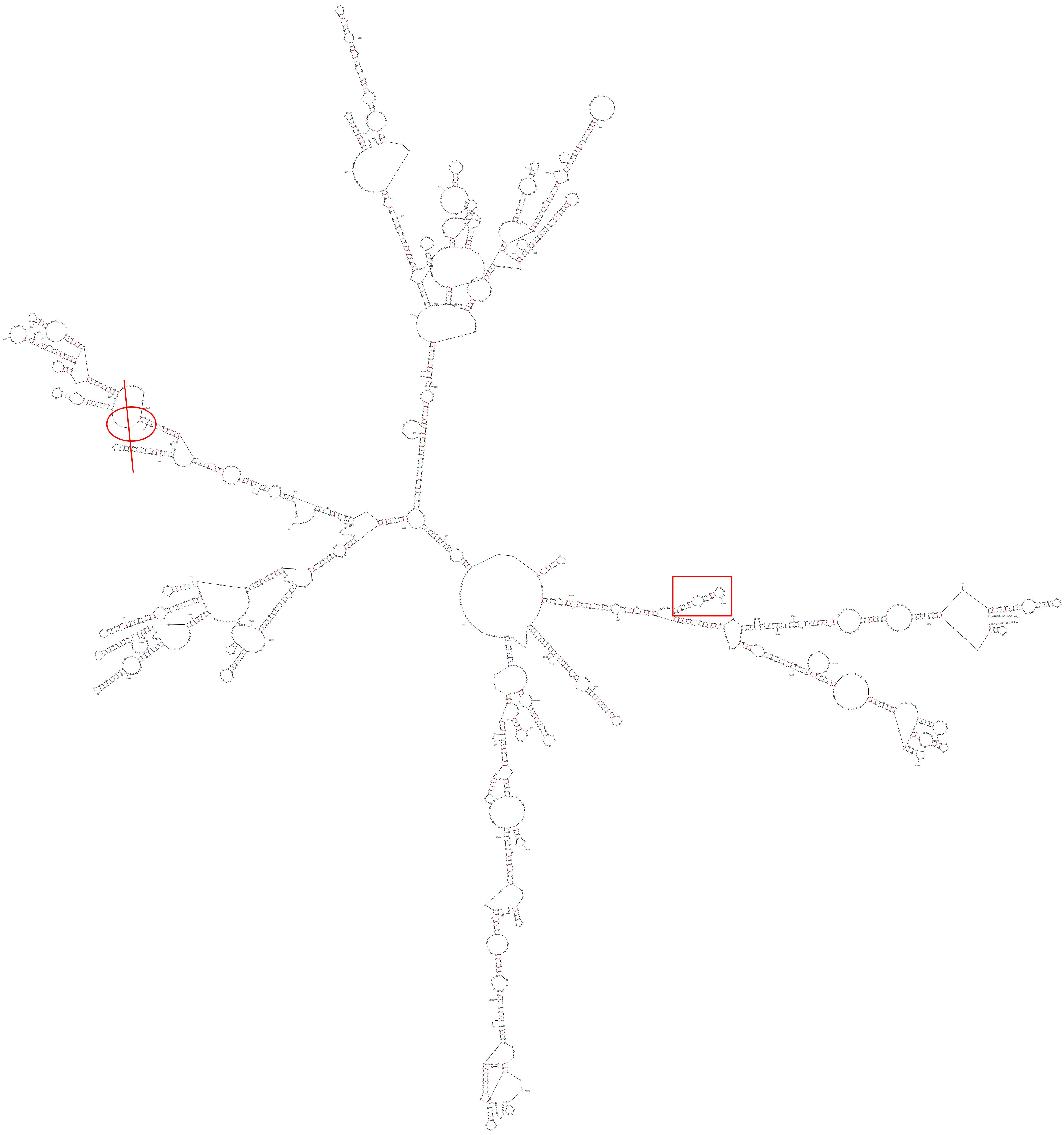

dG = -680.76 [Initially -766.70] human-full

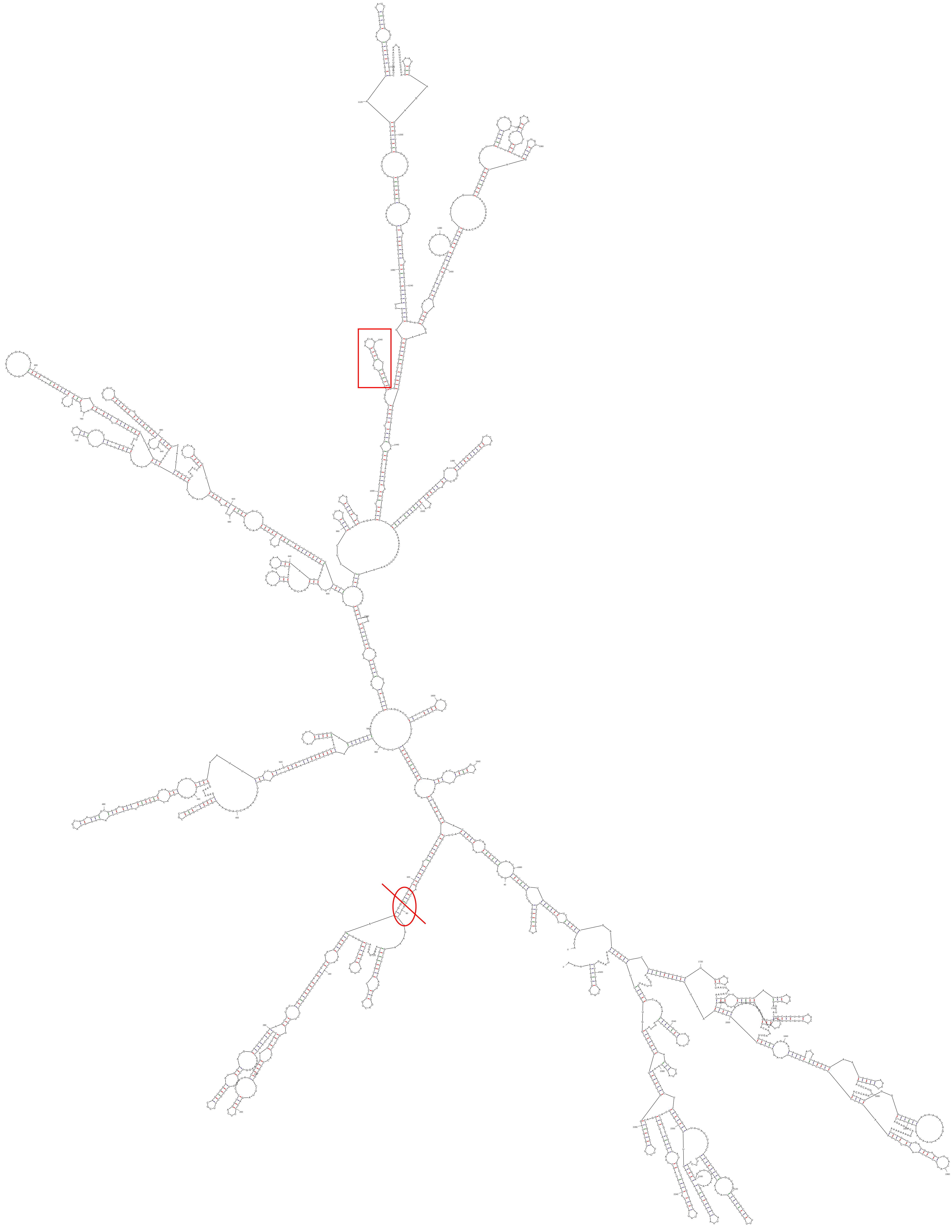

dG = -691.59 [Initially -766.60] human-full

Output of sir_graph (©)
mfold_util 4.6

Created Thu Mar 10 19:49:01 2011

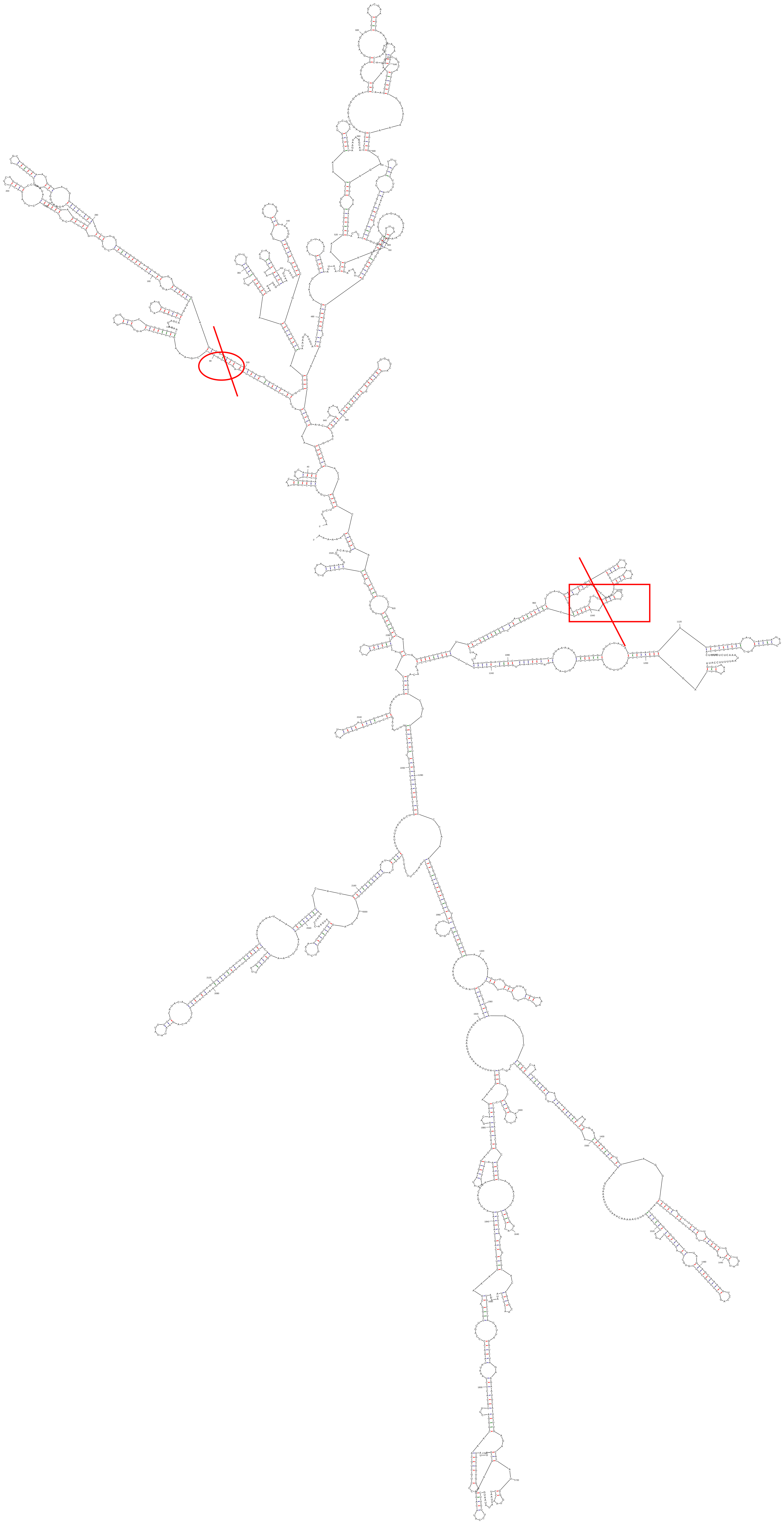

dG = -660.29 [Initially -765.80] human-full

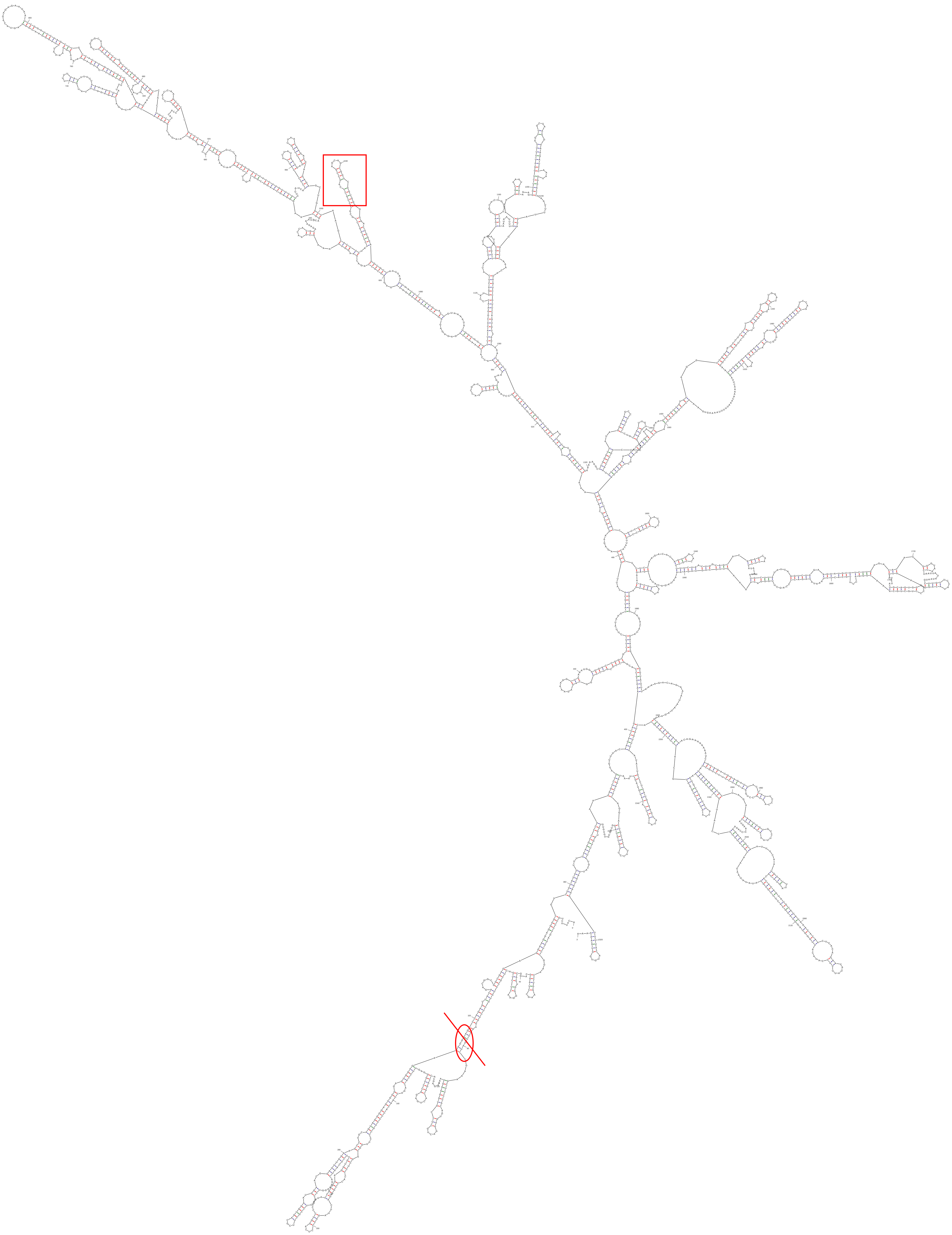

dG = -677.68 [Initially -771.80] human-full

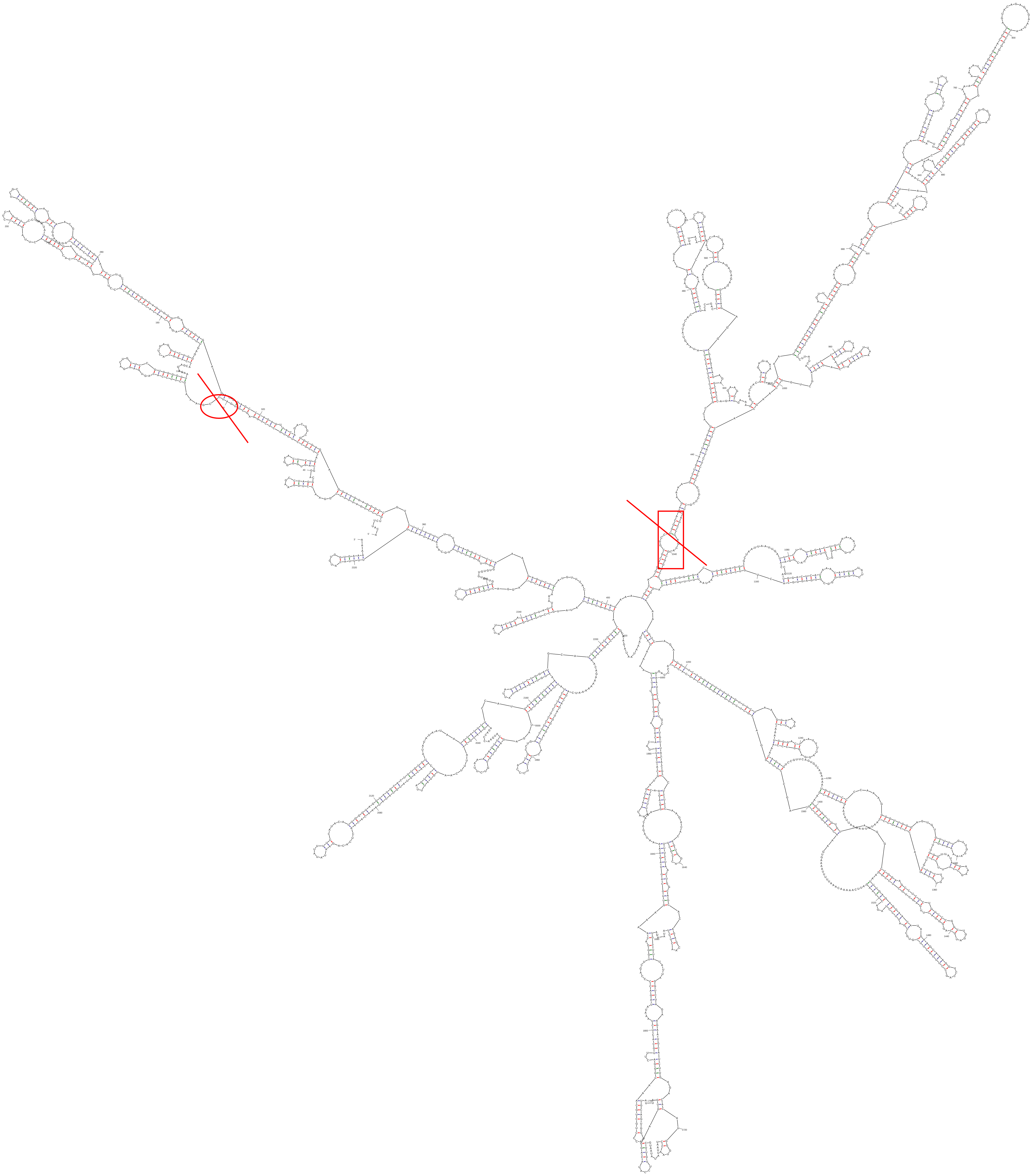

dG = -664.43 [Initially -765.10] human-full

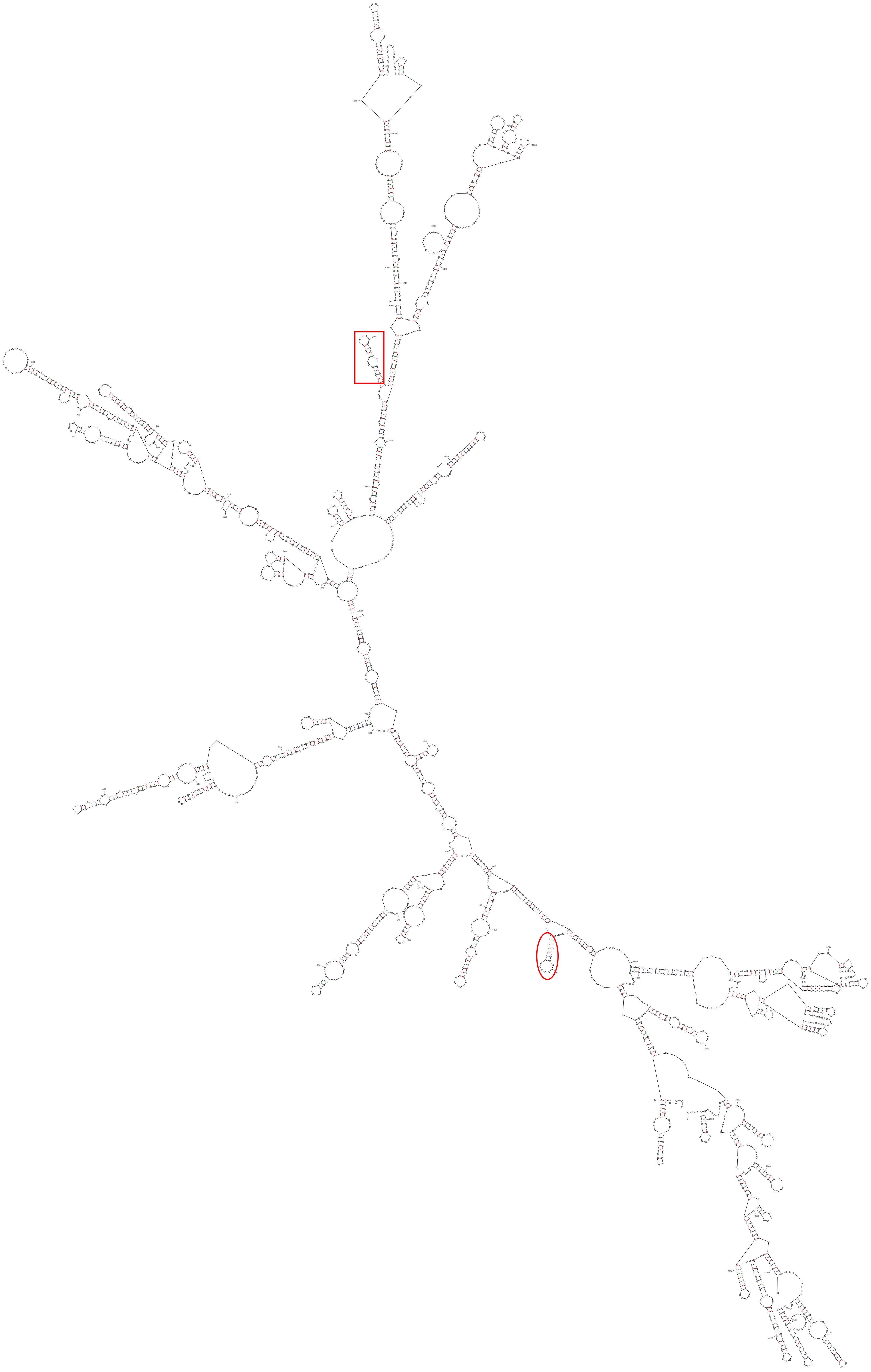

dG = -673.97 [Initially -765.10] human-full

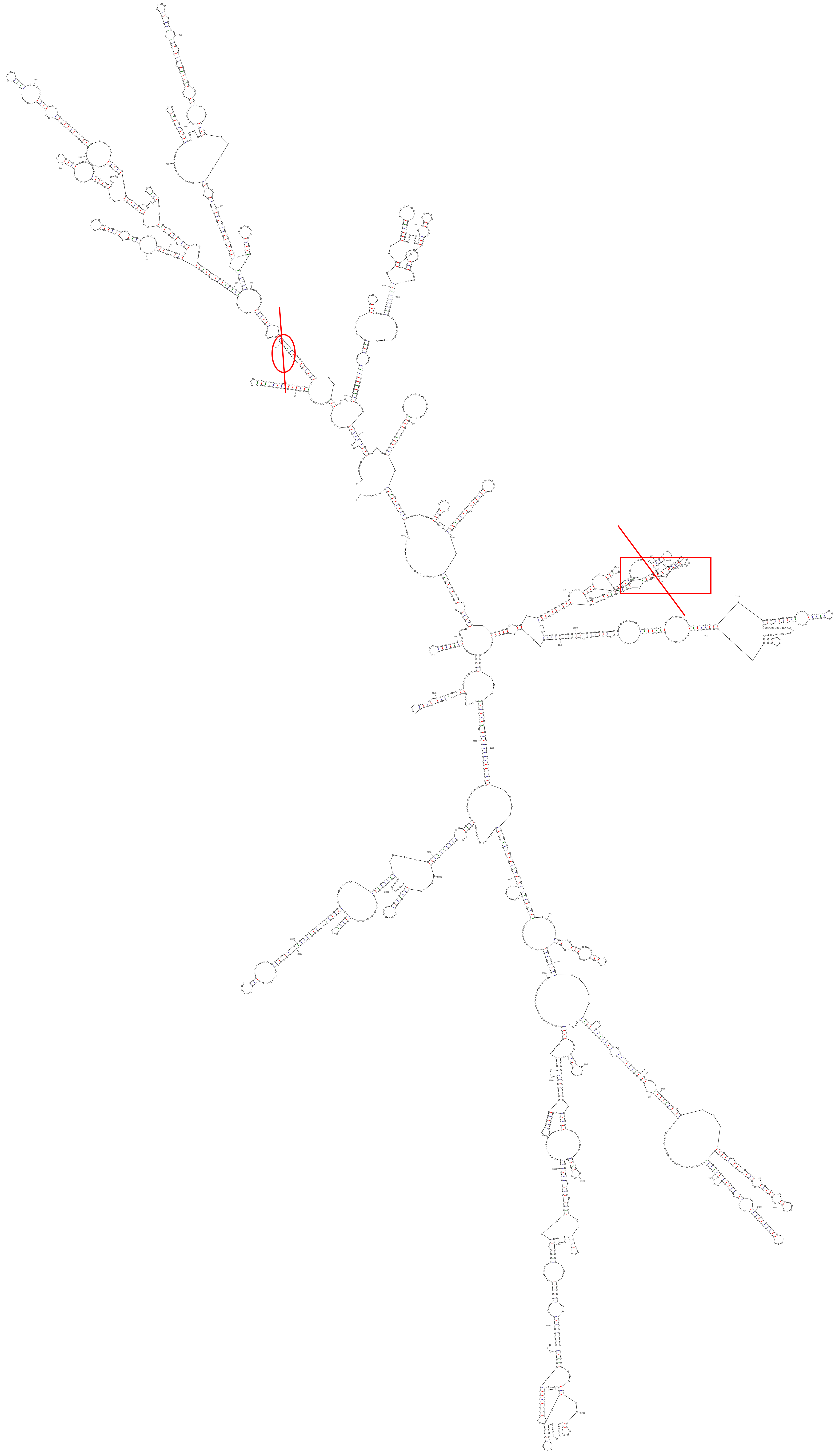

dG = -664.79 [Initially -765.00] human-full

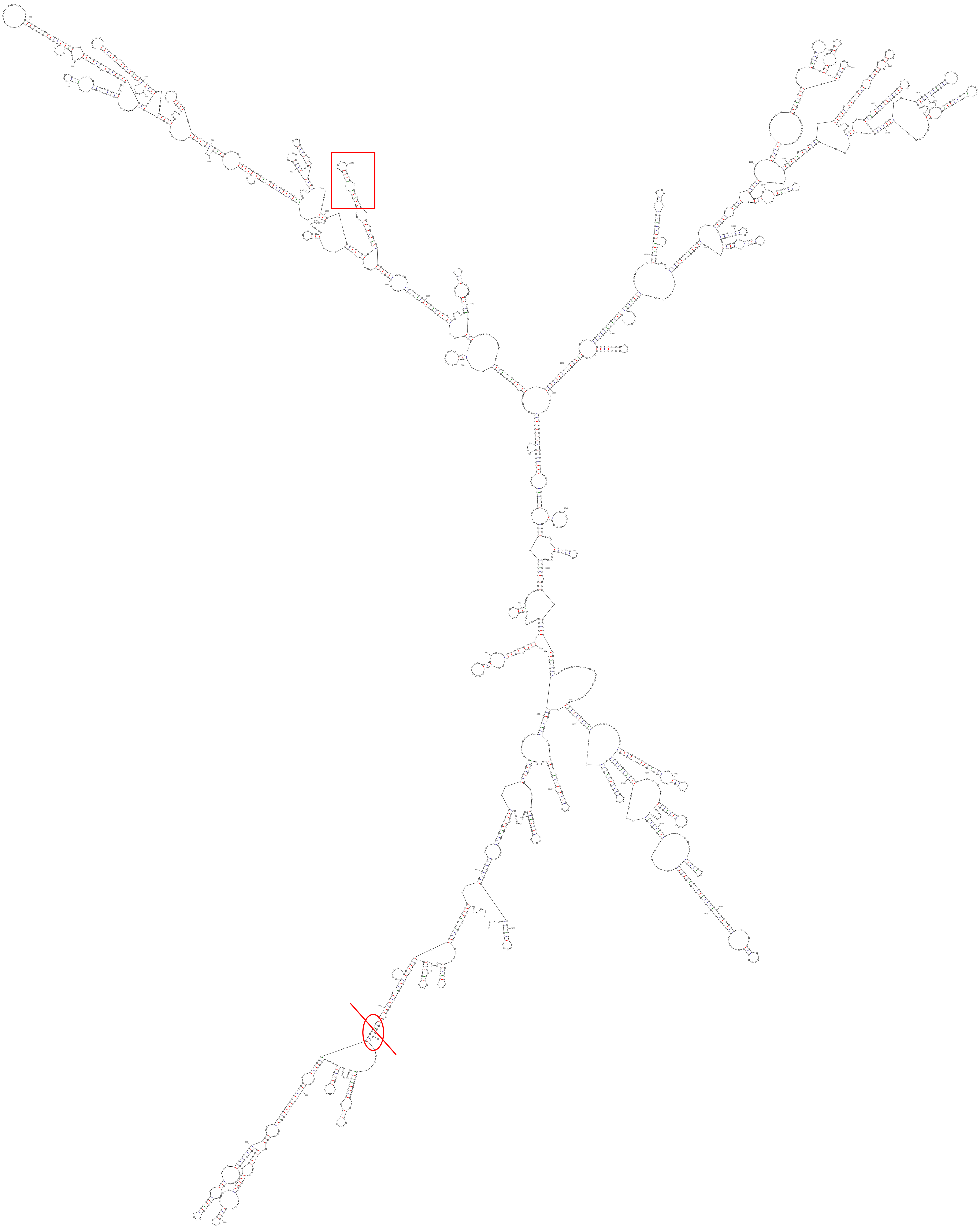

dG = -666.93 [Initially -765.00] human-full

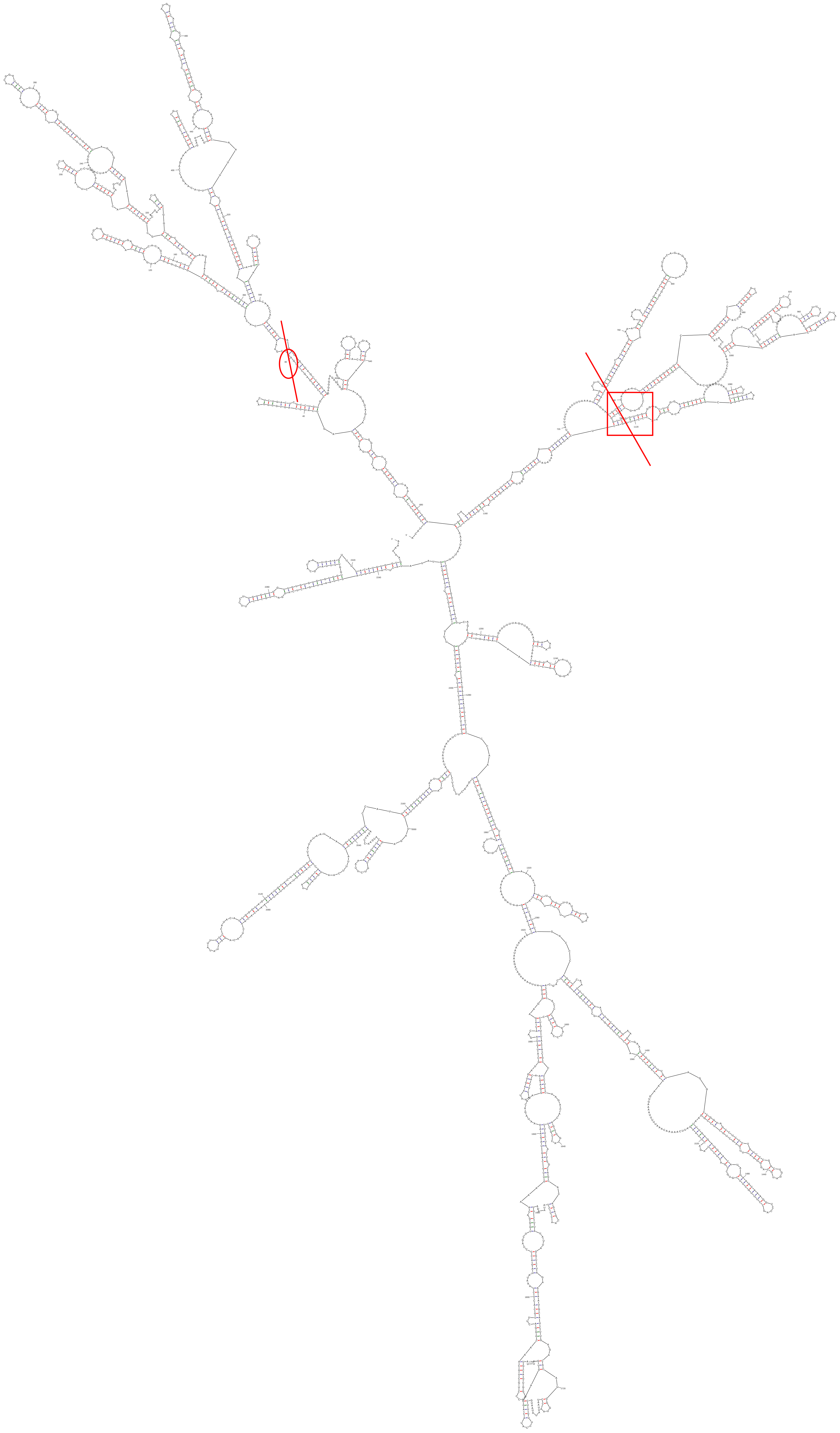

dG = -672.41 [Initially -764.90] human-full

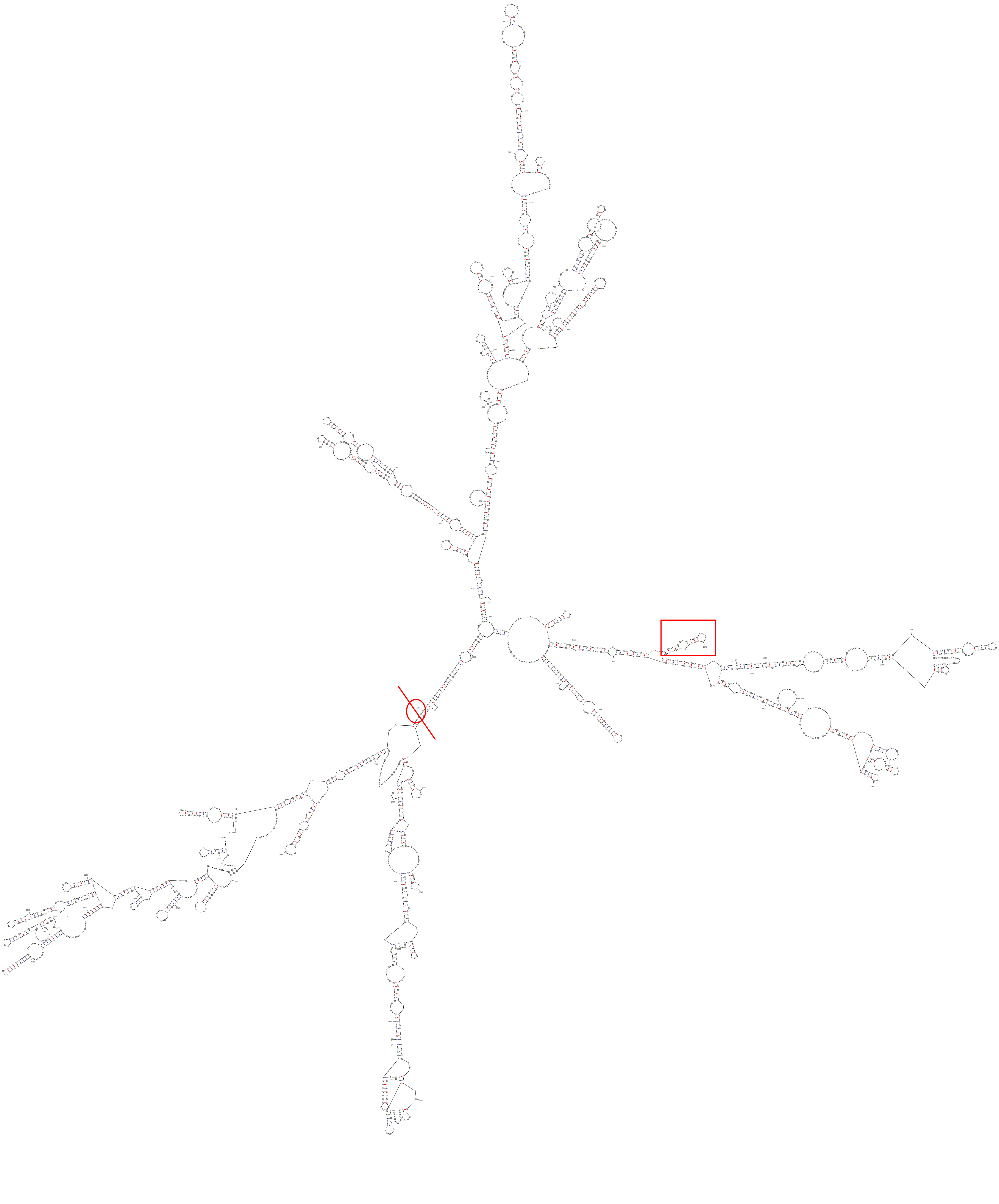

dG = -683.46 [Initially -764.30] human-full

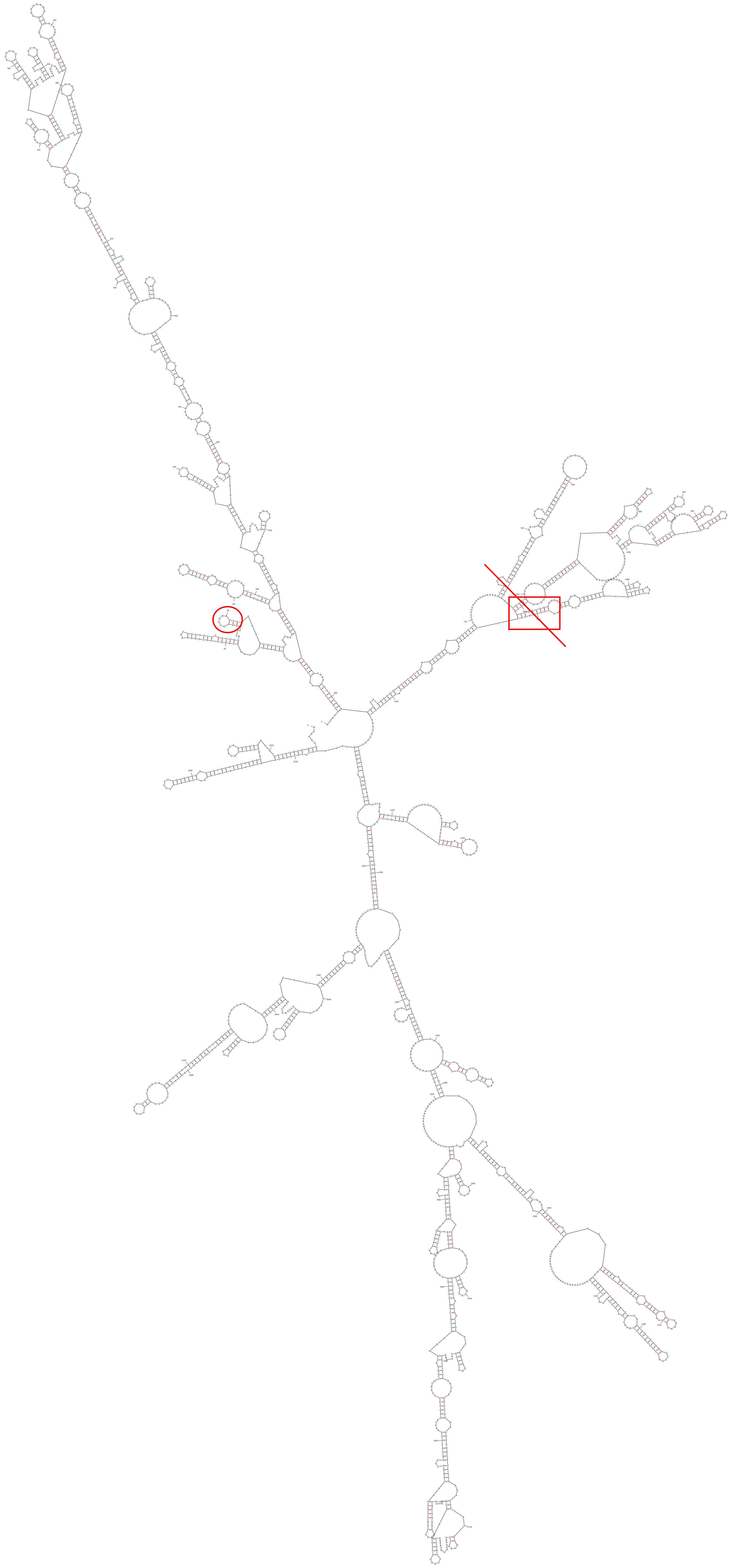

dG = -669.60 [Initially -760.90] human-full

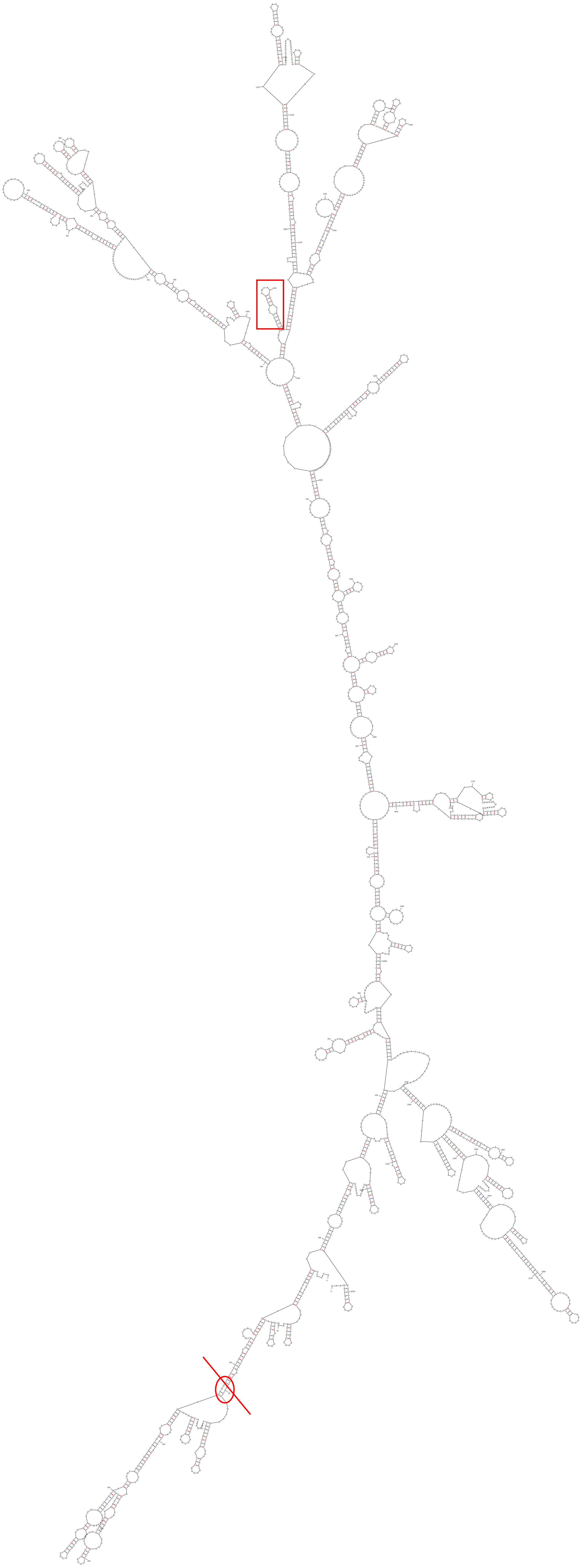

dG = -669.88 [Initially -760.30] human-full

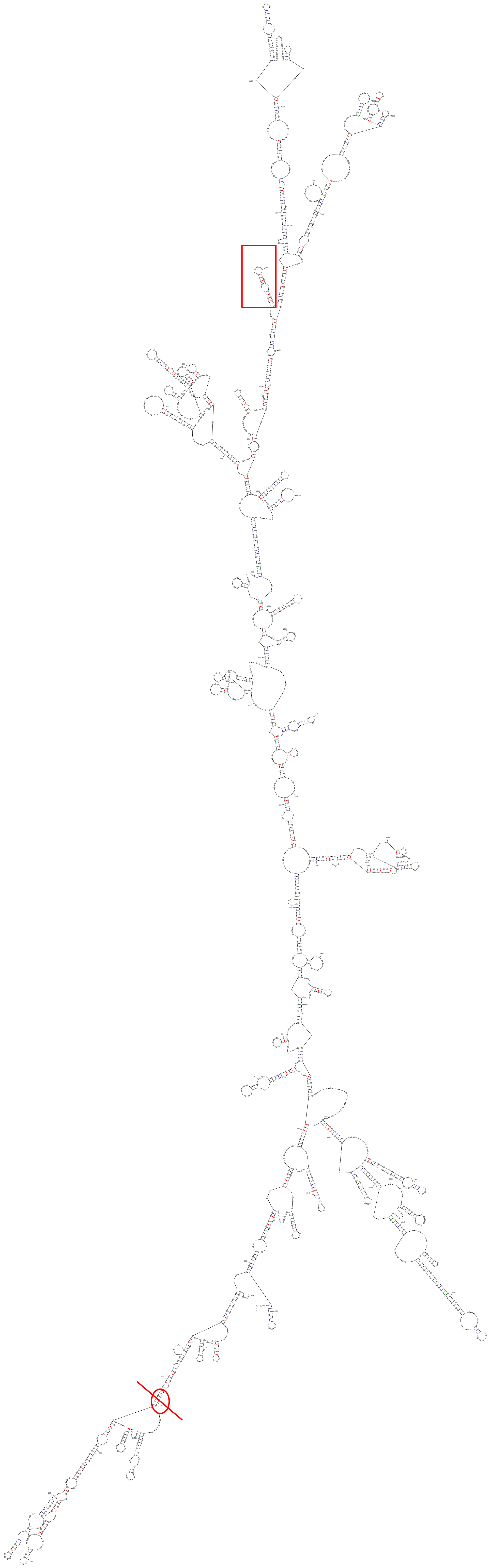

dG = -656.84 [Initially -759.90] human-full

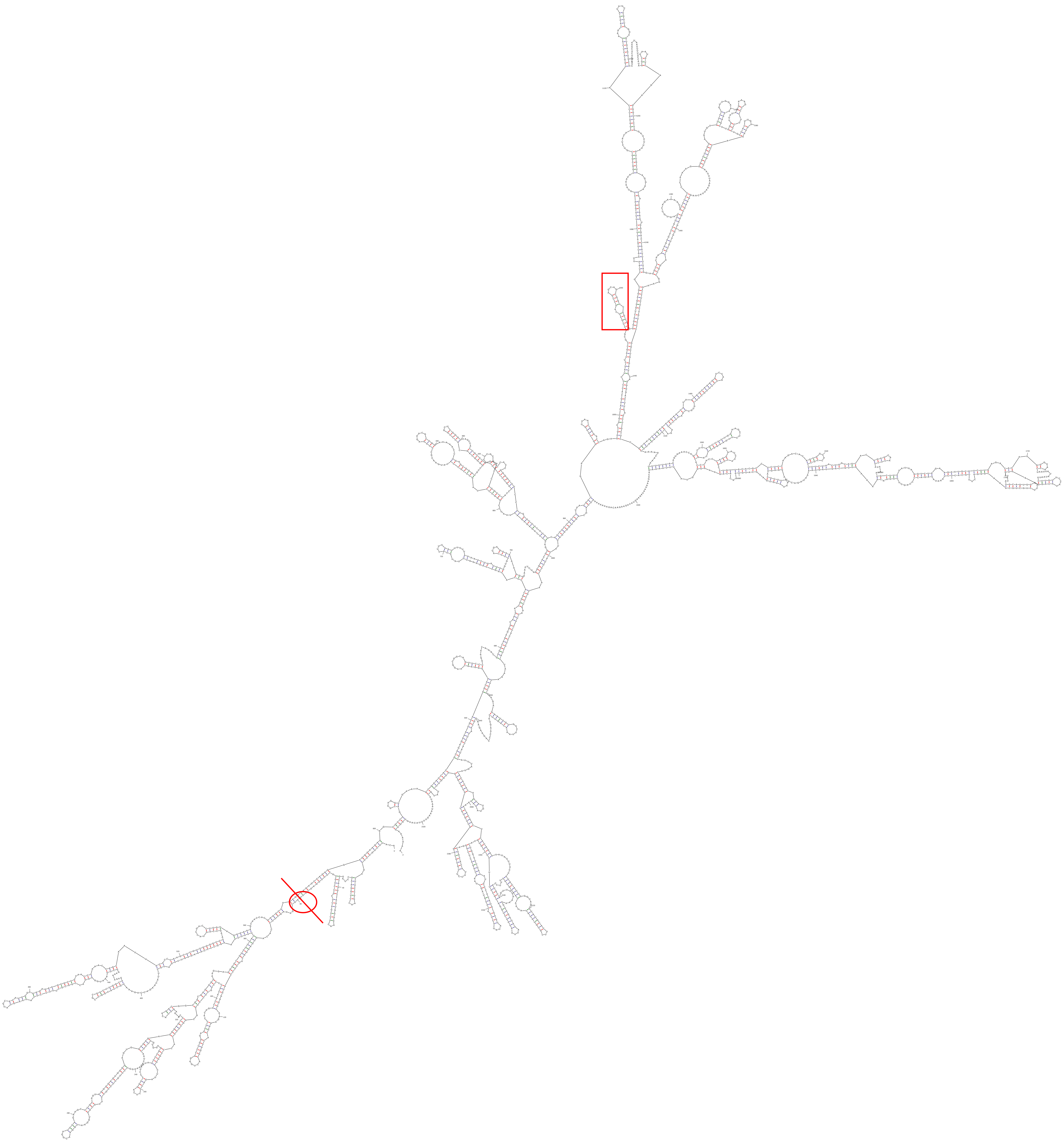

dG = -670.66 [Initially -758.10] human-full

Output of sir_graph (©)
mfold_util 4.6

Created Thu Mar 10 19:49:04 2011

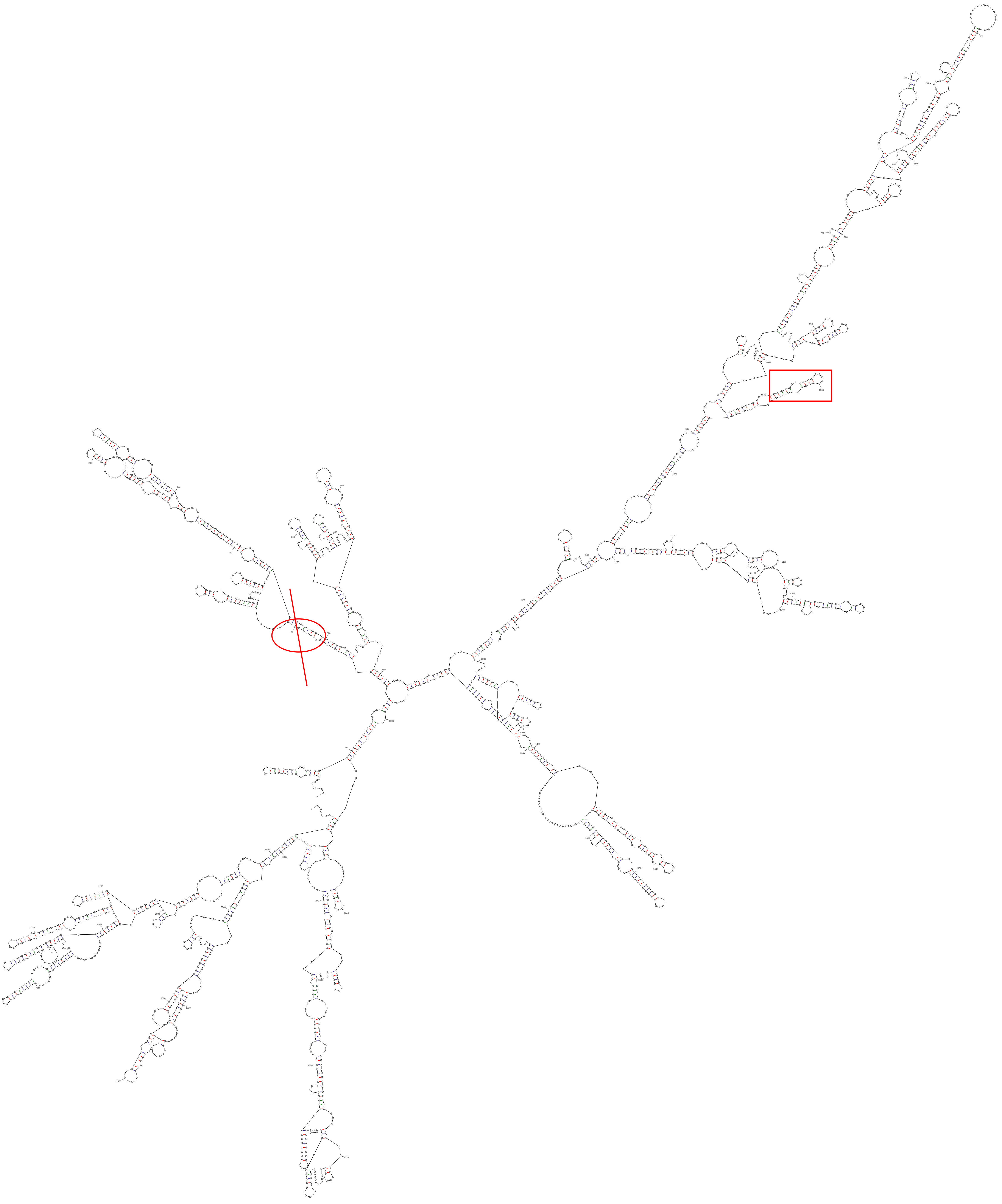

dG = -679.70 [Initially -771.00] human-full

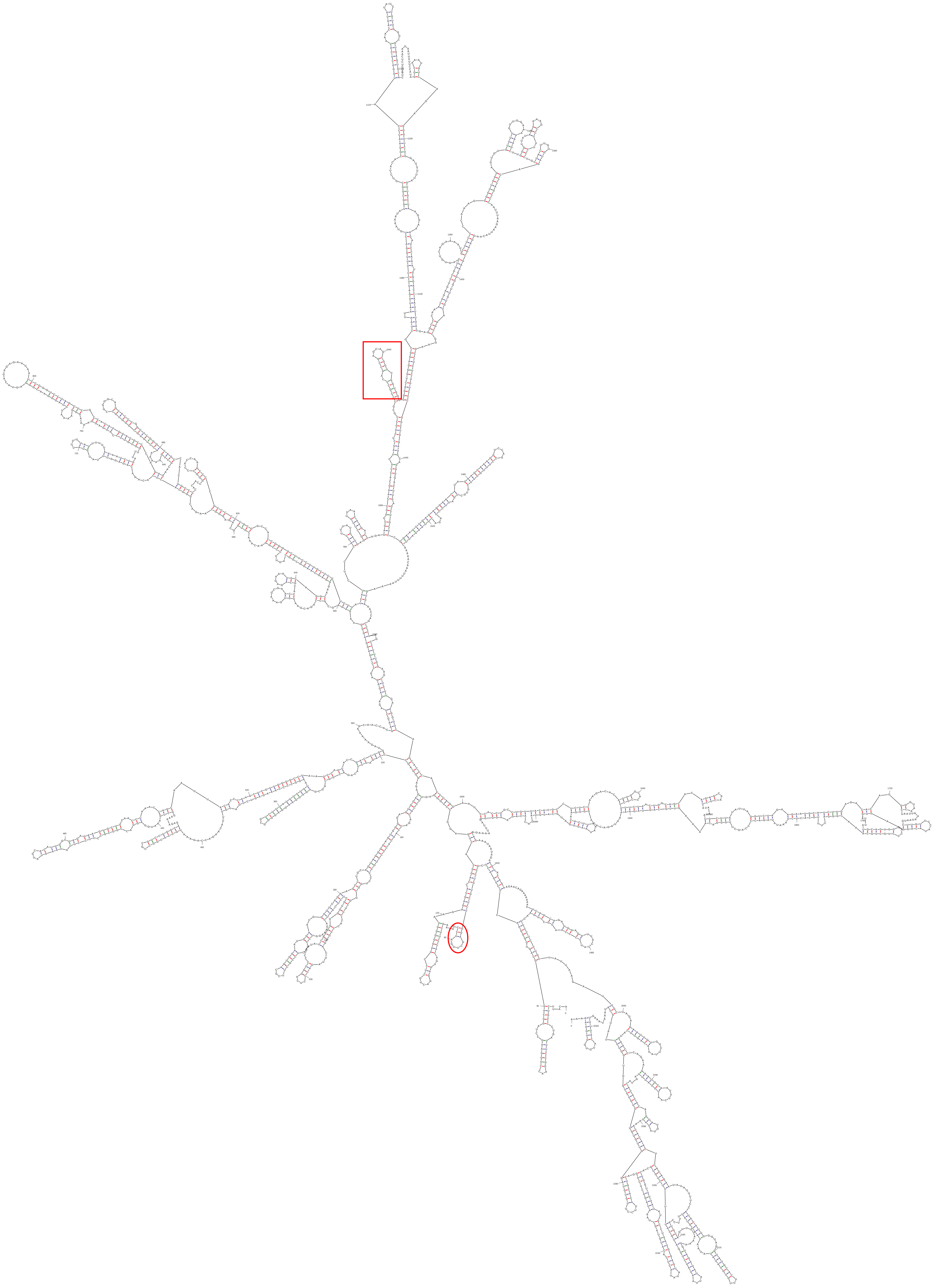

dG = -683.68 [Initially -771.00] human-full

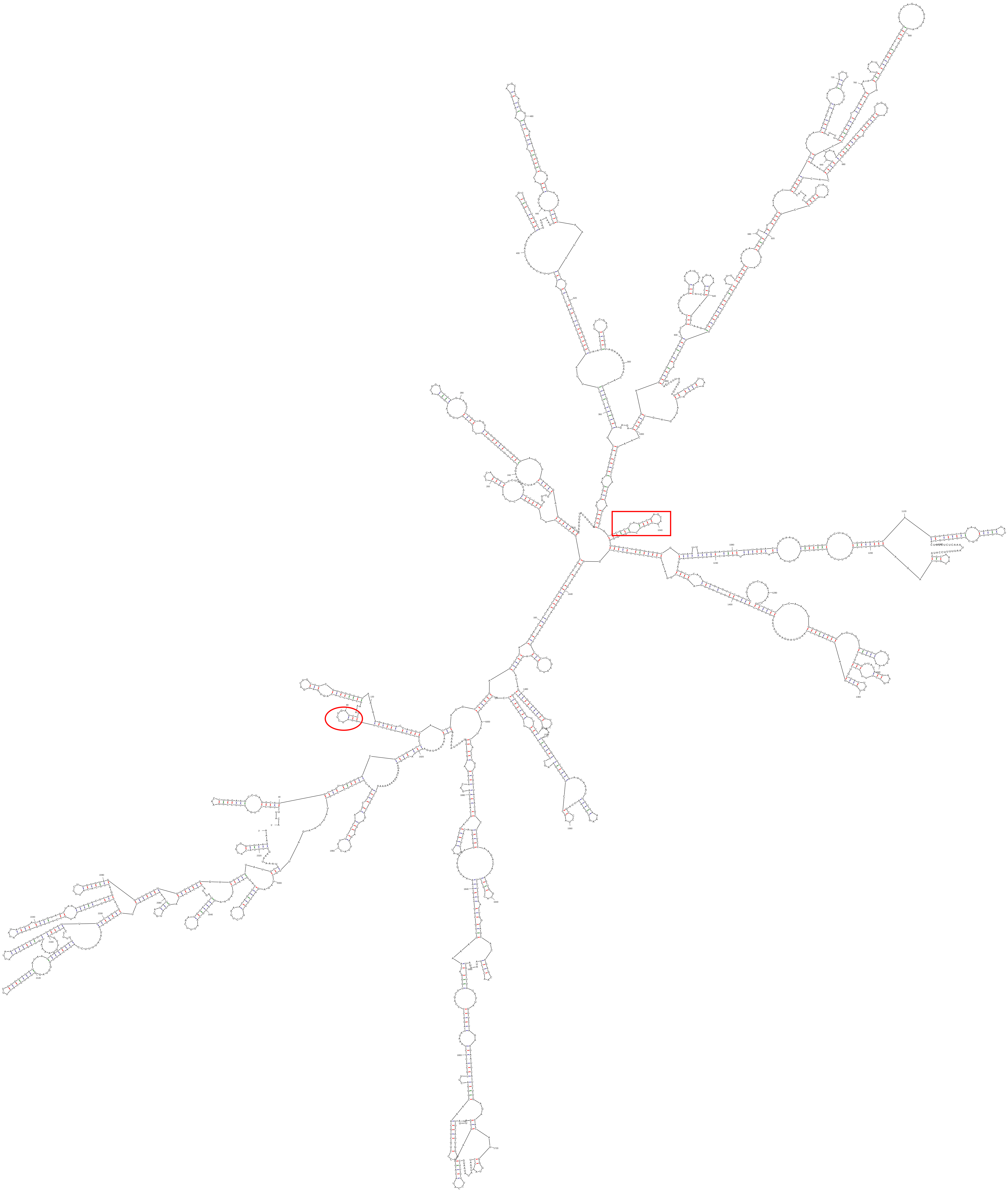

dG = -681.56 [Initially -770.40] human-full

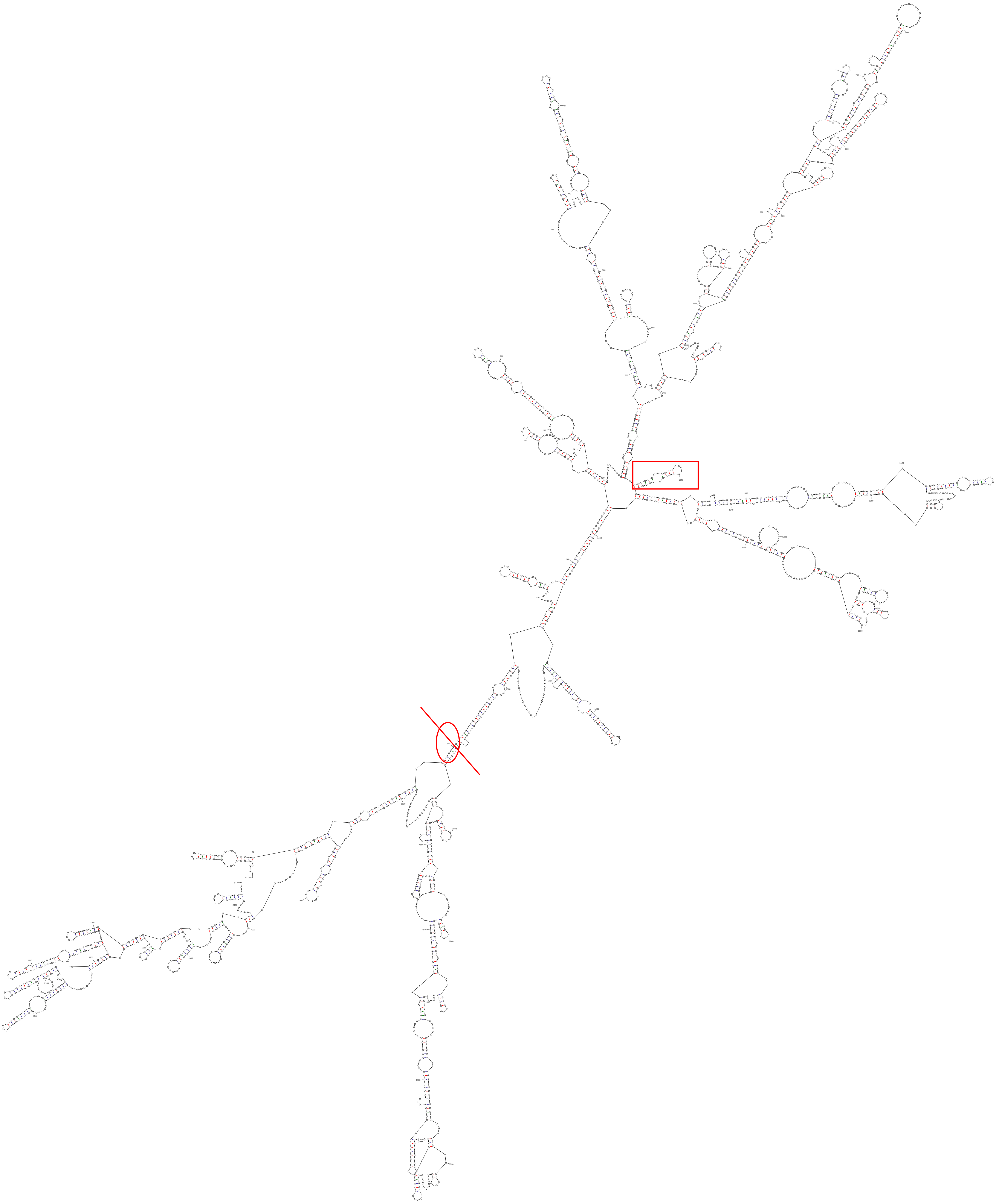

dG = -684.05 [Initially -769.70] human-full

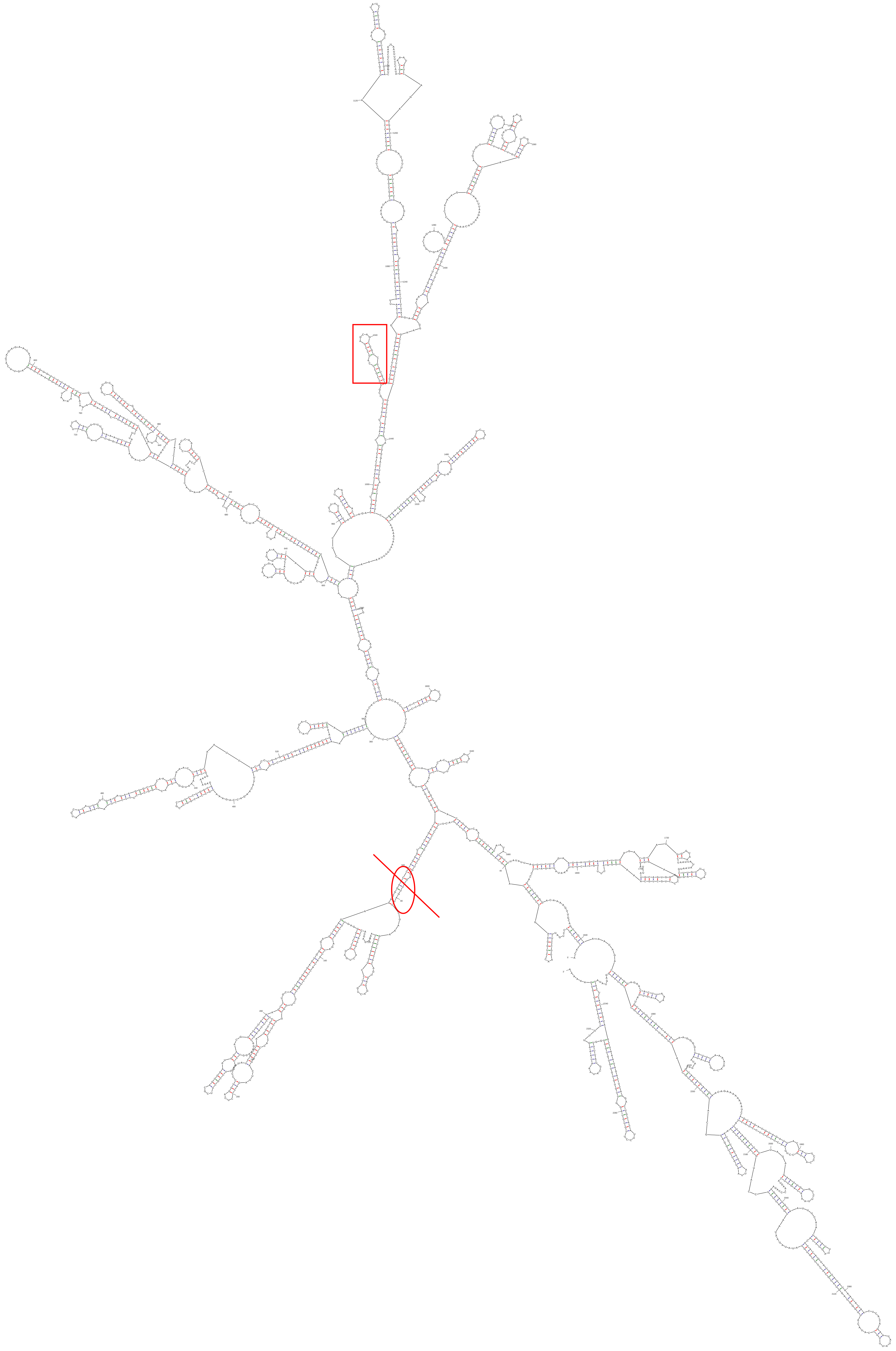

dG = -686.05 [Initially -769.70] human-full

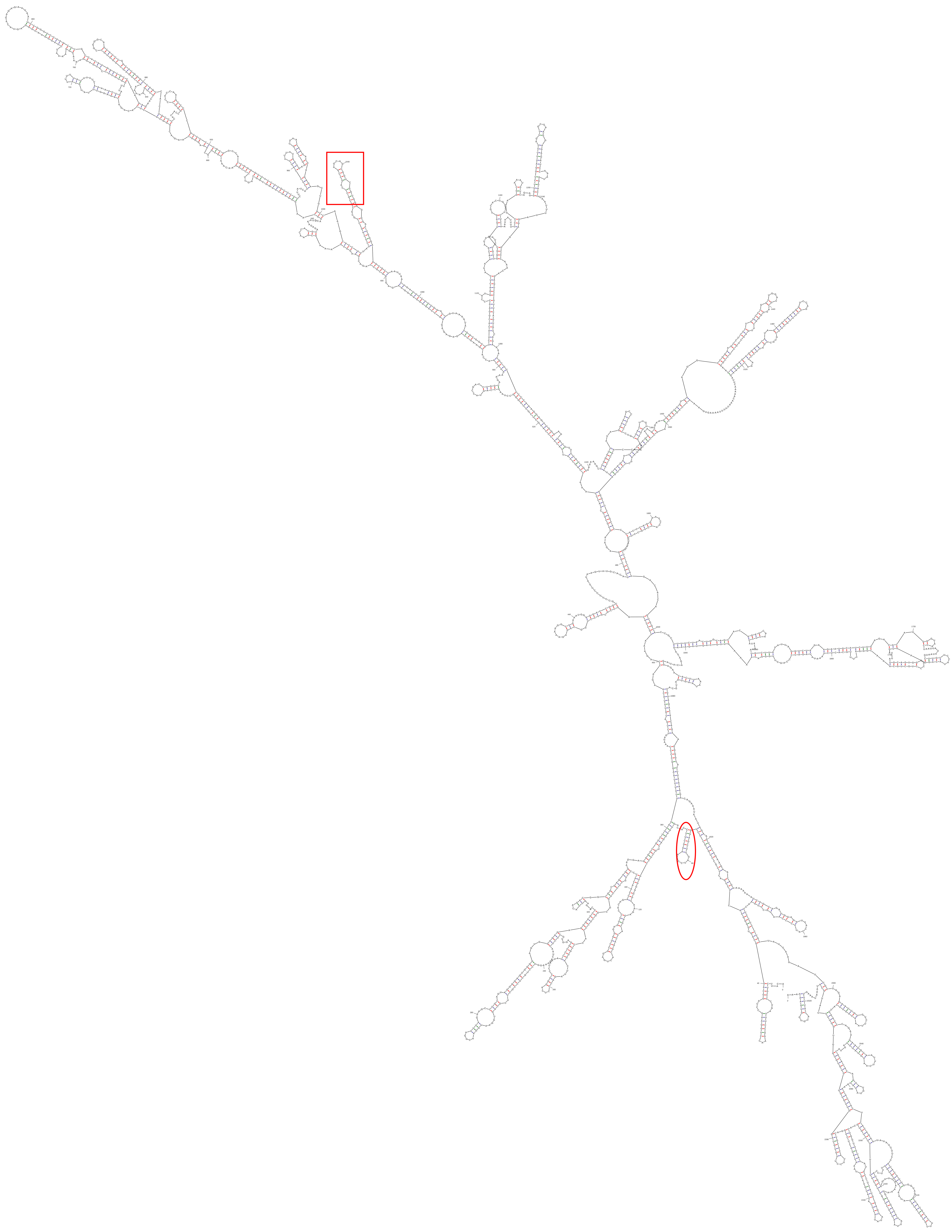

dG = -672.88 [Initially -769.50] human-full

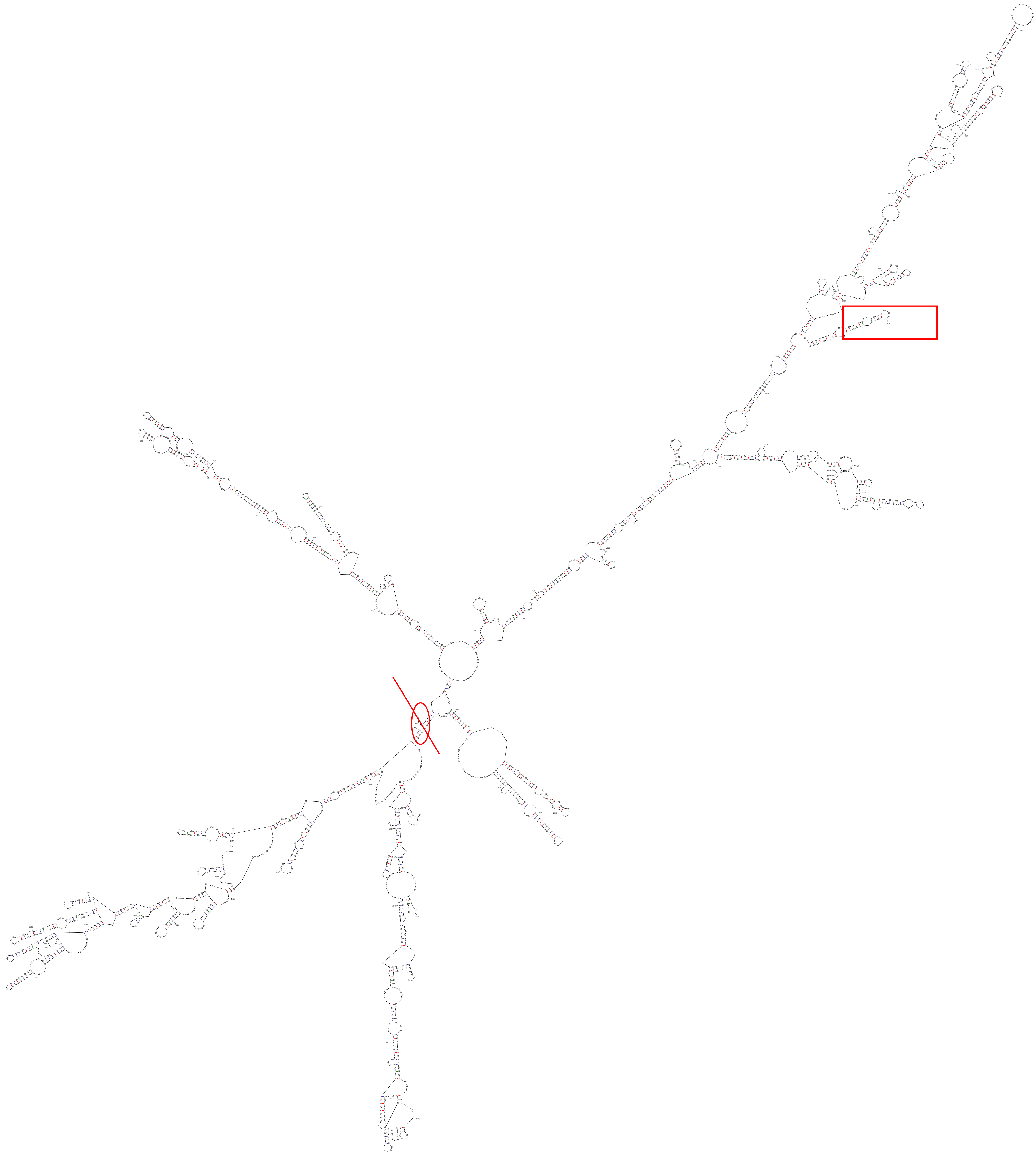

dG = -670.13 [Initially -768.90] human-full
